# Training-induced circuit-specific excitatory synaptogenesis in mice is required for effort control

**DOI:** 10.1038/s41467-023-41078-z

**Published:** 2023-09-08

**Authors:** Francesco Paolo Ulloa Severino, Oluwadamilola O. Lawal, Kristina Sakers, Shiyi Wang, Namsoo Kim, Alexander David Friedman, Sarah Anne Johnson, Chaichontat Sriworarat, Ryan H. Hughes, Scott H. Soderling, Il Hwan Kim, Henry H. Yin, Cagla Eroglu

**Affiliations:** 1 Department of Cell Biology, Duke University Medical Center, Durham, NC 27710 USA; 2 Department of Psychology and Neuroscience, Duke University, Durham, NC 27710 USA; 3 Department of Neurobiology, Duke University Medical Center, Durham, NC 27710 USA; 4 Duke Institute for Brain Sciences (DIBS), Durham, NC 27710 USA; 5 Department of Anatomy & Neurobiology, University of Tennessee Health and Science Center, Memphis, TN 38103 USA; 6 Howard Hughes Medical Institute, Duke University, Durham, NC 27710 USA; 7 Present Address: Cajal Institute (CSIC), Madrid, 28001 Spain

**Keywords:** Cellular neuroscience, Synaptic plasticity, Neuronal physiology, Neural circuits, Motivation

## Abstract

Synaptogenesis is essential for circuit development; however, it is unknown whether it is critical for the establishment and performance of goal-directed voluntary behaviors. Here, we show that operant conditioning via lever-press for food reward training in mice induces excitatory synapse formation onto a subset of anterior cingulate cortex neurons projecting to the dorsomedial striatum (ACC_→DMS_). Training-induced synaptogenesis is controlled by the Gabapentin/Thrombospondin receptor α2δ−1, which is an essential neuronal protein for proper intracortical excitatory synaptogenesis. Using germline and conditional knockout mice, we found that deletion of α2δ−1 in the adult ACC_→DMS_ circuit diminishes training-induced excitatory synaptogenesis. Surprisingly, this manipulation does not impact learning but results in a significant increase in effort exertion without affecting sensitivity to reward value or changing contingencies. Bidirectional optogenetic manipulation of ACC_→DMS_ neurons rescues or phenocopies the behaviors of the α2δ−1 cKO mice, highlighting the importance of synaptogenesis within this cortico-striatal circuit in regulating effort exertion.

## Abstract (web summary)

The role of synaptogenesis during the acquisition of goal-directed behaviors is unknown. Here, the authors show that learning-induced synaptogenesis in the adult mouse cortex is required to excite a specific circuit to adjust effort exertion.

## Introduction

Goal-directed behaviors are executed to obtain desirable outcomes, such as food rewards. These complex voluntary behaviors are established when motivated individuals learn to associate a set of actions with its desirable outcome^1^. An important aspect of goal-directed behaviors is effort, which can be described as the motor and cognitive resources allocated to action performance. Learned behaviors need to be adaptable to changing action-outcome contingencies because, often, the effort required to reach the desired outcome changes. Spending too much effort on ineffective or excessively demanding actions can be maladaptive and detrimental to survival^2,3^.

Synapses are the smallest units of neuronal circuits that control behaviors. The molecular mechanisms regulating synaptogenesis are highly complex. Importantly, mutations in genes encoding for synaptic or synaptogenic proteins are linked to a large number of brain diseases^4^ with severe cognitive decline, sensorimotor and memory deficits^5–12^. The majority of the synaptic structures are established during development; however, synaptic connectivity is not stagnant and is dynamically modified throughout the life span^13–15^. This experience-dependent remodeling of synapses is thought to underlie cognitive processes such as learning and memory^16–21^.

Long-term synaptic plasticity is widely accepted as the mechanism underlying synaptic remodeling associated with learning^21–24^. However, whether synaptogenesis is involved in the learning and performance of complex behaviors is less studied. In this study, in freely moving animals, we investigated whether new synapse formation occurs during training and if it is required for learning reward-based behaviors. To do so, we used an instrumental operant-conditioning task in mice^25,26^. In this behavioral paradigm, a mouse first learns to perform a specific action (i.e., press a lever) to earn a reward (i.e., food pellet). Once the mouse associates the action and outcome, the contingency can be changed either by increasing the number of lever presses required to receive the reward or by manipulating the relationship between lever press and reward. The mice adapt their rate of lever pressing based on these changing contingencies. For example, mice increase their effort when the number of presses required to achieve a reward is increased, but they would diminish the lever press rate if the task becomes too demanding^27,28^.

Previous research utilizing similar operant-conditioning paradigms identified brain regions within the basal ganglia circuits responsible for controlling instrumental actions and revealed how disruption of these circuits affects learning and performance^29–31^. For example, learning the action-outcome relationship requires the dorsomedial striatum (DMS)^29,32^, a hub for many cortical inputs^33,34^. Particularly, the inputs coming from the prefrontal cortex (PFC) to the DMS play critical roles in instrumental training, such as learning the action-outcome contingency^31,35,36^, evaluation of an outcome’s value^30,37,38^, and deciding between different actions or outcomes^39,40^. However, the cellular and molecular mechanisms underlying the remodeling of these cortico-striatal circuits during learning and how their functions control goal-directed actions are unknown. Closing these fundamental knowledge gaps is needed to determine druggable molecular targets to treat brain disorders, such as obsessive-compulsive disorder (OCD), autism spectrum disorder (ASD), and Alzheimer’s disease (AD), in which goal-directed behaviors are impaired^41–44^.

Which cortical regions control learning and establishment of instrumental behaviors? Does training induce cortical synaptogenesis? Are the newly formed synapses required for the establishment of learned goal-directed actions? Here, we addressed these questions by applying molecular and anatomical tools, circuit-specific gene modifications, optogenetics and quantitative behavioral techniques. By investigating the immediate early gene c-Fos expression as a marker for structural synaptic plasticity and neuronal activity^45–48^, we found that the Anterior Cingulate Cortex (ACC) is significantly activated by instrumental training. This knowledge allowed us to identify an ACC_→DMS_ connection controlling instrumental behavior performance.

In humans, the PFC, a brain region containing the ACC, is associated with cognitive control, the ability to flexibly adjust our behaviors and allocate effort towards our goals^49–52^. In rodents, the ACC has been suggested to regulate learning^53^, reward and action monitoring^54^, effort-based decision-making^55–57^, impulsivity^58^, and selection of behavioral strategy^59^ because the neuronal activity in the ACC correlates with these aspects of complex behaviors. However, the cellular and molecular mechanisms utilized by the ACC circuits to achieve such roles are unknown. To determine if new synapse formation within the ACC_→DMS_ circuit we identified is critical for the establishment and performance of goal-directed actions, we took a genetic approach to block synaptogenesis specifically in these neurons. To do so, we targeted the Gabapentin receptor α2δ−1, a type-I membrane protein encoded by *Cacna2d-1*^60^.

α2δ−1 is essential for the proper formation and maturation of intracortical excitatory synapses during development^61^. α2δ−1 (*Cacna2d-1*) is also highly expressed in the adult mouse cortex^62^. It was first identified as a subunit of voltage-gated calcium channels^63^. Then, it was shown to interact with several synaptic and synaptogenic proteins^60,64^, including the astrocyte-secreted Thrombospondins (TSPs)^61,65^. The synaptogenic function of α2δ−1 is mediated by Rac-1-signaling within the dendrites of cortical neurons^61^, and it is independent of its role in calcium channel trafficking^65–67^. Despite its known critical functions in the development and maturation of synapses, whether α2δ−1 controls adult synaptogenesis and contributes to the learning and performance of complex behaviors remains poorly understood. Here, we show that α2δ−1-dependent adult synaptogenesis as a cellular mechanism controlling the adaptability of voluntary goal-directed actions. Surprisingly, training-induced synapse formation is not required for learning but for the control of effort exertion through action sequence modulation.

## Results

### Lever press task induces the establishment of lever press bouts and increases immediate early gene expression in the ACC

**Fig. 1:**
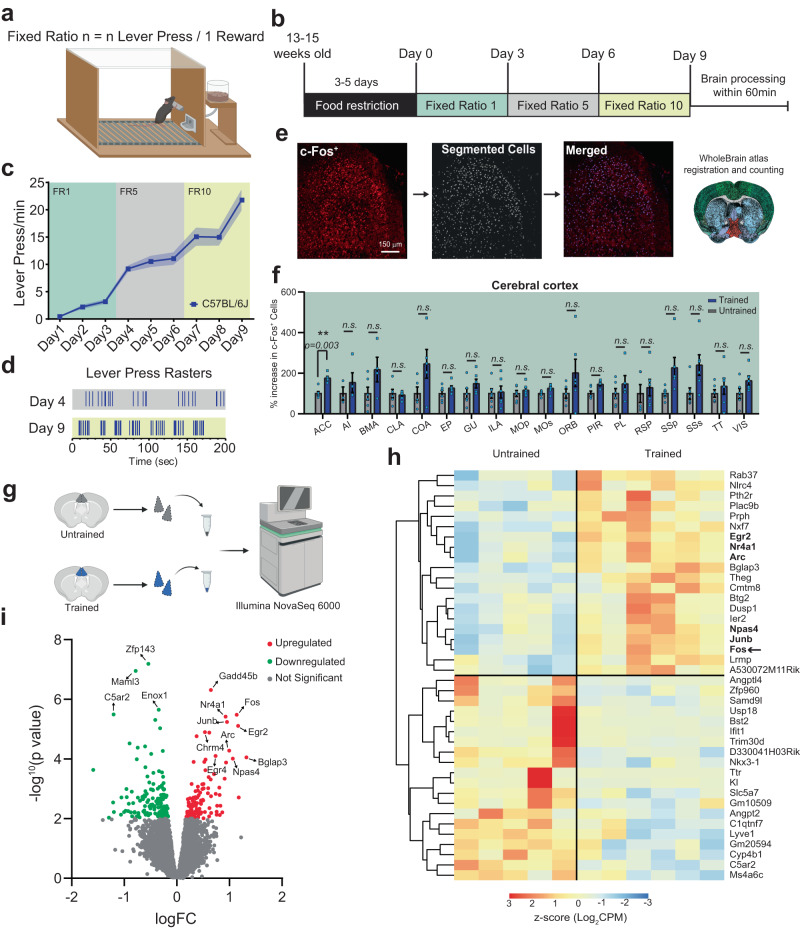
Action sequence engages the anterior cingulate cortex during instrumental actions. **a** Representation of the Skinner box used for training and testing mice on a lever-press-for-food reinforcer test. **b** Schematic of the fixed ratio schedules used for the operant training and testing. **c** Lever press (LP) rate for trained *C57BL/6J* mice (*n* = 17 mice, 8 males, and 9 females) across the 9 days of training. **d** Representative lever press raster plots for FR5 day 4 and FR10 day 9 of LP performance. **e** Flow chart and example images of the c-Fos^+^ cell segmentation and quantification. **f** Bar plot of c-Fos^+^ cells for the cerebral cortex regions. **g** Schematic representation of the ACC microdissection procedure used for the bulk RNA-seq. **h** Heat map of the top 20 differentially expressed genes (DEG). Bolded genes are the IEGs overexpressed in Trained mice. **i** Volcano plot organized based on logarithmic fold change (logFC) and *P* value (−log_10_*P* value). Arrows signify genes of interest. Source data are provided as a Source Data file. Drawings in (**a**, **g**) are created with BioRender.com.

To model operant behaviors in mice, we used an instrumental conditioning task in which mice learn to press a lever for a food reward. In this paradigm, mice first learn the relationship between an action (lever press, LP) and the desired outcome (food reward). Following this initial phase, the animal’s performance can be improved by increasing the number of LPs required for each reward (fixed ratio, Fig. 1a).

We trained male and female wild-type (WT, *C57BL/6J*) mice using a fixed ratio (FR) schedule to identify brain regions responsible for the learning and performance of the LP task. Mice were first trained on an FR1 schedule (1 lever press (LP)/1 reward, days 1–3), then on an FR5 (5 lever presses/1 reward, days 4–6), and finally on an FR10 schedule (10 lever presses/1 reward, days 7–9) (Fig. 1b, see “Methods” for details). An untrained control group (age- and sex-matched) was also food-restricted and housed in the same training chamber for an equivalent number of days and durations as the trained mice. However, the untrained mice were given free access to the same amount of food rewards without pressing the lever.

Throughout the training, the mice learned to press the lever efficiently, which is reflected by the significant increase in the number of LPs/min between the first day of training (Day 1-FR1) compared to the last day (Day 9-FR10) (Fig. 1c). Apart from the increased number of LPs/min, we utilized the probability distribution of inter-press intervals (IPI, Supplementary Fig. 1a) to identify and analyze the lever press bouts across days of training as a parameter of improved task performance (Fig. 1d, top and Supplementary Fig. 1b). Quantification of bout duration, number of LPs per bout, mean inter-press interval (IPI) within each bout and inter-bout interval (IBI) from day 4 through day 9 show an overall improvement of performance over time (Supplementary Fig. 1c–f). We did not find any sex differences in the performance of this task (Supplementary Fig. 1g). These data show that in this paradigm, mice of either sex learn the action-outcome contingency and efficiently adapt to contingency changes (i.e., transitioning to FR5 and FR10) by performing bouts of a discrete number of LPs.

Which brain regions are critical for learning and performance of the lever press task? To answer this question, we first analyzed the immediate early gene (IEG) c-Fos expression as a cellular marker of increased neuronal activity in several brain regions^46,68^ after the FR10 schedule. To capture the c-Fos protein at its highest levels^46^, we processed the brains of the trained and untrained mice within 60 min after completing the last session on day 9 (Fig. 1b). Coronal brain sections from trained and untrained mice corresponding to 4 forebrain Bregma coordinates (Supplementary Fig. 1h, posterior–anterior: −0.3; +0.4; +1.7; +2.2 from *The Mouse Brain in Stereotaxic Coordinates, Franklin and Paxinos 2008*) were stained for c-Fos and DAPI (nuclear DNA marker). The c-Fos^+^/DAPI^+^ cells (hereafter named c-Fos^+^) were imaged from the entire coronal brain sections and segmented using the U-Net machine-learning algorithm (https://github.com/ErogluLab/CellCounts, see “Methods” for details). With the Whole Brain Software^69^, we mapped the segmented c-Fos^+^ cells onto the Allen brain atlas (Supplementary Table 1) for the corresponding Bregma coordinates (Fig. 1e and Supplementary Fig. 1i). This analysis revealed a significant increase in the total number of c-Fos^+^ cells in trained compared to untrained mice (Supplementary Fig. 1j). The 42 detected brain regions were grouped based on their classification as regions belonging to the cerebral cortex (Fig. 1f), cerebral nuclei (Supplementary Fig. 1k), or interbrain (Supplementary Fig. 1l). A significant increase in c-Fos^+^ cells after training was detected in the Anterior Cingulate Cortex (ACC) and the Magnocellular Nucleus (MA). Previous work has implicated the prefrontal cortex in the learning and performance of instrumental actions, decision-making in cost-benefit tasks, and behavioral flexibility^31,39,40,59,70–72^. We, therefore, focused on the ACC for the remainder of our study because our findings suggested that the ACC, among the prefrontal cortex regions, is strongly and selectively activated by instrumental training.

To further investigate and confirm the changes in immediate-early gene expression due to the training, we micro-dissected the ACC from trained and untrained mice and performed RNA-sequencing (RNA-seq) (Fig. 1g). Of the 75 differentially expressed genes (DEGs) identified, 32 were significantly upregulated in the ACC of trained mice, and 43 were downregulated (i.e., 1.5-fold change compared to untrained animals, at a nominal *P* value of *P* < 0.01, Fig. 1h, i).

We found several immediate early genes (IEGs) among the genes upregulated by training, such as *Fos, Jun, Npas4, Arc, Nr4a1*, *Egr2*, and *Egr4* (Fig. 1h, i), known for their role in synaptic plasticity and memory-related processes^73–75^. Indeed, GO term analyses of the upregulated genes showed a significant (adjusted *P* value ≤ 0.05) enrichment for genes involved in cognitive processes and long-term memory (Supplementary Fig. 1m). Although not statistically significant, we found genes involved in other relevant categories in the Cellular Component and Molecular Functions groups. These are plasma membrane bounded cell projection cytoplasm, which indicates those genes involved in the elongation of processes from the cell body (i.e., axons, dendrites) (Supplementary Fig. 1n); and RNA polymerase II-specific DNA-binding transcription factor binding category which is also relevant for the long-term changes related to cognitive function (Supplementary Fig. 1o).

The KEGG pathway analysis (Supplementary Fig. 1p) resulted in only one category highly represented in terms of gene counts, the MAPK signaling pathway. This pathway is relevant to synaptic plasticity because, in mature neurons, it leads to the activation of extracellular signal-regulated kinases (ERKs), which are induced by excitatory glutamatergic signaling and possibly relevant to synaptic plasticity^76,77^.

These results reveal increased IEG expression in the ACC of trained mice, providing evidence for heightened neuronal activity in this region during the performance of the LP task. Furthermore, because IEGs are involved in the molecular mechanisms underlying long-term structural and functional changes in synaptic circuits during learning and memory^73,75,78^, these findings suggest that LP training induces long-term circuit remodeling in the ACC.

### Excitatory synapses increase in the ACC after training

**Fig. 2:**
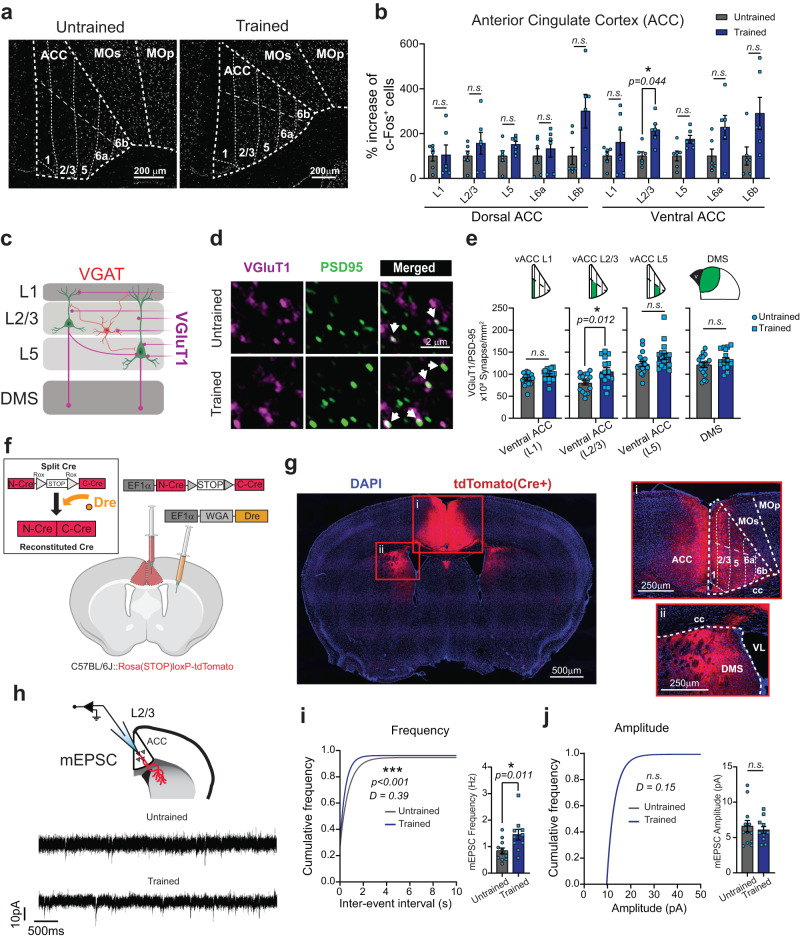
A high ratio schedule induces excitatory synaptogenesis in the Anterior Cingulate Cortex. **a** Example images of the segmented c-Fos^+^ cells across the layers of the ACC in untrained and trained groups. **b** Layer-specific quantification of the c-Fos^+^ cells in the dorsal and ventral ACC in trained and untrained *C57BL/6J* mice. **c** Representation of the specific cortical layers in which synaptic analysis was performed for VGlut1 and VGAT synapses (created with BioRender.com). **d** Representative images from Untrained and Trained mice stained with VGluT1 and PSD95 antibodies. The *arrows* indicate co-localized puncta. **e** Quantification of VGluT1/PSD95 co-localized puncta density in the ACC and DMS. **f** Schematic representation of the viral injections to label ACC_→DMS_ neurons (created with BioRender.com). **g** Tile scan image of a coronal brain section from a *C57BL/6* *J* tdTomato^+^ mouse. Insets show a magnification of the ACC (i) and DMS (ii). **h** Schematic representation of mEPSC recording from ACC_→DMS_ neurons and representative traces. **i** Cumulative distribution and bar plots of inter-event interval. **j** Cumulative distribution, and bar plots of amplitude. Source data are provided as a Source Data file.

Closer inspection of the c-Fos^+^ cells in the ACC revealed that instrumental training significantly increases the number of c-Fos^+^ cells in layers 2/3 of the ventral ACC (vACC, Fig. 2a, b). The ACC layers 2/3 and 5 contain neurons that project to the DMS, a striatal region that controls instrumental actions and action sequences^33,79,80^. In contrast, there were no significant differences in the numbers of c-Fos^+^ cells between trained and untrained mice in the dorsal ACC (Fig. 2a, b) and the neighboring secondary Motor Cortex (MOs), which also sends projections to the DMS (Supplementary Fig. 2a). These results show that there is a significant increase in the numbers of c-Fos^+^ cells in the L2/3 of the vACC of trained mice and implicate the ACC neurons that project to the DMS as neurons that have increased activity after training.

We postulated that training promotes a net increase in excitatory synapses made onto the neurons in this region, which results in increased neuronal activity and a consequential increase in IEGs expression. To test this hypothesis, we acquired high-magnification confocal images of mouse brain sections stained for specific markers of excitatory or inhibitory synaptic structures in the L2/3, L5, and the synaptic zone (layer 1, L1) of the vACC from trained and untrained mice. The synaptic zone in L1 harbors the apical dendrites from L2/3 and L5 neurons, which receive many synapses. In addition, we analyzed synapse numbers in the DMS, the axonal target for ACC neurons (Fig. 2c). To visualize and quantify structural synapses, we used an established protocol^81^ that marks synapses as the juxta-positioning of pre and postsynaptic markers. This method takes advantage of the close proximity of pre and postsynaptic markers and of the resolution limit of light microscopy. The pre and postsynaptic proteins are in distinct neuronal compartments (axons and dendrites, respectively); however, due to their close proximity at synapses, they appear partially co-localized.

We used the Vesicular Glutamate Transporter 1 and the Post-Synaptic Density protein 95 (VGluT1/PSD95) or the Vesicular GABA Transporter and gephyrin (VGAT/Gephyrin) to mark the respective pre and postsynaptic compartments of excitatory or inhibitory synapses (Fig. 2d and Supplementary Fig. 2e). We found an increase in the VGluT1/PSD95-positive excitatory synapse density in the L2/3 of the vACC of trained mice compared to untrained controls (Fig. 2e). We excluded that the observed increase was due to an enlargement of the pre or postsynaptic side as we found no changes in the puncta size after training (Supplementary Fig. 2c). When we quantified the number of VGluT1 or PSD95 puncta individually (Supplementary Fig. 2d), we found no correlations with the synapse number changes, suggesting that neither pre nor postsynaptic alterations alone can account for the training-induced synaptogenesis. Moreover, no significant changes in synapse densities were observed in the vACC L1, L5 or the DMS (Fig. 2e), or within the cortical region adjacent to ACC, the MO (Supplementary Fig. 2b). Finally, training did not alter the numbers of VGAT/Gephyrin-positive inhibitory synapses in any of these regions (Supplementary Fig. 2f). These data show that the training and improved performance of instrumental actions is accompanied by a significant increase in the numbers of VGluT1/PSD95-positive excitatory synapses in the L2/3 of the vACC.

Next, we tested whether the increase in the number of structural excitatory synapses we observed reflects a net functional increase in excitatory inputs onto the ACC neurons projecting to the DMS (hereafter called ACC_→DMS_ neurons). To do so, we recorded miniature excitatory postsynaptic currents (mEPSCs) from ACC_→DMS_ neurons of the trained and untrained mice. To label ACC_→DMS_ neurons, we used a viral approach that relies on the combined functions of two adeno-associated viruses (AAVs)^82^. The first AAV is injected bilaterally into the DMS and expresses the Dre-recombinase protein, a site-specific recombinase like Cre with a specific target site called *rox*^83^, conjugated to Wheat Germ Agglutinin (WGA). WGA is a lectin that retrogradely transports across synapses^84^. Hence, WGA-conjugated Dre-recombinase is expressed by DMS neurons and transferred to all the neurons that synapse onto the DMS. A second AAV is bilaterally injected into the ACC and contains the Cre-recombinase coding sequence; however, Cre protein expression is interrupted by a Rox-flanked STOP cassette (N-Cre-*rox-STOP-rox*-C-Cre, Fig. 2f). This strategy ensures that Cre is only expressed in the ACC_→DMS_ neurons, because the retrogradely transported WGA-Dre allows the recombination of the rox-STOP-rox codon within the Cre-recombinase (Fig. 2f). We verified the efficiency and specificity of this approach to target ACC_→DMS_ neurons using a Cre-reporter mouse line, Rosa (STOP)loxP-tdTomato^85^ (Fig. 2g).

This approach allowed us to perform mEPSC recordings specifically in the L2/3 ACC_→DMS_ neurons (Fig. 2h) in trained and untrained mice. In line with an increase in the number of excitatory synapses, we found a significant increase in the frequency, but not in the amplitude, of mEPSCs in the L2/3 ACC_→DMS_ neurons of the trained mice compared to untrained controls (Fig. 2i, j). These findings strongly suggest that operant training induces excitatory synaptogenesis onto ACC_→DMS_ neurons. These findings also implicate excitatory synapse formation as a necessary step for the learning and performance of instrumental actions.

### Training-induced excitatory synapse formation in the ACC requires the synaptogenic neuronal receptor α2δ−1

**Fig. 3:**
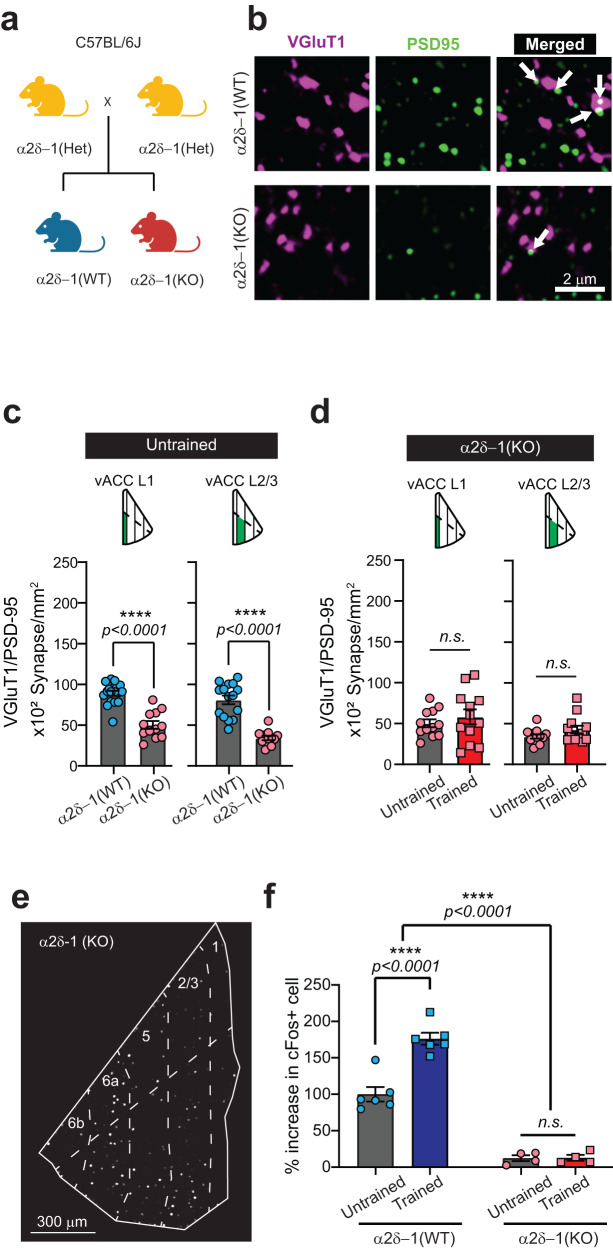
VGlut1-PSD95 synapse formation in the anterior cingulate cortex is regulated by the thrombospondin receptor α2δ−1. **a** Breeding scheme for the generation of α2δ−1 WT and KO mice (created with BioRender.com). **b** Representative images of VGluT1/PSD95 staining in the ACC of α2δ−1 WT and KO mice. The *arrows* in the merged channel indicate co-localized puncta. **c** Comparison between untrained α2δ−1 WT and KO mice. **d** Layer-specific comparison of untrained and trained α2δ−1 KO in the ventral ACC. **e** Example image of the segmented c-Fos^+^ cells across the layers of the ACC of a trained α2δ−1 KO mouse. **f** Quantification of the c-Fos+ cells in untrained and trained α2δ−1 KO mice compared to WT. Source data are provided as a Source Data file.

Next, we took a genetic approach to determine if training-induced synapse formation is required for instrumental learning and performance. *Cacna2d1* encodes for α2δ−1, the neuronal receptor for the astrocyte-secreted synaptogenic TSPs, and the anti-epileptic drug Gabapentin. α2δ−1 is required for intracortical synaptogenesis during development in the sensory cortical regions^61^. By mining single-cell RNA sequencing data from adult mouse brain^86^, we found *Cacna2d1* is also highly expressed in the prefrontal cortex (Supplementary Fig. 3a), with the pyramidal neurons in the L2/3 having the highest expression levels (Supplementary Fig. 3b). To investigate whether training-induced VGluT1/PSD95-positive synapse formation is dependent on α2δ−1, we bred α2δ−1 heterozygous mice and analyzed the numbers of synapses in the vACC of adult trained and untrained α2δ−1 WT and KO offspring (Fig. 3a).

We found that loss (KO) of α2δ−1 already severely reduces the synapse density in the vACC of the untrained mice (~50% reduction, Fig. 3b, c). Next, we tested if the loss of α2δ−1 affects training-induced synaptogenesis. Here, we compared the numbers of VGluT1/PSD95-positive synapses among untrained and trained α2δ−1 KO mice. The training-induced increase in VGluT1/PSD95-positive synapses in L2/3 was abolished in the α2δ−1 KO mice (Fig. 3d). The average size of the pre-synaptic marker VGlut1 was unaltered (Supplementary Fig. 3c) whereas post-synaptic marker PSD95 puncta size was decreased in α2δ−1 KOs (Supplementary Fig. 3d), a finding in line with the important role of α2δ−1 in spinogenesis^61^. Quantification of VGluT1 and PSD95 puncta numbers did not reveal a clear correlation between the training-induced increase in synapse numbers and changes in the numbers of individual puncta (Supplementary Fig. 3e, f). Our findings show that diminished α2δ−1-signaling reduces excitatory synapse numbers in the vACC of adult mice and prevents training-induced excitatory synaptogenesis in the L2/3 of the vACC. To test if the reduced number of excitatory synapses could have an impact on the overall activity of neurons within the ACC in α2δ−1 KO mice, we quantified the number of c-Fos^+^ cells in untrained and trained α2δ−1 KO mice (Fig. 3e). Compared to WT mice, we found a significant reduction in the number of c-Fos+ cells in the ACC of α2δ−1 KO mice (Fig. 3f). Moreover, there was no difference between the number of c-Fos+ cells in untrained vs. trained α2δ−1 KO mice (Fig. 3f). These results show that in the ACC of α2δ−1 KO mice, there is a reduced number of excitatory synaptic inputs which affects the activity and IEG expression in the ACC neurons. Thus, α2δ−1 KOs can be utilized as a genetic tool to test the role of cortical synaptogenesis on instrumental learning and performance.

### Loss of α2δ−1-signaling does not impair learning of instrumental actions but causes an increase in effort exertion

**Fig. 4:**
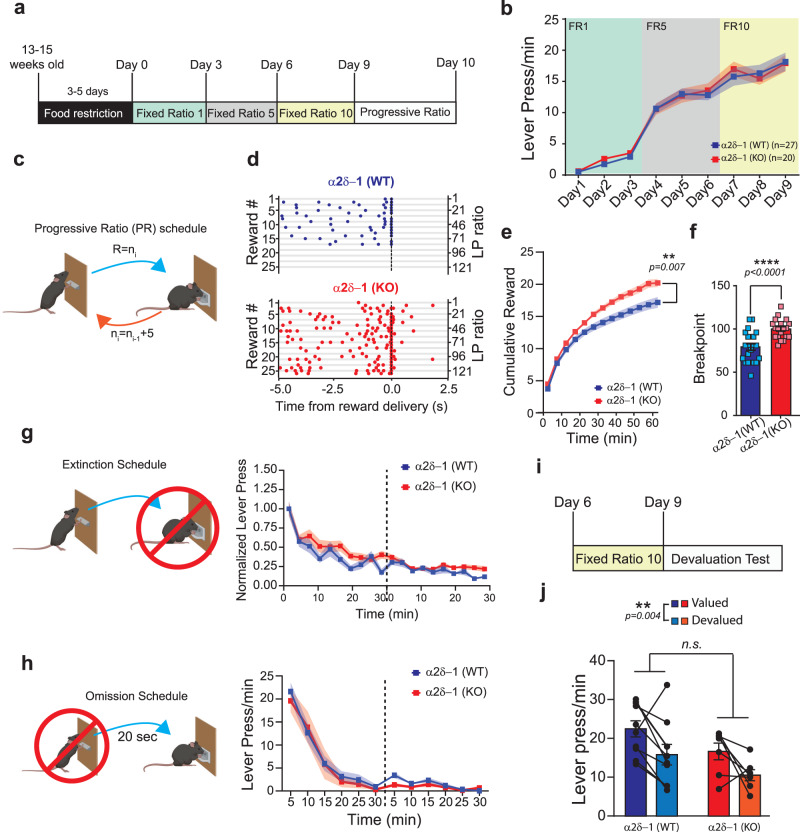
Constitutive lack of α2δ−1 increases effort exertion without affecting the learning of instrumental actions. **a** Schematic representation of the fixed ratio and PR schedule used for the α2δ−1 WT and KO mice. **b** Lever press (LP) rate for the 9 days on the FR schedule for α2δ−1 WT (*n* = 27 mice; 14 male and 13 female) and KO (*n* = 20 mice; 10 male and 10 female). **c** Schematic representation of the progressive ratio (PR) schedule. The value (*n*) of the ratio (R) increment of 5 for every received reward (i), starting with R = 1. **d** Representative peri-reward raster histograms of LP for α2δ−1 WT and KO mice. **e** Cumulative reward count over the PR session (bin=5 min) for α2δ−1 WT (*n* = 22; 17.2 ± 0.8 rewards) and KO (*n* = 19; 20.2 ± 0.6 rewards) animals. **f** Breakpoint for α2δ−1 WT (*n* = 22; Breakpoint = 79.1 ± 3.8 and KO (*n* = 19; Breakpoint = 99.9 ± 2.6). **g** Schematic representation of the extinction schedule in which the action is not reinforced. α2δ−1 WT (*n* = 22) and KO (*n* = 19). The normalized number of lever press is reported for the 2 days of testing (dashed line). **h** Schematic representation of the omission schedule in which the reinforcer is delayed by each press. α2δ−1 WT (*n* = 17) and KO (*n* = 8). The lever press/min are reported for the 2 days of testing (dashed line). **i** Schematic representation of the devaluation test schedule. **j** Lever press/min in valued and devalued states after pre-feeding for α2δ−1 WT and KO mice. Source data are provided as a Source Data file. Drawings (**c**, **g**, **h**) are created with BioRender.com.

We examined whether α2δ−1 KO mice would exhibit deficits in the LP task (Fig. 4a). Surprisingly, there were no genotype or sex differences in the ability of the KO mice to learn and perform the FR lever press schedules (Fig. 4b and Supplementary Fig. 4a, b). Analysis of the lever press patterns established during training confirmed a significant improvement in performance across days in both genotypes (Fig. 4b and Supplementary Fig. 4c–f). These findings suggest that training-dependent, α2δ−1-mediated excitatory synaptogenesis is not necessary to establish instrumental actions.

Then, what is the role of synaptogenesis in the ACC? To answer this question, we next tested the performance of WT and KO mice in a progressive ratio (PR) paradigm. This schedule evaluates the motivational state of animals by adjusting the effort requirement for a reward^87,88^. PR is a standard paradigm to study effort in humans and rodents^37,89–91^, eliminating the confounding effects of choice-based tasks. During the PR test, the number of lever presses required to receive one food reward is progressively increased by an increment of 5 (i.e., 1 LP for the first reward, 6 LPs for the second, 11 LPs for the third, and so on…, Fig. 4c). Mice are expected to stop pressing or reduce their press rates greatly when the number of presses required to achieve the reward becomes too high.

We found that in the PR schedule, as the task became difficult, there was a clear difference between how WT and α2δ−1 KO mice responded to the higher LP demand. Surprisingly, throughout the PR session, the performance of α2δ−1 KO mice was higher than the WT controls (Fig. 4d). As a result, the KO mice received more rewards and reached a significantly higher breakpoint by the end of the PR session (Fig. 4e, f). Lever press bout analysis showed that the bout properties remain unaltered in α2δ−1 KO mice. The difference was found in the Inter-Bout interval (IBI), which was shorter in α2δ−1 KO mice (Supplementary Fig. 4g–j). These results indicate that α2δ−1 KO mice cannot effectively control effort exertion during a demanding task due to more frequently repeated lever press bouts.

However, to reach this conclusion, several other possible interpretations had to be ruled out, such as the establishment of persistent, impulsive, or hyperactive behaviors in the α2δ−1 KO mice. To do so, both α2δ−1 WT and KO mice were tested for their ability to extinguish the LP behavior (Fig. 4g) or to learn the opposite contingency, i.e., not pressing the lever to get the reward (Fig. 4h). In both cases, α2δ−1 WT and KO were able to reduce the number of presses, excluding the possibility of action persistence. We then tested the mice for their hunger level under a food-restricted state. To do so, food-restricted mice were exposed to 3 g of chow or pellet for 30 min on two different days, and the amount of leftover food was then weighted to calculate the amount of consumed food. Both α2δ−1 WT and KO groups consumed similar amounts of chow or pellet, and both genotypes did not show a preference for one of the two types of food (Supplementary Fig. 4k). These results show that the loss of α2δ−1 does not affect hunger level of the mice.

Next, we tested the possibility that in α2δ−1 KOs sensitivity to reward value could have a role in the alteration of the effort/reward relation. To test this possibility, we performed a devaluation test. Mice were trained as usual up to FR10. On the 10th day of testing, trained mice were pre-fed either with standard chow (valued state) or reward pellets (devalued state of reward)^25^. These mice were then tested on a 5-min extinction schedule (Fig. 4i). As expected, α2δ−1 WT mice that were in the valued state pressed more compared to when they were in the devalued state. Comparison of α2δ−1 WT and KO mice in a valued versus devalued reward state showed that α2δ−1 KO mice do not display impaired sensitivity to reward value (Fig. 4j and Supplementary Fig. 4l). These results show that in α2δ−1 KO mice effort control is impaired; however, these KO mice still are able to extinguish the LP behavior and learn to reverse or diminish their behavior when the reward contingencies are altered.

Finally, to determine if α2δ−1 KO mice had a hyperactive phenotype, which may explain enhanced LP behavior, we tested them in an open field to quantify their overall motor activity in a novel environment (Supplementary Fig. 4m). On the contrary to hyperactivity, compared to α2δ−1 WT, the α2δ−1 KOs were significantly less active, as shown by the reduced total distance traveled (Supplementary Fig. 4n). Moreover, α2δ−1 WT mice spent on average 39.8% of their time in the center zone of the open field arena, whereas the α2δ−1 KO mice spent significantly less time in the center (23.7% Supplementary Fig. 4o). These results reveal that loss of α2δ−1 inhibits the training-induced excitatory synapse formation in the mouse ACC, but it does not affect the learning of instrumental actions. Instead, the α2δ−1 KO mice display a profound increase in effort exertion, suggesting that excitatory synaptogenesis in the adult cortex is involved in effort regulation.

### Conditional deletion of α2δ−1 from ACC_→DMS_ neurons is sufficient to increase effort exertion

**Fig. 5:**
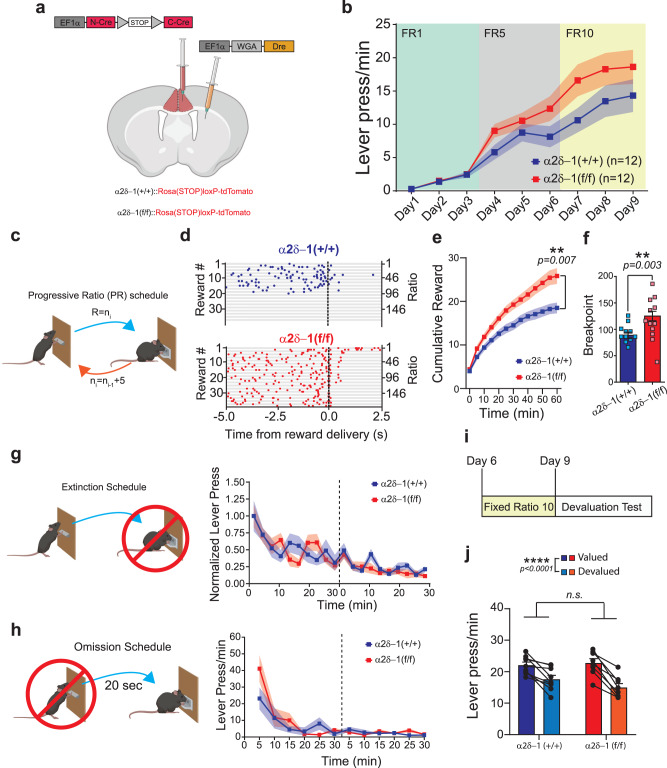
Conditional deletion of α2δ−1 from ACC_→DMS_ neurons increases effort exertion without affecting the learning of instrumental actions. **a** Schematic representation of the injection strategy to conditionally knock out α2δ−1. **b** Lever press rate for the 9 days on the FR schedule for α2δ−1 (+/+) and α2δ−1(f/f) mice. **c** Schematic representation of the progressive ratio (PR) schedule. The value (*n*) of the ratio (R) increment of 5 for every received reward (i), starting with R = 1. **d** Representative peri-reward raster histograms of LP for both groups. **e** Cumulative reward count over the PR session for α2δ−1 (+/+) and α2δ−1(f/f) animals. **f** Breakpoint for α2δ−1(+/+) and α2δ−1(f/f) animals. **g** Left: Schematic representation of the Extinction schedule in which the action is not reinforced. Right: Normalized lever press number in a 3 min bins for α2δ−1 (+/+) and α2δ−1(f/f) animals. **h** Left: Schematic representation of the omission schedule in which the reinforcer is delayed by each press. Right: The lever press/min are reported for the 2 days of testing (dashed line). **i** Schematic representation of the devaluation test schedule. **j** Lever press/min in valued and devalued states after pre-feeding. Source data are provided as a Source Data file. Drawings in (**a**, **c**, **g**, **h**) are created with BioRender.com.

To determine the specific roles of α2δ−1-signaling in the adult ACC_→DMS_ neurons, we utilized α2δ−1(f/f) mice carrying the Cre-reporter Rosa(STOP)loxP-tdTomato (Fig. 5a) and conditionally deleted α2δ−1 selectively from these ACC neurons by utilizing the viral approach described before (Fig. 2f). We trained virally-transduced α2δ−1(+/+) or α2δ−1(f/f) mice using the same behavioral paradigm (Fig. 4a). Similar to α2δ−1 global KOs, we found that circuit-specific deletion of α2δ−1 in adulthood does not affect the learning of the lever press task (FR1 schedule in Fig. 5b). During both FR5 and FR10 schedules, we observed a trending, but not significant, increase in the LP rate for the α2δ−1(f/f) compared to the α2δ−1(+/+) mice (Fig. 5b). Analysis of the lever press bouts (Supplementary Fig. 5a–d) showed an overall improvement of both groups across days. However, a significant difference between α2δ−1(+/+) and α2δ−1(f/f) was observed for the number of presses per bout and bout IPI (Supplementary Fig. 5b, c), showing that mice lacking α2δ−1 in ACC_→DMS_ neurons have an alteration of the lever press sequence, with an increased number of presses within bouts. These results suggest a specific function of α2δ−1 in the ACC_→DMS_ neurons in controlling the properties of the lever press bouts.

Next, we tested the same mice on the PR schedule (Fig. 5c) and observed stark differences between α2δ−1(f/f) and α2δ−1(+/+) in effort exertion. The representative LP raster plots from two PR sessions, one from an α2δ−1(+/+) and the other from an α2δ−1(f/f) mouse, illustrate some of these differences (Fig. 5d). Similar to the α2δ−1 KO mice, α2δ−1(f/f) received a higher number of rewards across the session time (60 min) and reached a significantly higher breakpoint than the α2δ−1(+/+) mice (Fig. 5e, f). Quantitative bout analyses revealed that the change in the performance of the LP sequence (Supplementary Fig. 5e–h) underlies the differences between genotypes. In α2δ−1(f/f) mice, bouts are longer and have a higher number of presses per bout, hence a higher frequency (lower bout IPI), than the α2δ−1(+/+) (#of presses/bout for α2δ−1(+/+) = 12 ± 1.7; and for α2δ−1(f/f) = 25 ± 3.4). These results show that ablating α2δ−1, specifically in ACC_→DMS_ neurons causes a profound alteration of the lever press sequence, resulting in increased effort exertion. On the other hand, the deletion of α2δ−1 from ACC_→DMS_ neurons did not affect the ability of α2δ−1(f/f) mice to extinguish LP behavior when the reward is no longer delivered (Fig. 5g) or to learn the opposite contingency (Fig. 5h).

α2δ−1(f/f) mice were not in a higher hunger state compared to α2δ−1(+//+) mice as they consumed similar amounts of food (Supplementary Fig. 5i). Both genotypes also had similar sensitivity to the reward value of the food (Fig. 5i, j and Supplementary Fig. 5j). These results show that loss of α2δ−1 in the ACC_→DMS_ neurons does not change the hunger state of mice and the mice are still able to respond to reward devaluation by pre-feeding in the same way as the WT.

General hyperactivity was also ruled out, as indicated by the open field test (Supplementary Fig. 5k): there were no differences in total distance traveled (Supplementary Fig. 5l) and time spent in the center of the arena (Supplementary Fig. 5m) between genotypes. Altogether, these data show that loss of α2δ−1 only in the ACC_→DMS_ neurons is sufficient to cause a profound increase in effort exertion when the task progressively becomes more demanding. Furthermore, these results suggest that α2δ−1 is required in the ACC_→DMS_ neurons for new excitatory synapse formation during LP learning to monitor action sequence and regulate effort exertion.

### Conditional deletion of α2δ−1 reduces the number and activity of excitatory synapses in the adult ACC_→DMS_ neurons

**Fig. 6:**
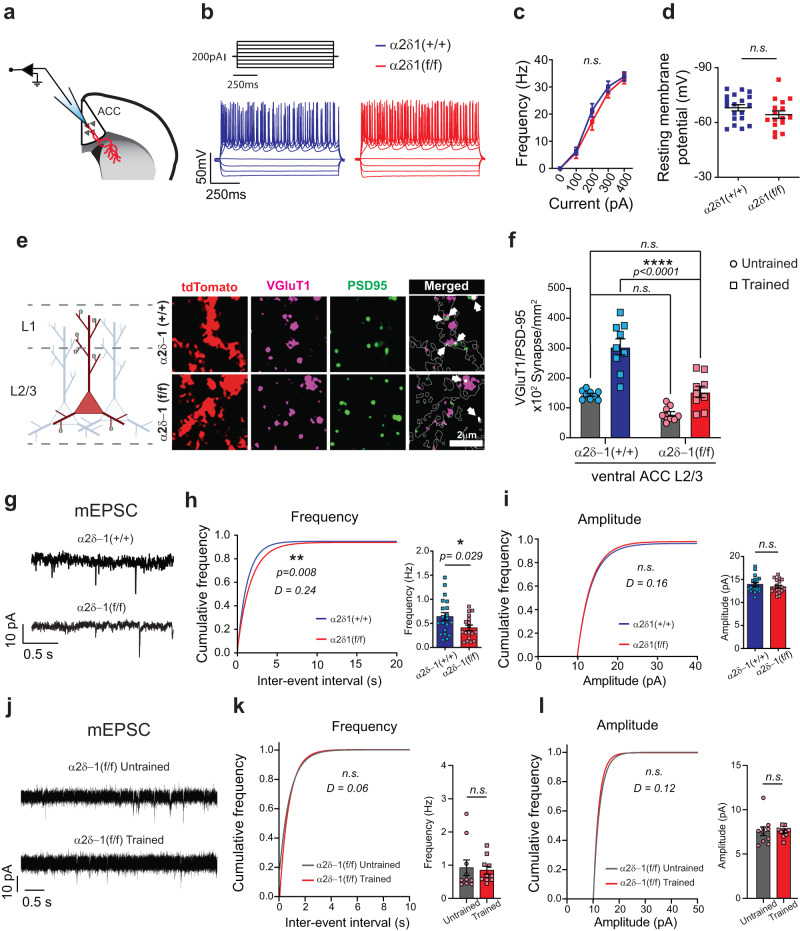
Circuit-specific conditional deletion of α2δ−1 reduces the excitatory synapses number onto the ACC_→DMS_ neurons. **a** Schematic representation of the electrophysiological recordings from ACC_→DMS_ tdTomato^+^ neurons in both α2δ−1(+/+) and α2δ−1(f/f) mice. **b** Example traces of the intrinsic excitability. **c** Action potential (AP) frequency as function of the injected current. **d** Average resting membrane potential. **e** Left: Schematic representation of the tdTomato^+^ neurons used to quantify the VGluT1-PSD95 synapses (created with BioRender.com). Right: Representative images. White arrows point at the puncta within the tdTomato mask. **f** Quantification of synaptic density between conditions. **g** Example traces from mEPSC recordings. **h** Left: Cumulative distribution of the Inter-event interval. Right: Average frequency of mEPSCs. **i** Left: Cumulative distribution of amplitude in pA. Right: Average amplitude. **j** Representative traces from mEPSC recordings from untrained and trained α2δ−1(f/f) mice. **k** Left: Cumulative distribution of the Inter-event interval. Right: Average frequency. **l** Left: Cumulative distribution of amplitude. Right: Average amplitude. Source data are provided as a Source Data file.

Why does the loss of α2δ−1 in the ACC_→DMS_ neurons cause an increase in effort exertion? Previously, dorsal-root ganglia neurons lacking α2δ−1 were shown to have reduced action potential (AP) firing frequency^92^, so we wondered if changes in the AP firing frequency of ACC_→DMS_ neurons underlie this behavioral phenotype. To test this possibility, we performed current step stimulation of the Cre^+^ (tdTomato^+^) ACC_→DMS_ neurons of α2δ−1(+/+) and α2δ−1(f/f) mice (Fig. 6a, b) during whole-cell patch-clamp recordings. We observed no differences between the mean AP firing frequencies of the tdTomato^+^ neurons between the genotypes in any of the step current stimulations used to elicit neuronal APs (Fig. 6b, c). Moreover, we did not observe a difference in the mean resting membrane potentials between α2δ−1(+/+) and α2δ−1(f/f) neurons (Fig. 6d), showing that loss of α2δ−1 does not alter the excitability of the ACC_→DMS_ neurons.

In the visual cortices of constitutive α2δ−1 KO mice, L2/3 neurons displayed a severe reduction in the frequency of mEPSCs compared to littermate WTs^61^, so we next tested whether loss of α2δ−1 in ACC_→DMS_ neurons would lead to a reduction in the density of synaptic inputs made onto these cells. To do so, first, we quantified the number of VGluT1/PSD95-positive synapses made onto the tdTomato^+^ neuronal processes by masking the TdTomato channel and creating ROIs for the specific quantification of the excitatory inputs onto ACC_→DMS_ neurons (Fig. 6e). These analyses were made both in L1 and L2/3 because the L2/3 ACC_→DMS_ pyramidal neurons extend their apical dendrites to L1 and basal dendrites to L2/3 (Fig. 6e). Training induced a significant increase in the density of VGluT1/PSD95-positive synapses made onto the α2δ−1( + / + ) ACC_→DMS_ neuron processes in L1 (Supplementary Fig. 6a). Deletion of α2δ−1 from the ACC_→DMS_ neurons abolished the training-induced increase in synapse density in L1 (Supplementary Fig. 6b). However, in L2/3, training still induced a significant increase in the density of synapses in both genotypes (Supplementary Fig. 6a, b). However, the synapse density of trained α2δ−1(f/f) ACC_→DMS_ neurons was significantly lower than the trained α2δ−1(+/+) and not different from the untrained α2δ−1(+/+) (Fig. 6f). Quantification of VGluT1 and PSD95 individually did not reveal a clear correlation with the increase in co-localized puncta (Supplementary Fig. 6c, d). Finally, we recorded mEPSCs from the ACC_→DMS_ neurons of trained α2δ−1(f/f) mice and trained α2δ−1(+/+), and we found a significant reduction in the frequency but not the amplitude of the mEPSCs (Fig. 6g–i).

As opposed to the training-induced structural and functional increases in excitatory synapses in WT mice (Fig. 2e, h–j), in the α2δ−1(f/f) ACC_→DMS_ neurons, we found that the observed structural increase is not sufficient to drive a functional change. Recordings of mEPSCs in the α2δ−1(f/f) ACC_→DMS_ neurons from L2/3 neurons of trained and untrained mice revealed no differences in the frequency or amplitude of the mEPSCs after training (Fig. 6j–l).

Taken together, these electrophysiological and neuroanatomical analyses show that circuit-specific conditional deletion of α2δ−1 in the adult ACC_→DMS_ neurons reduces the number and function of excitatory synaptic inputs made onto these neurons without changing intrinsic excitability. These results suggest that training-induced excitatory synaptogenesis is required to excite the ACC _→ DMS_ neurons properly. In turn, the activity of ACC_→DMS_ neurons controls effort exertion by suppressing lever press behavior when the task becomes too demanding.

### Optogenetic excitation of ACC_→DMS_ neurons is sufficient to reduce effort exertion in WT and α2δ−1 circuit-specific knockout mice

**Fig. 7:**
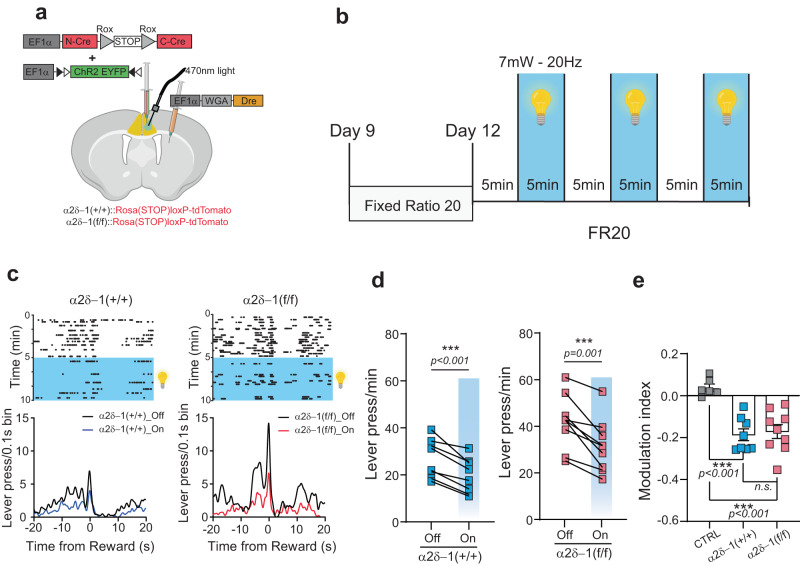
Optogenetic excitation of ACC_→DMS_ neurons inhibits the lever press behavior. **a** Schematic representation of the viral injections and fiber implants for the optogenetic rescue experiments in the ACC_→DMS_ projecting neurons of α2δ−1(+/+) and α2δ−1(f/f) mice. **b** Schematic representation of the FR20 schedule used for the optogenetic experiments with 5 min Off and On schedule. **c** Representative peri-reward raster histograms of an α2δ−1(+/+) and an α2δ−1(f/f) mouse during the light-Off and light-On periods. **d** Lever press/min for the α2δ−1(+/+) mice (*n* = 8) and α2δ−1(f/f) mice (*n* = 11). Paired two-tailed *t* test for α2δ−1(+/+) light-Off (27 ± 2.9) and light-On (20 ± 2.6) [*t* (7) = 6.9; *P* = 0.0006] and for α2δ−1(f/f) light-Off (42 ± 3.8) and light-On (32 ± 3.8) [*t* (10) = 6.7]. **e** Modulation index for CTRL, α2δ−1 (+/+), and α2δ−1 (f/f) mice. One-way ANOVA [*F* (2, 20) = 16.27, *P* < 0.001], Tukey’s multiple comparisons revealed a significant difference between CTRL and α2δ−1 (+/+) [q (20) = 7.3; *P* = 0.0001] and between CTRL and α2δ−1 (f/f) [*q* (20) = 7.0; *P* = 0.0002] and no differences between α2δ−1 (+/+) and α2δ−1 (f/f) [*q* (20) = 0.56, *P* = 0.916; *P* = 0.916]. For all graphs: data showed as mean ± s.e.m. alpha = 0.05. Source data are provided as a Source Data file. Drawings in (**a**–**c**) are created with BioRender.com.

To determine if the excitation of ACC_→DMS_ neurons is sufficient to reduce effort exertion during the LP behavior, we expressed a Cre-dependent light-gated cation-selective membrane channel, Channelrhodopsin-2 (flex-ChR2)^93^, in these neurons. To do so, α2δ−1(+/+) and α2δ−1(f/f) mice received bi-lateral injections of the WGA-Dre virus in the DMS and a 1:1 cocktail of N-Cre-*rox-STOP-rox*-C-Cre and flex-ChR2 viruses in the ACC (Fig. 7a). Optic fibers were also implanted bilaterally in the ACC to enable activation of ACC_→DMS_ neurons (Fig. 7a). To compare the LP behavior of the same mouse with or without optogenetic stimulation of ACC_→DMS_ neurons, we tested the mice with a more demanding fixed ratio schedule, FR20. For these optogenetic experiments, we cannot use the PR schedule because the LP/reward ratio changes over time. Thus, during the PR schedule, we cannot compare the lever press rate of the same mouse with or without stimulation within the same session.

Mice were trained for 9 days as described previously. Subsequently, they were trained for an additional two days on an FR20 schedule (Fig. 7b). On day 12, we tested these mice over a 30 min session, during which we alternated 5 min of optogenetic stimulation (light-On) with 5 min of no stimulation (light-Off) (Fig. 7b). In a control group of mice (CTRL), which had only fiber implants into the ACC but no virus, we controlled for possible effects of the light exposure and surgery on behavioral performance (Supplementary Fig. 7a)^94^. We observed that these CTRL mice behave similarly during periods of light-On and light-Off, showing that light exposure in the absence of ChR2 expression had no or little effect on their behavior (Supplementary Fig. 7b, c). For each mouse, we confirmed the anatomical locations of the optic fiber placement and the efficiency of viral targeting and co-expression of Cre (tdTomato^+^) and ChR2 (EYFP^+^) in the ACC_→DMS_ neurons (Supplementary Fig. 7d, e). The colocalization of the tdTomato (Cre) and EYFP (ChR2) signals was observed in more than half of the tdTomato^+^ cells (Supplementary Fig. 7f, g), with L2/3 having the highest percentage of positive cells among all the layers (Supplementary Fig. 7h).

As shown by the representative raster plots and histograms (Fig. 7c), in the absence of optogenetic stimulation, the numbers of LPs are increased in α2δ−1(f/f) compared to α2δ−1(+/+) mice (Fig. 7c, d). However, during the light-On intervals, both α2δ−1(+/+) and α2δ−1(f/f) mice reduced their LP rate (Fig. 7c, d). To quantify this change, we calculated the modulation index denoting the difference in the number of LPs per 15 min light-On compared to the 15 min of the light-Off periods divided by the total number of LPs for the entire 30-min session (Fig. 7e). The mice expressing ChR2 in the ACC_→DMS_ neurons displayed a 20% reduction in the number of LPs when stimulated (Fig. 7e). Importantly, planned *t* test analysis showed that under light-On conditions, the number of LP of the α2δ−1(f/f) mice were comparable with those of the α2δ−1(+/+) mice during light-Off periods (Fig. 7d, planned unpaired two-tails *t* test [*t* (15) = 0.98], *P* = 0.344). These results indicate that when α2δ−1 is deleted in ACC_→DMS_ neurons, LP behavior can be rescued by optogenetic activation of these neurons. Taken together, we found that optogenetic excitation of ACC_→DMS_ neurons is sufficient to reduce the effort exerted during a demanding task. Furthermore, when considered together with our anatomical and functional analysis (Fig. 6), these data strongly indicate that α2δ−1-mediated synaptogenesis is required for the ACC_→DMS_ neuron activation to regulate effort exertion and that reduced activity of the ACC_→DMS_ neurons increase the amount of effort exerted during task performance.

### Optogenetic inhibition or excitation of ACC_→DMS_ neurons inversely modulates the effort exerted during a demanding task

**Fig. 8:**
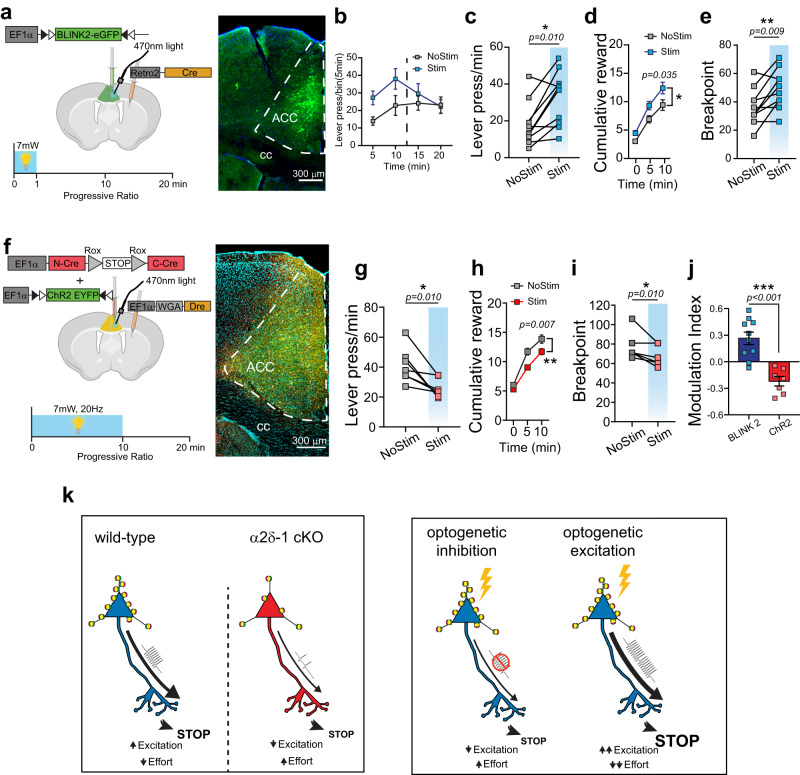
Inhibition and excitation of ACC_→DMS_ neurons have opposite effects on the performance of lever press bout and effort exertion. **a** Left: Schematic representation of viral injections for the BLINK2 and behavioral schedule used for inhibitory experiments and Right: Example image of BLINK2 expression within ACC_→DMS_ neurons. **b** Lever press/min of the entire PR test for BLINK2 mice showing the effect of BLINK2 inhibition for the first 10 min upon stimulation. *n* = 10 mice. **c** Quantification of behavioral parameters during the first 10 min of PR for no stimulation (NoStim) and stimulation (Stim) conditions. Lever press/min during NoStim (18 ± 3.8) and Stim (30 ± 4.5) days of PR. **d**, Cumulative reward count during NoStim and Stim days of PR. **e** Breakpoint between NoStim (37 ± 4.0) and Stim (49 ± 4.4) days. **f** Left: Injection strategy for ChR2 expression and the protocol used for optogenetic experiments using ChR2 during PR schedule. Right: Example image of ChR2 expression in ACC_→DMS_ neurons. **g** Quantification of behavioral parameters during the first 10 min of PR for NoStim and Stim conditions. Lever press/min during NoStim (41 ± 4.4) and Stim (26 ± 2.4) days of PR. **h** Cumulative reward count during NoStim and Stim days of PR. **i** Breakpoint between NoStim (77 ± 5.2) and Stim (67 ± 3.8) days. **j** Modulation index of BLINK2 (0.26 ± 0.07; *n* = 10) and ChR2 (−0.22 ± 0.05; *n* = 7) mice during the PR schedule. **k** Left: Proposed intrinsic mechanism of regulation of effort exertion through training-induced excitatory synaptogenesis. Right: Representation of how optogenetic manipulation of ACC_→DMS_ neurons would affect the effort exertion. Source data are provided as a Source Data file. Drawings in (**a**, **f**, **k**) are created with BioRender.com.

To test if inhibition of ACC_→DMS_ neurons could phenocopy the α2δ−1(f/f) mice and cause an increase in effort exertion, we expressed an inhibitory step-function opsin called BLINK2 in ACC_→DMS_ neurons (Fig. 8a). BLINK2 activation by light provides sustained neuronal inhibition after a short period of optogenetic stimulation^95^. For these experiments, mice were tested on a PR schedule with and without optogenetic stimulation. We found that 1 min of light delivery at the beginning of a PR schedule causes an increase in the number of lever presses (Fig. 8b). After 10 min, the inhibitory effects of the optogenetic manipulation subside, and the LP rate returns to the levels observed without stimulation (Fig. 8b). We statistically analyzed the effect of BLINK2-mediated inhibition during the first 10 min after stimulation onset. These data show that inhibition of the ACC_→DMS_ neurons is sufficient to increase LP rate, the number of rewards obtained, and the breakpoint reached when compared to the performance of the same mice on a day in which they did not receive optogenetic stimulation (Fig. 8c–e). Analysis of the lever press bouts revealed that inhibition of ACC_→DMS_ neurons also increases the frequency and number of lever presses per bout, as observed in α2δ−1(f/f) mice (Supplementary Fig. 8a–d).

Next, we performed optogenetic excitation of ACC_→DMS_ neurons under similar conditions in mice co-expressing Cre (tdTomato^+^) and flex-ChR2 (EYFP^+^) in the ACC_→DMS_ neurons (Fig. 8f). In contrast to the inhibition of ACC_→DMS_ neurons, light stimulation during the first 10 min of a PR schedule caused a significant reduction in the number of LP, cumulative rewards and breakpoint (Fig. 8g–i). Indeed, calculation of the modulation index for inhibition and excitation of ACC_→DMS_ neurons shows opposite effects of optogenetic manipulation on mouse behavior (Fig. 8j). Analysis of the lever press bouts revealed that the reduced effort exertion was due to the performance of shorter bouts with lower number of presses (Supplementary Fig. 8e–h).

These results reveal that the activity of ACC_→DMS_ neurons bidirectionally regulates the amount of effort exerted during a demanding task by modulating the lever press bout properties. These data also suggest a role for the ACC_→DMS_ neurons in monitoring and controlling the performance of goal-directed action sequence.

## Discussion

Synaptogenesis is critical for the construction of brain circuits during development^5,7,64,96,97^. However, little is known about the role of synaptogenesis in adult brains. We show that new excitatory synapses are formed onto the ACC_→DMS_ neurons during instrumental operant conditioning training. These new synapses are not necessary for learning the action-outcome relationship but are required for adaptively adjusting the effort. The Thrombospondin-Gabapentin receptor α2δ−1 mediates training-induced excitatory synapse formation onto the ACC_→DMS_ neurons. When α2δ−1 is lost, training-induced excitatory synaptogenesis is diminished, generating a behavioral phenotype in which mice spend significantly more effort to achieve the same reward. Concordantly, circuit-specific excitation of ACC_→DMS_ neurons is sufficient to reduce the effort exerted during instrumental actions, whereas their inhibition increases the effort exerted. Taken together, our findings offer a circuit-specific cellular and molecular mechanism for the cognitive control of effort (Fig. 8k).

In our behavioral paradigm, the number of required lever presses represents the cost, whereas the food pellet reward represents the benefit. When the task becomes too demanding (e.g., in PR or FR20 schedules), WT animals reduce the effort because the cost is too high. Our findings show that loss of α2δ−1-signalling disrupts this adaptive step. We used the PR test to measure effort exertion, because it avoids the confound of having to select between different options, such as choosing between two actions with different probability of reward or two different locations with different rewards^20,38,40,70,98,99^. In fact, PR test is a standard test to measure effort exertion both in rodents and humans^37,89–91^.

To interpret the behavioral phenotype in α2δ−1 global and circuit-specific KOs as increased effort exertion, we ruled out other possibilities. A possible alternative explanation for increased lever press behavior in α2δ−1 KOs could be the establishment of persistent or habitual behaviors. If that were the case, we would expect persistent lever pressing during the extinction test or no effect on lever pressing after outcome devaluation. But instead, the KO mice were able to extinguish the behavior and adjust their effort after reward devaluation (Figs. 4 and 5 and Supplementary Figs. 4 and 5). Moreover, mice with α2δ−1 deletions did not show impaired reversal learning, further showing that learning the action-outcome relationship is intact in α2δ−1 KOs (Figs. 4 and 5). Finally, general hyperactivity was ruled out using an open-field test to evaluate the overall movement in a novel environment (Supplementary Figs. 4 and 5). Moreover, optogenetic excitation or inhibition of only the ACC_→DMS_ neurons is sufficient to modulate lever press rates within the same mice, showing that this particular circuit is a regulator of effort exertion (Figs. 7 and 8).

Loss of α2δ−1 or optogenetic modulation of ACC_→DMS_ neurons caused increased effort exertion by affecting the action sequence (bout) properties. As the effort exertion increased, the number of lever presses per bout increased, and the inter-press interval decreased. These changes occurred without an increase in bout duration or inter-bout intervals (Supplementary Figs. 5 and 8). These results show that the ACC_→DMS_ neurons control effort exertion by modifying the rate of lever press within a bout.

In mice and non-human primates, ACC activity is proposed to be necessary during behavioral tasks in which action-outcome contingencies guide future behavioral decisions^100–102^. For example, in a foraging simulation, monkeys with ACC lesions were more likely to repeat a learned action even if the probability of receiving the reward decreased^101^, suggesting that the ACC is involved in evaluating the changes in the relationship between the effort and reward. In rats, a role for ACC in effort evaluation was first identified using a cost-benefit T-maze task after an excitotoxic lesion of the ACC^55,56^. ACC-lesioned rats were less likely to choose the high-cost/high-reward option compared to sham animals, suggesting that ACC activity promotes effortful choices. However, other studies showed that ACC lesion does not have an effect on motivation^72^ and ACC activity during effort-related tasks could vary based on task complexity and the existence of alternative options^57,103,104^. All these studies were conducted by manipulating or ablating all the neurons within the ACC. In our study, we pinpointed a specific subpopulation of ACC neurons that project to the DMS in the regulation of effort control. Future studies investigating the molecular and functional landscape of ACC neurons and further delineating their distinct outputs are likely to yield new insights into the role of ACC in the control of goal-directed behaviors.

From a molecular and cellular standpoint, it is intriguing that the ACC is thought to guide future behaviors based on recent experience. Indeed, the time scale of synaptogenesis (days) is unlikely to underlie the online adjustment of performance by ACC. Instead, our findings suggest that new synapses are formed onto the ACC_→DMS_ neurons during training as an anticipatory mechanism for future scenarios in which the effort needed for the learned behavior increases. New findings suggest that training-induced modification of synaptic connectivity could be a common mechanism used by other PFC regions to guide an adaptation of behavioral response. For example, learning induces the formation of new spines in the Orbitofrontal cortex (OFC) neurons projecting to the DMS. These newly formed spines are necessary to retrieve previous memories of learned actions and select actions with higher reward^20^. It is possible that OFC and ACC connections to the DMS have synergistic functions to regulate goal-directed learned behaviors.

Previously, we found that the Thrombospondin-Gabapentin receptor, α2δ−1, is required for the formation of intracortical excitatory synapses in the developing visual cortex^61^. Loss of α2δ−1 causes more than 50% reduction in excitatory synapse numbers, function, and dendritic spines. Here, we found that α2δ−1 is required for proper intracortical connectivity in the adult ACC as well. The lack of α2δ−1 is sufficient to abolish the training-induced increase in excitatory synapse numbers and activity in this brain region (Figs. 2, 3, and 6).

New synapse formation in the motor cortex was previously proposed as an important step in motor learning and memory^16,105^. Consequently, at first, we expected the α2δ−1 mutants to have deficits in initial instrumental learning. A previous study using a different α2δ−1 knockout line and behavioral paradigms found that lack of α2δ−1 impaired motor and spatial learning^106^. Surprisingly, we found no learning impairments in α2δ−1 global or circuit-specific KO mice in instrumental training. Instead, our findings show that α2δ−1-signaling in adults controls effort exertion by regulating the formation of excitatory synapses onto the ACC_→DMS_ neurons.

α2δ−1 is a neuronal receptor for Thrombospondins (TSPs), astrocyte-secreted synaptogenic proteins. TSP-α2δ−1 signaling stimulates the formation of silent, structural synapses containing only NMDA receptors, whereby astrocytes can activate them by recruiting AMPA receptors to the synapse^107^. Our findings imply that astrocyte-to-neuron signaling might be upstream of training-induced synaptogenesis. Future studies investigating the roles of astrocytes and the TSPs that they secrete in the control of goal-directed learned behaviors might be fruitful.

The importance of synaptogenesis in brain development is well-established, but whether synaptogenic signaling performs critical functions in the adult brain to regulate complex, learned behaviors is unknown. Mutations in genes controlling synapse formation and maturation are strongly linked to many neuropsychiatric disorders, including ASD, Schizophrenia, OCD, and Alzheimer’s Disease^42,43,108–110^. A common hallmark of these disorders is the presence of repetitive and ineffective behaviors^111,112^. Our findings reveal a link between synaptogenic signaling in the adult brain and the control of the adaptability of learned behaviors, suggesting that dysfunctional adult synaptogenesis may underlie the etiology of behavioral inflexibility seen in such disorders.

## Methods

### Animals, housing, and genotyping

All mice were used in accordance with the Institutional Animal Care and Use Committee (IACUC) and the Duke Division of Laboratory Animal Resources (DLAR) oversight (IACUC Protocol Numbers A173−14-07, A147−17-06, and A263−16−12). All mice were housed under typical day/night conditions of 12-h cycles with an ambient temperature of 22 degrees Celsius and humidity at 50%. Wild-type *C57BL/6J* (Stock #000664) and ROSA(STOP)loxP-td-Tomato (B6;129S6-Gt(ROSA)26Sor^tm14(CAG-tdTomato)^Hze/J; Stock #007914) lines were obtained through Jackson. The constitutive α2δ−1 Het and KO mice, as well as the conditional α2δ−1(f/f) mouse lines, were generated from our laboratory^61^. All male and female mice (3-5 months old) used in this study were handled for 5−10 min a day for a week to get used to the experimenter. After this time, animals were food-restricted for 3–5 days until they reached 85–90% of their normal body weight. The target weight was maintained stable by daily feeding them, with 1.5/2 g of home chow, after training.

### Instrumental training

Lever pressing training was performed in operant chambers (St Albans, VT, USA) set within light resistant and sound attenuating walls. Each chamber was equipped with a food magazine, where an infrared beam recorded the head entries into the magazine, and pellets were received from a dispenser. The delivered reinforcer was a Bio-Serv 14 mg Dustless Precision Pellets (Bio-Serv, NJ, USA). Each chamber also had two retractable levers on either side of the magazine and a 3-W 24-V house light mounted to the opposite side of the levers. A computer with the Med-PC-IV program was able to control the equipment and record the behavior according to the desired schedule. Timestamps for each lever press and head entries were recorded with a 10 ms resolution and then analyzed using custom-written programs (available upon request). Lever pressing training was performed using only the left lever.

#### Fixed ratio schedule

To test the capability of learning new behaviors, we used a continuous reinforcement schedule, here named fixed ratio 1 (FR1). The first day of FR1 began with three food pellets left in the food magazine, allowing the mice to learn about the possibility of receiving food and the location of food delivery. Then, the lever was inserted into the chamber, and the house light was illuminated. The initial schedule consisted of 3 days of FR1, during which the animals received a pellet for each lever press. The session ended when one of the two restrictions was reached, 120 min or 50 reinforcers (food pellets), with the retraction of the lever and the light turned off. At the end of the 3 days of FR1, mice were moved to three different testing schedules. Three days of FR5 schedule, during which the animals received a pellet for every 5 lever presses, were followed by the other three days of FR10, increasing the number of lever presses up to 10 for one reinforcer. The session ended when one of the two restrictions was reached, 60 min or 50 reinforcers, with the retraction of the lever and the light turned off. The lever press bout analysis was performed using Matlab. The average inter-press interval probability distribution was used to define the bout start and bout end events. In all the cases in which multiple conditions were compared, the control condition was used to define bout start and bout end events for both groups to identify changes from the control condition. At the end of these 9 days, the mice were either sacrificed within 1 h for histological analysis and RNA purification or shifted to the next step of the behavioral test.

#### Progressive ratio, extinction, omission and revaluation tests

A progressive ratio (PR) schedule was used to evaluate the motivation of the mice. The PR schedule consists of an increasing number of lever presses every time a reinforcer is received. In our case, we decided to use 5 as increased progression. The session ended after 60 min with the retraction of the lever and the light turned off. Extinction schedule occurred across two consecutive days during which mice could press the lever, but there was no food delivered as a reinforcer. The session ended after 30 min with the retraction of the lever and the light turned off. The omission schedule occurred across two consecutive days, during which the reinforcer was delivered at a fixed interval of 20 s unless a lever press happened. At every press performed by the mouse, the timer for the reinforcer delivery reset, resulting in a delay in the reinforcer delivery. Outcome devaluation was performed after training the mice up to the FR10 schedule. On day one, mice were exposed to 0.5 g of home cage food for 30 min (valued state). After retraining for FR10, mice were then exposed to 0.5 g of reinforcer pellets for 30 min (devalued state). After each pre-feeding session, a 5-min extinction test was given. The test began with the illumination of the house light and insertion of the left lever and ended with the retraction of the lever and the offset of the house light. The number of presses on each lever was recorded.

### Hunger test

Food-restricted mice were habituated for 3–4 days to be singly housed for 30 min in a new cage. Then, on 2 consecutive days, 3 g of the home chow or the pellets were placed in the cage to be used as reinforcement. Mice were given 30 min of free access to the food in the cage, and leftover food was weighed to calculate the amount consumed. Mice were then regrouped in their home cage.

### Open field test

After the operant task under food deprivation was completed, mice were normally housed with food and water provided ad libitum for 2–3 days, until their normal body weight was reestablished. Mice were then tested in an open field chamber equipped with a blackfly camera (Flyr System, BFS-U3-04S2M-CS) to record the animal movement. The Bonsai software (https://bonsai-rx.org/) was used to automatically detect and record the *x* and *y* coordinates for the center of mass of the mouse. The session began after the mice were acclimated for 30 min to the new room. The mouse was positioned to the center of the arena at the start of the recording, which then finished after 30 min. The chamber was carefully cleaned, and the bedding was changed between different groups of littermates to avoid distraction due to other animals‘ odor stimuli.

### c-Fos staining and analysis

Both trained WT mice (*n* = 6) and untrained WT mice (*n* = 6) trained α2δ−1 KO (*n* = 4) and untrained α2δ−1 KO (*n* = 4) were anesthetized with 200 mg/kg tribromoethanol (avertin) and then terminated by perfusing with a solution made of TBS with heparin (0.1128 g Heparin ammonium salt from porcine intestinal mucosa [Sigma; H6279]) and then with 4% Paraformaldehyde (PFA) within 1 h after the last day of FR10 schedule to observe changes in the activity-dependent expression of the immediate-early gene c-Fos. Mouse brains were then kept in 4% PFA o.n. at 4 °C. The day after, brains were rinsed 3 times with TBS, immersed in 30% Sucrose in TBS, and stored at 4 °C until they were not floating anymore. At this time, brains were included in a mixture of 30% Sucrose in TBS and Tissue Tek O.C.T. compound (frozen tissue matrix) at a 2:1 ratio and stored at −80 °C. Using a cryostat, the brains were cut into 25–30-μm coronal sections and stored in 50% Glycerol in TBS in a 24 multi-well plate, five sections per well. WT sections were chosen to have a 100 μm spatial representation in the rostro-caudal direction. Four sections for each WT were selected at these approximate ( ± 0.2) coordinates relative to Bregma: +2.2, +1.7, +0.4 and −0.3. Sections to make for a total of six ACC hemispheres analyzed per brain were selected for the a2d1-KO condition. The sections were rinsed three times (1 × 2 min, 1 × 20 min, and 1 × 30 min) in TBS, then they were moved for 1 h in a blocking solution containing 10% Normal Donkey Serum (NDS) in TBS + 0.3% v/v of Triton. Primary antibody, Rabbit anti-cFos (Calbiochem, PC05), was diluted 1:50 in the blocking solution with 5% NDS + 0.01% v/v of Sodium azide. The sections were incubated in this solution for 48–72 h at 4 °C with gentle shaking. After this time, they were transferred to a secondary antibody solution after three washing steps in TBST (Triton 0.2%) (1 × 10 min, 1 × 30 min, 1 × 40 min). Donkey anti-rabbit conjugated to Alexa fluor-594 (1:500) was used as the secondary Ab in 10% NDS blocking solution + 0.3% v/v Triton to incubate the sections 2 h at RT. DAPI (1:50,000) was added 15 min before the end of this step, and then sections were rinsed again three times in TBST (Triton 0.2%) before mounting them on Superfrost Plus slides using a Refractive Index solution (RI solution: 20 mM Tris (pH 8.0), 0.5% N-propylgallate, 90% glycerol).

Tile scan images were then acquired using an Olympus Fluoview confocal microscope using ×20 lens in resonant scanner mode, allowing the fast acquisition of entire coronal sections with an optical section step of 1 μm and the necessary number of images/stacks to acquire the entire depth of the section. Images were then processed using a combination of software. All the sections’ stitching was performed either with a custom code (https://github.com/ErogluLab/CellCounts) or through the Olympus microscope software and finally, segmentation for DAPI^+^ and c-Fos^+^ (from now called c-Fos^+^) cells was performed using the UNet software (https://arxiv.org/abs/1505.04597, adapted by Chaichontat (Richard) Sriworarat). In this case, images that were manually segmented by the user to indicate the DAPI^+^ and c-Fos^+^ cells, as well as ROIs that were not positive for either marker, were used to train the UNet neural network. The training process was reiterated until the segmentation was able to recognize the positive cells correctly on a small set of data and then applied to analyze the entire batch of images. The segmentation step produced 16-bit images with the mask of the segmented c-Fos^+^ cells in which the intensity is indicative of the degree of confidence of the segmentation. The mask was then used to select, based on the intensity, and count the c-Fos^+^ cells using the WholeBrain software^69^. For the α2δ−1 KO image analysis focusing on the ACC only, images were first cropped using Fiji then segmented. All the brain regions represented by less than three mice were not considered in the analysis.

### RNA sequencing preparation and analysis

RNA-sequencing libraries were made from ≥500 ng of purified mouse ACC (*n* = 6 mice per condition; *n* = 3 mice per sex; 1 untrained male mouse was removed from the final analysis for having a low number of reads) RNA using the Kapa Stranded mRNA-seq kit. For each replicate 40–72 million, 2 × 51 reads were obtained from a NovaSeq 6000. Raw reads were adapter trimmed using Trimmomatic^113^ (v0.38), aligned to the reference mouse genome (mm10; GRCm38) using Bowtie2^114^ (v2.3.5.1), and counted using Subread^115^ (featureCounts^116^, v1.6.3). Differential gene expression was conducted using edgeR^117^ (v3.30.3). Gene-Ontology (GO terms) and KEGG pathway analyses were done with R using the clusterProfile (v4.6.2)^118^ package.

### Synaptic staining

Brain sections were washed three times and then permeabilized in TBS with 0.2% Triton-X 100 (TBST; Roche, Switzerland) at room temperature. Sections were blocked in 5% Normal Goat Serum (NGS) in TBST for 1 h at room temperature. Primary antibodies (guinea pig anti-VGlut1 1:2000 [AB5905, Millipore, MA], rabbit anti-PSD95 1:300 [51–6900, Invitrogen, CA], guinea pig anti-VGAT 1:1000 [Synaptic Systems 131 004], rabbit anti-Gephyrin 1:500 [Synaptic Systems 147 002], were diluted in 5% NGS containing TBST. Sections were incubated overnight at 4 °C with primary antibodies. Secondary Alexa-fluorophore (488, 594, and 647) conjugated antibodies (Invitrogen) were added (1:200 in TBST with 5% NGS) for 2 h at room temperature. Slides were mounted in Vectashield with DAPI (Vector Laboratories, CA) and images were acquired on an Olympus Fluoview confocal microscopy using a ×60 oil lens at ×1.64 Zoom.

### Quantification of synapses

In all, 3–4 animals per genotype of WT, α2δ−1 KO, α2δ−1(f/f), and α2δ−1(+/+) were used for synapse analysis. Three independent brain sections per group were used for immunohistochemistry. In all, 5 µm thick confocal z-stacks (optical section depth 0.33 µm, 15 sections/z-stack) of the ACC (layer 1,2/3,5), DMS were imaged at ×60 magnification on an Olympus Fluoview confocal laser-scanning microscope. Maximum projections of three consecutive optical sections (corresponding to 1 µm total depth) were generated from the original z-stack. Analyses were performed blindly as to genotype. The Puncta Analyzer plugin (written by Barry Wark, modified by Chaichontat (Richard) Sriworarat, and available upon request from Cagla Eroglu at c.eroglu@cellbio.duke.edu for either ImageJ (NIH; http://imagej.nih.gov/ij/) or FIJI (https://imagej.net/Fiji/Download) was used to count the number of co-localized puncta. This quantification method is based on the fact that pre- and post-synaptic proteins (such as VGluT1 and PSD95) are not within the same cellular compartments of neurons (axons versus dendrites, respectively) and would only appear partially co-localized at synaptic junctions due to their proximity. For the analysis of the synapses made specifically onto ACC_→DMS_-neurons, we only counted the co-localized puncta within the ROI that was created by masking the TdTomato signal marking the neuronal processes of the Cre-expressing neurons. This allowed for the quantification of the VGluT1/PSD95-positive excitatory inputs made specifically onto the ACC_→DMS_-neurons.

The quantification of synapses as the close apposition of pre and postsynaptic markers yields an accurate estimation of the number of synapses both in vitro and in vivo because single pre- or postsynaptic protein signals are also punctate, but they often accumulate in extrasynaptic regions during the course of their life cycle. In agreement, numerous previous studies by ourselves and others have shown that synaptic changes observed by this quantification method are verified by techniques such as electron microscopy and electrophysiology^65,119–124^. Details of the quantification method have been described previously^81^. Briefly, 1 µm thick maximum projections are separated into red and green channels, backgrounds are subtracted (rolling ball radius = 50), and thresholds are determined to detect discrete puncta without introducing noise. The minimum pixel size of puncta was set as 4 to remove any background noise. The Puncta Analyzer plugin then uses an algorithm to detect the number of puncta that are in close proximity across the two channels, yielding quantified co-localized puncta.

### Adeno-associated virus (AAV) production

AAV-EF1α-WGA-Dre and AAV-EF1α-(N)Cre-Rox-Stop-Rox-(C)Cre, were produced and purified as described before^82^. More in detail, AAVs were produced by co-transfecting each AAV vector (15 µg) to the HEK 293 T cells (from ATCC #CRL−11268) with a helper (pAD-delta F6, 30 µg) and the capsid plasmids (15 µg). Five 15-cm tissue culture dishes (12 × 10^6^ HEK 293T cells per dish) were used to produce one type of virus. Three days post-transfection, HEK 293 T cells were lysed using the cell lysis buffer (Cell lysis buffer: Add 3 ml of 5 M NaCl and 5 ml of 1 M Tris-HCl (pH 8.5) to 80 ml of dH_2_O. Adjust the pH to 8.5 with NaOH and adjust the volume to 100 ml with dH_2_O. Sterilize by passing through a 0.22-μm filter and store at 4 °C). Then the AAV particles were released from the cells by three cycles of freeze/thaw between dry ice-ethanol and 37 °C water bath and then treated with Benzonase (Novagen, 70664) at 37 °C for 30 min. Then AAVs were purified using Iodixanol Gradient Ultracentrifugation as described by Addgene (https://www.addgene.org/protocols/aav-purification-iodixanol-gradient-ultracentrifugation/). Briefly, the cell lysates were loaded to the gradients of iodixanol (Sigma D1556, 15%, 25%, 40%, and 60%) in the OptiSeal tubes (Beckman Coulter, Indianapolis, IN). Then the lysates were spun down at 462,000 g for 1 h at 18 °C with the ultracentrifuge (Beckman) using a Beckman 70 Ti rotor. The AAV particles were collected by collecting the interface between 40 and 60% iodixanol with a syringe. The collected AAV-containing solution was mixed with ice-cold DPBS and concentrated using the Vivaspin column (100 MWCO) at 4 °C. Collected virus particles were aliquoted and stored at −80 °C until use.

### Surgery procedure

Mice were anesthetized with 1.5–2.0% isoflurane mixed with 0.60 L/min of oxygen for surgical procedures and placed into a stereotactic frame (David Kopf Instruments, Tujunga, CA). Meloxicam (2 mg/kg) and topical bupivacaine (0.20 mL) were administered prior to incision. α2δ−1(f/f) and α2δ−1(+/+) mice were used for the behavioral and electrophysiological test as well as for anatomical and synaptic count studies. For these purposes, 50 nL of AAV-EF1α-WGA-Dre were injected bilaterally in the dorsomedial striatum (AP: +0.5 relative to bregma, ML: 1.4 relative to bregma, DV: 2.0 relative to brain surface) and 100 nL of AAV-EF1α-(N)Cre-Rox-Stop-Rox-(C)Cre were injected bilaterally in the frontal and caudal part of the Anterior Cingulate Cortex (AP: +1.5 relative to bregma, ML: 0.12 relative to bregma, DV: 1.5 relative to brain surface; AP: −0.2 relative to bregma, ML: 0.12 relative to bregma, DV: 1.0 relative to brain surface) using a microinjector (Nanoject 3000, Drummond Scientific) at a rate of 1 nL/s.

For optogenetic stimulation experiments, 200 nL of AAV-EF1α-DIO-hChR2(E123T/T15 9 C)-eYFP (UNC viral vector core) or AAV-hSyn-DIO-BLINK2-eGFP (Duke viral vector core) were bilaterally injected into the ACC (AP: +0.9 relative to bregma, ML: 0.12 relative to bregma, DV: 1.1 relative to brain surface) and 200 nL of AAV(RETRO2)-EF1α-Cre-WPRE (Duke viral vector core) were bilaterally injected into the DMS (AP: +0.5 relative to bregma, ML: 1.4 relative to bregma, DV: 2.0 relative to brain surface) of WT, α2δ−1(f/f) or α2δ−1(+/+) mice. Custom-made optic fibers (2–3 mm length below ferrule, >75% transmittance, 105 μm core diameter) were then implanted directly above the ACC at an angle (AP: +0.5 with respect to bregma, ML: 1.1 with respect to bregma, DV: 1.3 from the brain surface; 25° angle). Fibers were secured in place with dental acrylic adhered to skull screws. Mice were allowed to recover for three weeks after surgery before experimentation.

### Optogenetic stimulation

For optogenetic stimulations, WT, α2δ−1 (f/f), and α2δ−1 (+/+) mice expressing ChR2 and implanted with optic fibers were trained for the LP task as described above. After the 3 days FR1, mice were trained with the optic fibers attached to the laser source to allow habituation. The training was conducted as described above for the 9 days and in addition, we performed 2 more days with an FR20 schedule. On day 12 the laser was turned on at a frequency of 20 Hz and a power of 7 mW to allow light stimulation for 5 min soon after 5 min without light stimulation. This cycle was repeated three times for a total of 30 min to allow a within-session comparison of the light Off and light On periods. For the PR experiments, mice were trained up to FR10 and on the next day went through either a PR test with optogenetic stimulation or without. After retraining to FR10, the same mice were then tested again on PR with the opposite optogenetic condition. This approach allowed us to compare the same mice across sessions (light Off and light On) and to counterbalance the session for the order in which they occurred. The modulation index has been calculated using the following equation: $\frac{({LPon}-{LPoff})}{({LPon}+{LPoff})}$.

### Circuit tracing and fiber implant control

The circuit tracing and surgery check were conducted as follows. After the behavioral tests were concluded, mice were anesthetized with 200 mg/kg tribromoethanol (avertin) and euthanized by perfusing with a solution made of TBS with heparin (0.1128 g Heparin ammonium salt from porcine intestinal mucosa [Sigma; H6279]) and then with 4% Paraformaldehyde (PFA). Mouse brains were then kept in 4% PFA o.n. at 4 °C. The day after brains were rinsed three times with TBS, immersed in 30% Sucrose in TBS, and stored at 4 °C until they were not floating anymore in the solution. At this time brains were included in a mixture of 30% Sucrose in TBS and Tissue Tek O.C.T. compound (frozen tissue matrix) at a 2:1 ratio and stored at −80 °C. Brains were cut into 20 or 50-μm coronal sections and stored in a 1:1 mixture of TBS/glycerol at −20 °C. Sections were washed in 1× TBS containing 0.2% Triton-X100 (TBST) and blocked in 5% NGS diluted in TBST. For the mice expressing ChR2, BLINK2 or their control with only fiber implants, sections were incubated o.n. with a primary antibody chicken anti-GFP (1:1000; Millipore, AB16901; Aves Labs GFP 1010). In addition, brain sections from α2δ−1 (f/f) and α2δ−1 (+/+) mice, were also incubated with a primary antibody rabbit anti-RFP (1:1000; Rockland, 600-401-379). Secondary Alexa-fluorophore (488, 594) conjugated antibodies (Invitrogen) were added (1:200 in TBST with 5% NGS) for 2 h at room temperature. Slides were mounted in Vectashield with DAPI (Vector Laboratories, CA) and images were acquired on an Olympus Fluoview confocal microscope using ×20 objective at ×1.3 Zoom. Mice were excluded if fiber placement was not located in the target site.

### Whole-cell patch-clamp recording

For whole-cell patch-clamp recordings, four trained and four untrained mice were used per group (WT or α2δ−1 (f/f)) to measure miniature excitatory postsynaptic current (mEPSC) before or after training. During all recordings, brain slices were continuously perfused with standard aCSF at RT (∼25 °C) and visualized by an upright microscope (BX61WI, Olympus) through a 40x water-immersion objective equipped with infrared-differential interference contrast optics in combination with digital camera (ODA-IR2000WCTRL). Patch-clamp recordings were performed by using an EPC 10 patch-clamp amplifier, controlled by Patchmaster Software (HEKA). Data were acquired at a sampling rate of 50 kHz and low-pass filtered at 6 kHz.

To prepare acute brain slices, after decapitation, the brains were immersed in ice-cold artificial cerebrospinal fluid (aCSF, in mM): 125 NaCl, 2.5 KCl, 3 mM MgCl_2_, 0.1 mM CaCl_2_, 10 glucose, 25 NaHCO_3_, 1.25 NaHPO_4_, 0.4 l-ascorbic acid, and 2 Na-pyruvate, pH 7.3–7.4 (310 mOsm). Coronal slices containing the ACC were obtained using a vibrating tissue slicer (Leica VT1200; Leica Biosystems). Slices were immediately transferred to standard aCSF (33 °C, continuously bubbled with 95% O_2_ – 5% CO_2_) containing the same as the low-calcium aCSF but with 1 mM MgCl_2_ and 1–2 mM CaCl_2_. After 30 min incubation, slices were transferred to a recording chamber with the same extracellular buffer at room temperature (RT: ∼25 °C).

To measure mEPSCs, the internal solution contained the following (in mM): 125 K-gluconate, 10 NaCl, 10 HEPES, 0.2 EGTA, 4.5 MgATP, 0.3 NaGTP, and 10 Na-phosphocreatine, pH adjusted to 7.2–7.4 with KOH and osmolality set to ~300 mOsm. mEPSCs were measured in the aCSF bath solution containing 1 µM tetrodotoxin and 50 µM Picrotoxin at -70 mV in voltage-clamp mode. mEPSCs recorded at −70 mV were detected using Minhee Analysis software (https://github.com/parkgilbong/Minhee_Analysis_Pack). To analyze the frequency, events were counted over 5 min of recording. To obtain the average events for each cell, at least 100 non-overlapping events were detected and averaged. The peak amplitude of the average mEPSCs was measured relative to the baseline current.

For the data collected only after training, eight mice were used to measure excitability and seven mice to measure miniature excitatory postsynaptic currents (mEPSCs). Viral injection to label ACC_→DMS_ neurons was performed as described previously, and the mice were sacrificed 6 weeks after the injection. The brain was removed quickly and placed in ice-cold solution bubbled with 95% O_2_–5% CO_2_ containing the following (in mM): 194 sucrose, 30 NaCl, 2.5 KCl, 1 MgCl_2_, 26 NaHCO_3_, 1.2 NaH_2_PO_4_, and 10 d-glucose. After 5 min, 250 μm coronal slices were cut with a vibratome (PELCO) and then placed in 35.5 °C oxygenated artificial cerebrospinal fluid (aCSF) solution containing the following (in mM): 124 NaCl, 2.5 KCl, 2 CaCl_2_, 1 MgCl_2_, 26 NaHCO_3_, 1.2 NaH_2_PO_4_, and 10 d-glucose, pH adjusted to 7.4 with HCl and osmolality set to ~310 mosM. After 30 min, the slices were maintained in aCSF at ~22–23 °C for at least 30 min before recording. Following recovery, all recordings were conducted under continuous perfusion of aCSF at 29–30 °C, and the pipette’s impedances were between 3.5 and 5 MΩ. All recordings were performed with MultiClamp 700B amplifier (Molecular Device) and filtered at 10 kHz and digitized at 20 kHz with a Digidata 1440 A digitizer (Molecular Devices).

To measure the excitability, the internal solution contained (in mM) 150 potassium gluconate, 2 MgCl_2_, 1.1 EGTA, 10 HEPES, 3 sodium ATP, and 0.2 sodium GTP, with pH adjusted to 7.2 with KOH and osmolarity set to ∼300 mOsm. Pipette impedances were between 3.5 and 5 MΩ. The excitability was measured in current-clamp mode by injection of current between −300 and 400 pA. Each step was 100 pA with a duration of 1 s. The number of spikes for each depolarizing step was counted by peak detection software in pCLAMP10 (Molecular Devices).

To measure miniature excitatory postsynaptic current (mEPSC), the internal solution contained the following (in mM): 120 cesium methanesulfonate, 5 NaCl, 10 tetraethylammonium chloride, 10 HEPES, 4 lidocaine N-ethyl bromide, 1.1 EGTA, 4 magnesium ATP, and 0.3 sodium GTP, pH adjusted to 7.2 with CsOH and osmolality set to ~300 mosM. mEPSCs were measured in the aCSF bath solution containing 1 µM tetrodotoxin and 50 µM Picrotoxin at −70 mV in voltage-clamp mode. The amplitudes of mEPSCs over −10 pA by the peak detection software in pCLAMP10 were counted.

Further information and requests for resources and reagents can be directed to the Lead Contacts, Francesco Paolo Ulloa Severino (francesco.ulloa@cajal.csic.es), Henry Yin (hy43@duke.edu), and Cagla Eroglu (cagla.eroglu@duke.edu).

### Quantification and statistical analysis

All statistical analyses were performed in GraphPad Prism v8 and v9. Sample size and specific statistical tests for each experiment are indicated in the figure legend for each experiment. Exact adjusted *P* values are listed in the figures for each experiment, where absent the difference was not significant (*P* > 0.05). The details of the statistical analysis are reported in figure legends. The significance for all the quantifications is *P < 0.05, **P < 0.01, ***P < 0.001, and ****P < 0.0001. The RNA sequencing data were analyzed using the Benjamini–Hochberg correction and the false discovery rate (FDR) was utilized to evaluate differences among data sets. Where indicated, Unpaired two-tailed *t* test were run using Welch’s correction and then a correction for multiple comparison was applied using Hom–Sidak method with an alpha threshold of 0.05 for adjusted *P* value. A Geisser-Greenhouse correction was used for both one-way and two-way ANOVA analyses. Sample sizes were determined based on previous experience for each experiment to yield high power to detect specific effects. No statistical methods were used to predetermine sample size.

### Statistics and reproducibility

Here we report the statistical details of each experiment, organized by figure number, whenever not present in the figure legend.

Figure 1: **c** (Day 1 = 3 ± 0.4 LP/min, Day 9 = 22 ± 1.9 LP/min; one-way ANOVA for repeated measures, main effects of Days [*F* (8, 128) = 74.86, *P* < 0.0001] and Subject [*F* (2.13, 17.04) = 10.30, *P* = 0.001])., **f** ACC (176.203 ± 8.26%, [*t* (9.684) = 5.906], *P* = 0.003). *n* = 6 mice per condition, except for the following regions due to their absence in some cases: AI (*n* = 3 untrained; *n* = 4 trained), BMA (*n* = 6 untrained; *n* = 4 trained), COA (*n* = 5 untrained; *n* = 5 trained), ORB (*n* = 5 untrained; *n* = 6 trained), PL (*n* = 6 untrained, *n* = 5 trained), RE (*n* = 5 untrained; *n* = 6 trained). Multiple unpaired *t* test with Welch correction. Multiple comparisons using Holm–Sidak method; alpha = 0.05 for adjusted *P* value. Data shown as mean ± s.e.m.

Figure 2: **b** (*n* = 6 mice per condition (3 males and 3 females); 4 sections per mouse). Multiple unpaired t-test with Welch correction. Multiple comparisons using Holm–Sidak method; adjusted *P* value. vACC 2/3 [t (9.18) = 3.72]. **e** For L1 and L5 (*n* = 6 mice per condition, 3 images per mouse); for L2/3 (*n* = 5 mice per condition, 3 images per mouse). Multiple unpaired *t* test with Welch correction. Multiple comparisons using Holm–Sidak method; adjusted *P* value. L1 Untrained (89.1 ± 3.02), Trained (98.3 ± 2.97) [*t* (34) = 2.17]; L2/3 Untrained (81.0 ± 5.34), Trained (107 ± 7.92) [t (24.5) = 2.7]; L5 Untrained (124 ± 5.58), Trained (140 ± 6.87) [t (33) = 1.86]. **g** sections have been imaged from 24 mice. **i** Comparison between Untrained (*n* = 12 cells from 4mice) and Trained (*n* = 11 cells from 4 mice) WT mice. Kolmogorov–Smirnov test (*P* = 0.0008); average mEPSC frequency, Untrained (0.84 ± 0.11) and Trained (1.45 ± 0.20). Unpaired two-tailed *t* test [t (20) = 2.78] *P* = 0.011. **j** Comparison between Untrained (*n* = 12 cells from 4mice) and Trained (*n* = 11 cells from 4 mice) WT mice. Kolmogorov–Smirnov test (*P* = 0.474); average mEPSC amplitude, Untrained (6.59 ± 0.81) and Trained (6.05 ± 0.49). Unpaired two-tailed *t* test [*t* (21) = 0.56] *P* = 0.584. Data shown as mean ± s.e.m.

Figure 3: **c** Multiple unpaired *t* test with Welch correction. Multiple comparisons using Holm–Sidak method; alpha = 0.05 for adjusted *P* value. L1 (WT 89 ± 3.0; KO 50 ± 4.8), [*t* (19.5) = 6.89, *P* = 0.000001] and L2/3 (WT 81 ± 2.7; KO 34 ± 2.7), [*t* (20.3) = 7.83, *P* < 0.000001]. For L1 (WT: *n* = 6 mice, 3 images per mouse; KO: *n* = 4 mice, 3 images per mouse); for L2/3 (WT: *n* = 5 mice, 3 images per mouse; KO: *n* = 4 mice, 3 images per mouse). **d** Multiple unpaired *t* test with Welch correction. Multiple comparisons using Holm–Sidak method; alpha = 0.05 for adjusted *P* value. L1 (Untrained 50 ± 4.8; Trained 58 ± 9.1) [*t* (16.5) = 0.77]. L2/3 (Untrained 34 ± 2.7; Trained 43 ± 4.8), [*t* (17.3) = 1.5] *n* = 4 mice per condition, 3 images per mouse. **f** Two-way ANOVA. Main effects of Genotype [*F* (1,16) = 225.0, *P* < 0.0001], main effect of Training [*F* (1, 16) = 20.93, *P* = 0.0003], and significant interaction [*F* (1,16) = 20.52, *P* = 0.0003]. Tukey’s multiple comparisons analysis reported in the figure. WT Untrained vs Trained (*n* = 6 mice per condition) [*q* (16) = 10.18; *P* < 0.0001]; KO Untrained vs Trained (*n* = 4 mice per condition) [*q* (16) = 0.041; *P* > 0.9999]. Data shown as mean ± s.e.m.

Figure 4: **b** RM two-way ANOVA. Main effects of Days [*F* (2.536, 114.1) = 196.4, *P* < 0.0001], no effect of Genotype [*F* (1, 45) = 0.04814, *P* = 0.827] nor interaction [*F* (8, 360) = 0.4819, *P* = 0.869]. **e** Two-way ANOVA for repeated measures. Main effects of Genotype [*F* (1, 39) = 8.060] and Time [*F* (1.683, 65.65) = 636.6, *P* < 0.0001] and interaction [*F* (12, 468) = 7.220, *P* < 0.0001]. **f** Unpaired two-tailed *t* test [*t* (39) = 4.4; *P* < 0.0001]. **g** RM two-way ANOVA. Main effect of time [*F* (8.158, 318.1) = 31.66, *P* < 0.0001], no effect of genotype [*F* (1, 39) = 4.077, *P* = 0.050] and no interaction [*F* (19, 741) = 0.9839, *P* = 0.478]. **h** RM two-way ANOVA. Main effect of time [*F* (3.755, 86.36) = 47.89, *P* < 0.0001], no effect of genotype [*F* (1, 23) = 0.4091, *P* = 0.528] and no interaction [*F* (11, 253) = 0.2691, *P* = 0.991]. **j** WT (*n* = 10 mice), KO (*n* = 7 mice). RM two-way ANOVA. Main effect of value state [*F* (1, 15) = 11.73], no effect of genotype [*F* (1, 15) = 4.44, *P* = 0.052] nor interaction [*F* (1, 15) = 0.0224, *P* = 0.883]. Data shown as mean ± s.e.m.

Figure 5: **b** RM two-way ANOVA. *n* = 12 mice per condition. Main effect of Days [*F* (1.795, 39.50) = 55.72, *P* < 0.0001], no effect of genotype [*F* (1, 22) = 2.851, *P* = 0.105] nor interaction [*F* (8, 176) = 1.943, *P* = 0.056]. *n* = 12; 6 male and 6 female per group. **e** Two-way ANOVA for repeated measures. Main effects of Genotype [*F* (1, 22) = 8.832] and time [*F* (12, 264) = 298.4, *P* < 0.0001] and interaction [*F* (12, 264) = 10.62, *P* < 0.0001]. α2δ−1 (+/+) (*n* = 12; 18.5 ± 1.2 rewards) and α2δ−1(f/f) animals (*n* = 12; 25.8 ± 1.8 rewards). **f** Unpaired *t* test [*t* (22) = 3.366, *P* = 0.003]. α2δ−1 (+/+) (*n* = 12; Breakpoint = 88.5 ± 6.1) and α2δ−1(f/f) (*n* = 12; Breakpoint = 125.2 ± 9.0) animals. **g** Two-way ANOVA for repeated measures. Main effects of Time [*F* (5.433, 119.5) = 13.14, *P* < 0.0001], no effect of genotype [*F* (1, 22) = 0.1059, *P* = 0.748] nor interaction [*F* (19, 418) = 1.399, *P* = 0.122] *n* = 12 animals per group. **h** For α2δ−1 (+/+) (*n* = 9) and for α2δ−1 (f/f) (*n* = 11). RM two-way ANOVA. Main effect of time [*F* (2.269, 40.85) = 15.81, *P* < 0.0001], no effect of genotype [*F* (1, 18) = 0.7326, *P* = 0.403] and significant interaction [*F* (11, 198) = 1.893, *P* = 0.042]. Multiple comparison showed no differences between genotypes at any time point. **j** α2δ−1 (+/+) (*n* = 9 mice); α2δ−1 (f/f) (*n* = 8 mice). RM two-way ANOVA. Main effect of value state [*F* (1, 15) = 64.07], no effect of genotype [*F* (1, 15) = 0.44, *P* = 0.514] and interaction [*F* (1, 15) = 4.73, *P* = 0.046]. Multiple comparison showed significant difference between devalued and valued states for α2δ−1 (+/+) [*P* = 0.001] and α2δ−1(f/f) [*P* < 0.0001]. Data shown as mean ± s.e.m.

Figure 6: **c** α2δ−1(+/+) (*n* = 4 mice, 19 cells) and α2δ−1(f/f) (*n* = 4 mice, 16 cells). RM two-way ANOVA. Main effect of Current [*F* (2.3, 75.91) = 221.6, *P* < 0.0001], no effect of genotype [*F* (1, 33) = 0.5398, *P* = 0.467] and no interaction [*F* (4, 132) = 0.7386, *P* = 0.567]. **d** α2δ−1(+/+) (*n* = 4 mice, 19 cells) and α2δ−1(f/f) (*n* = 4 mice, 16 cells). Unpaired *t* test, [*t* (33) = 1.416, *P* = 0.166]. **f** Two-way ANOVA. Main effect of genotype [*F* (1, 32) = 39.73, *P* < 0.0001], Training [*F* (1, 32) = 46.97, *P* < 0.0001] and interaction [*F* (1, 32) = 6.473, *P* = 0.016]. Multiple comparison showed in figure for relevant comparisons. *n* = 3 mice per condition and genotype (*n* = 3 images per mouse). **h** Left: α2δ−1(+/+) (*n* = 4 mice; 20 cells) and α2δ−1(f/f) (*n* = 3 mice; 18 cells) mice. Kolmogorov–Smirnov test. Right: α2δ−1(+/+) (*n* = 4 mice, 20 cells; 0.64 ± 0.08 Hz) and α2δ−1(f/f) (*n* = 3 mice, 18 cells; 0.41 ± 0.06 Hz) mice. Unpaired *t* test [*t* (36) = 2.27]. **i** Left: α2δ−1(+/+) (*n* = 4 mice; 20 cells) and α2δ−1(f/f) (*n* = 3 mice; 18 cells) mice. Kolmogorov–Smirnov test [*P* = 0.176]. Right: α2δ−1(+/+) (*n* = 4 mice, 20 cells; 14 ± 0.38 pA) and α2δ−1(f/f) (*n* = 3 mice, 18 cells; 13 ± 0.34 pA) mice. Unpaired *t* test [*t* (36) = 1.00, *P* = 0.322]. **k** Left: α2δ−1(f/f) untrained (*n* = 10 cells from 4 mice) and trained (*n* = 10 cells from 4 mice) mice. Kolmogorov–Smirnov test (*P* = 0.723). Right: Untrained (0.92 ± 0.23) and Trained (0.84 ± 0.12) α2δ−1(f/f) mice. Unpaired two-tailed *t* test [*t* (18) = 0.296]. **l** Left: untrained (*n* = 10 cells from 4 mice) and trained (*n* = 10 cells from 4 mice) α2δ−1(f/f) mice. Kolmogorov–Smirnov test (*P* = 0.705). Right: untrained (7.6 ± 0.49) and Trained (7.5 ± 0.19) α2δ−1(f/f) mice. Unpaired *t* test [*t* (18) = 0.064, *P* = 0.949]. Data shown as mean ± s.e.m. alpha = 0.05.

Figure 8: **c** *n* = 10 mice. Paired two-tailed *t* test [*t* (9) = 3.2]. **d** *n* = 10 mice. RM two-way ANOVA, main effect of stimulation [*F* (1, 18) = 5.219] and time [*F* (2, 36) = 161.1, *P* < 0.0001], no interaction [*F* (2, 36) = 0.6485, *P* = 0.528]. **e** Paired two-tailed *t* test [*t* (9) = 3.3]. **g** *n* = 7 mice. Paired two-tailed *t* test [*t* (6) = 3.7]. **h** RM two-way ANOVA, main effect of stimulation [*F* (1, 12) = 10.21; *P* = 0.007] and time [*F* (1.458, 17.49) = 267.4, *P* < 0.0001], significant interaction [*F* (2, 24) = 5.379, *P* = 0.012]. **i** Paired two-tailed *t* test [*t* (6) = 3.2]. **j** Unpaired two-tailed *t* test [t (15) = 5.1; *P* = 0.0001]. For all graphs: Multiple comparisons using Holm–Sidak method; alpha = 0.05 for adjusted *P* value. Data shown as mean ± s.e.m.

### Reporting summary

Further information on research design is available in the Nature Portfolio Reporting Summary linked to this article.

### Supplementary information

Supplementary Information

Reporting Summary

### Source data

Source Data

## Data Availability

The reagents and data generated in this study are available without restriction. RNA-sequencing data have been deposited in the Gene Expression Omnibus (GEO) repository with accession number: GSE169392. The accession code for the mouse genome used for the RNA-seq is GRCm38, available at https://www.ncbi.nlm.nih.gov/datasets/genome/GCF_000001635.20/. The scRNAseq database from ref. ^86^ can be found at http://dropviz.org/. Source data are provided with this paper.

## Code availability

The scripts generated in this study are available without restriction. Please visit https://github.com/ErogluLab/CellCounts, https://github.com/UlloaSeverinoLab to access the custom codes used in this manuscript. We recommend getting in touch with the corresponding authors to have further information.

## References

[1] Yin HH, Knowlton BJ (2006). The role of the basal ganglia in habit formation. Nat. Rev. Neurosci..

[2] Hartley SL, Sikora DM, McCoy R (2008). Prevalence and risk factors of maladaptive behaviour in young children with autistic disorder. J. Intellect. Disabil. Res. JIDR.

[3] Botvinick MM, Huffstetler S, McGuire JT (2009). Effort discounting in human nucleus accumbens. Cogn. Affect. Behav. Neurosci..

[4] Bayés À (2011). Characterization of the proteome, diseases and evolution of the human postsynaptic density. Nat. Neurosci..

[5] Chih B, Engelman H, Scheiffele P (2005). Control of excitatory and inhibitory synapse formation by neuroligins. Science.

[6] Robbins EM (2010). SynCAM 1 adhesion dynamically regulates synapse number and impacts plasticity and learning. Neuron.

[7] Connor SA (2017). Loss of synapse repressor MDGA1 enhances perisomatic inhibition, confers resistance to network excitation, and impairs cognitive function. Cell Rep..

[8] Guo B (2019). Anterior cingulate cortex dysfunction underlies social deficits in Shank3 mutant mice. Nat. Neurosci..

[9] Connor SA, Elegheert J, Xie Y, Craig AM (2019). Pumping the brakes: suppression of synapse development by MDGA–neuroligin interactions. Curr. Opin. Neurobiol..

[10] Assous M (2019). Neuropilin 2 signaling mediates corticostriatal transmission, spine maintenance, and goal-directed learning in mice. J. Neurosci..

[11] Ribic A, Crair MC, Biederer T (2019). Synapse-selective control of cortical maturation and plasticity by parvalbumin-autonomous action of SynCAM 1. Cell Rep..

[12] Fernández-García S (2020). M2 cortex-dorsolateral striatum stimulation reverses motor symptoms and synaptic deficits in Huntington’s disease. eLife.

[13] Zhu F (2018). Architecture of the mouse brain synaptome. Neuron.

[14] Cizeron M (2020). A brainwide atlas of synapses across the mouse life span. Science.

[15] Curran OE, Qiu Z, Smith C, Grant SGN (2021). A single-synapse resolution survey of PSD95-positive synapses in twenty human brain regions. Eur. J. Neurosci..

[16] Xu T (2009). Rapid formation and selective stabilization of synapses for enduring motor memories. Nature.

[17] Yang G, Pan F, Gan W-B (2009). Stably maintained dendritic spines are associated with lifelong memories. Nature.

[18] Penzes P, Cahill ME, Jones KA, VanLeeuwen J-E, Woolfrey KM (2011). Dendritic spine pathology in neuropsychiatric disorders. Nat. Neurosci..

[19] Chen, H. I. et al. Neural substrate expansion for the restoration of brain function. *Front. Syst. Neurosci*. **10**, 1 (2016). 10.3389/fnsys.2016.00001 PMC4724716 26834579

[20] Li, D. C. et al. A molecularly integrated amygdalo-fronto-striatal network coordinates flexible learning and memory. *Nat. Neurosci*. 1–12 10.1038/s41593-022-01148-9 (2022). 10.1038/s41593-022-01148-9 PMC10614133 36042313

[21] Hedrick NG (2022). Learning binds new inputs into functional synaptic clusters via spinogenesis. Nat. Neurosci..

[22] Xiong Q, Znamenskiy P, Zador AM (2015). Selective corticostriatal plasticity during acquisition of an auditory discrimination task. Nature.

[23] Adamsky, A. et al. Astrocytic activation generates de novo neuronal potentiation and memory enhancement. *Cell* **174**, 59–71 (2018). 10.1016/j.cell.2018.05.002 29804835

[24] Doron A, Goshen I (2018). Investigating the transition from recent to remote memory using advanced tools. Brain Res. Bull..

[25] Rossi, M. A. & Yin, H. H. Methods for studying habitual behavior in mice. *Curr. Protoc. Neurosci.* **60**, 8–29 (2012). 10.1002/0471142301.ns0829s60 PMC3408711 22752897

[26] Jin X, Costa RM (2010). Start/stop signals emerge in nigrostriatal circuits during sequence learning. Nature.

[27] Coutureau, E., Esclassan, F., Di Scala, G. & Marchand, A. R. The role of the rat medial prefrontal cortex in adapting to changes in instrumental contingency. *PLoS ONE* **7**, e33302 (2012). 10.1371/journal.pone.0033302 PMC3319541 22496747

[28] Jin X, Tecuapetla F, Costa RM (2014). Basal ganglia subcircuits distinctively encode the parsing and concatenation of action sequences. Nat. Neurosci..

[29] Yin HH (2009). Dynamic reorganization of striatal circuits during the acquisition and consolidation of a skill. Nat. Neurosci..

[30] Gremel CM, Costa RM (2013). Orbitofrontal and striatal circuits dynamically encode the shift between goal-directed and habitual actions. Nat. Commun..

[31] Hart G, Bradfield LA, Fok SY, Chieng B, Balleine BW (2018). The bilateral prefronto-striatal pathway is necessary for learning new goal-directed actions. Curr. Biol..

[32] Yin HH, Ostlund SB, Knowlton BJ, Balleine BW (2005). The role of the dorsomedial striatum in instrumental conditioning. Eur. J. Neurosci.

[33] Hintiryan H (2016). The mouse cortico-striatal projectome. Nat. Neurosci..

[34] Gao L (2022). Single-neuron projectome of mouse prefrontal cortex. Nat. Neurosci..

[35] Swanson AM, DePoy LM, Gourley SL (2017). Inhibiting Rho kinase promotes goal-directed decision making and blocks habitual responding for cocaine. Nat. Commun..

[36] Hart G, Bradfield LA, Balleine BW (2018). Prefrontal corticostriatal disconnection blocks the acquisition of goal-directed action. J. Neurosci..

[37] Gourley SL, Zimmermann KS, Allen AG, Taylor JR (2016). The medial orbitofrontal cortex regulates sensitivity to outcome value. J. Neurosci..

[38] Alabi OO (2020). Disruption of Nrxn1α within excitatory forebrain circuits drives value-based dysfunction. eLife.

[39] Schweimer J, Hauber W (2005). Involvement of the rat anterior cingulate cortex in control of instrumental responses guided by reward expectancy. Learn. Mem..

[40] Schweimer J, Hauber W (2006). Dopamine D1 receptors in the anterior cingulate cortex regulate effort-based decision making. Learn. Mem..

[41] Tinaz S (2020). Goal-directed behavior in individuals with mild Parkinson’s disease: Role of self-efficacy and self-regulation. Clin. Park. Relat. Disord..

[42] Nelson SB, Valakh V (2015). Excitatory/inhibitory balance and circuit homeostasis in autism spectrum disorders. Neuron.

[43] Sohal VS, Rubenstein JLR (2019). Excitation-inhibition balance as a framework for investigating mechanisms in neuropsychiatric disorders. Mol. Psychiatry.

[44] Maia TV, Cooney RE, Peterson BS (2008). The neural bases of obsessive-compulsive disorder in children and adults. Dev. Psychopathol..

[45] Sagar SM, Sharp FR, Curran T (1988). Expression of c-fos protein in brain: metabolic mapping at the cellular level. Science.

[46] Bertaina‐Anglade V, Tramu G, Destrade C (2001). Differential learning‐stage dependent patterns of c‐Fos protein expression in brain regions during the acquisition and memory consolidation of an operant task in mice. Eur. J. Neurosci..

[47] Yap E-L, Greenberg ME (2018). Activity-regulated transcription: bridging the gap between neural activity and behavior. Neuron.

[48] Yap E-L (2021). Bidirectional perisomatic inhibitory plasticity of a Fos neuronal network. Nature.

[49] Cohen JD, Botvinick M, Carter CS (2000). Anterior cingulate and prefrontal cortex: who’s in control?. Nat. Neurosci..

[50] Matsumoto K, Tanaka K (2004). Conflict and cognitive control. Science.

[51] Shenhav A, Botvinick MM, Cohen JD (2013). The expected value of control: an integrative theory of anterior cingulate cortex function. Neuron.

[52] Shenhav A, Cohen JD, Botvinick MM (2016). Dorsal anterior cingulate cortex and the value of control. Nat. Neurosci..

[53] McKee BL, Kelley AE, Moser HR, Andrzejewski ME (2010). Operant learning requires NMDA-receptor activation in the anterior cingulate cortex and dorsomedial striatum, but not in the orbitofrontal cortex. Behav. Neurosci..

[54] Hayden BY, Platt ML (2010). Neurons in anterior cingulate cortex multiplex information about reward and action. J. Neurosci..

[55] Walton ME, Bannerman DM, Rushworth MFS (2002). The role of rat medial frontal cortex in effort-based decision making. J. Neurosci..

[56] Walton ME, Bannerman DM, Alterescu K, Rushworth MFS (2003). Functional specialization within medial frontal cortex of the anterior cingulate for evaluating effort-related decisions. J. Neurosci..

[57] Hart, E. E., Blair, G. J., O’Dell, T. J., Blair, H. T. & Izquierdo, A. Chemogenetic modulation and single-photon calcium imaging in anterior cingulate cortex reveal a mechanism for effort-based decisions. *J. Neurosci*. 10.1523/JNEUROSCI.2548−19.2020 (2020). 10.1523/JNEUROSCI.2548-19.2020 PMC7363467 32527984

[58] van der Veen B (2021). Control of impulsivity by Gi-protein signalling in layer-5 pyramidal neurons of the anterior cingulate cortex. Commun. Biol..

[59] Tervo, D. G. R. et al. The anterior cingulate cortex directs exploration of alternative strategies. *Neuron* **109**, 1876–1887 (2021). 10.1016/j.neuron.2021.03.028 33852896

[60] Risher WC, Eroglu C (2020). Emerging roles for α2δ subunits in calcium channel function and synaptic connectivity. Curr. Opin. Neurobiol..

[61] Risher, W. C. et al. Thrombospondin receptor α2δ−1 promotes synaptogenesis and spinogenesis via postsynaptic Rac1. *J. Cell Biol*. jcb.201802057 10.1083/jcb.201802057 (2018). 10.1083/jcb.201802057 PMC6168259 30054448

[62] Cole RL (2005). Differential distribution of voltage-gated calcium channel alpha-2 delta (α2δ) subunit mRNA-containing cells in the rat central nervous system and the dorsal root ganglia. J. Comp. Neurol..

[63] Dolphin AC (2013). The α2δ subunits of voltage-gated calcium channels. Biochim. Biophys. Acta BBA Biomembr..

[64] Tong X-J (2017). Retrograde synaptic inhibition is mediated by α-neurexin binding to the α2δ subunits of N-type calcium channels. Neuron.

[65] Eroglu Ç (2009). Gabapentin receptor α2δ−1 is a neuronal thrombospondin receptor responsible for excitatory CNS synaptogenesis. Cell.

[66] Kurshan PT, Oztan A, Schwarz TL (2009). Presynaptic α 2 δ-3 is required for synaptic morphogenesis independent of its Ca 2+ -channel functions. Nat. Neurosci..

[67] Held, R. G. et al. Synapse and active zone assembly in the absence of presynaptic Ca2+ channels and Ca2+ entry. *Neuron*10.1016/j.neuron.2020.05.032 (2020). 10.1016/j.neuron.2020.05.032 PMC7442750 32616470

[68] Bertaina V, Destrade C (1995). Differential time courses of c-fos mRNA expression in hippocampal subfields following acquisition and recall testing in mice. Cogn. Brain Res..

[69] Fürth D (2018). An interactive framework for whole-brain maps at cellular resolution. Nat. Neurosci..

[70] Friedman A (2015). A corticostriatal path targeting striosomes controls decision-making under conflict. Cell.

[71] Marton TF, Seifikar H, Luongo FJ, Lee AT, Sohal VS (2018). Roles of prefrontal cortex and mediodorsal thalamus in task engagement and behavioral flexibility. J. Neurosci..

[72] Brockett, A. T., Tennyson, S. S., deBettencourt, C. A., Gaye, F. & Roesch, M. R. Anterior cingulate cortex is necessary for adaptation of action plans. *Proc. Natl. Acad. Sci*. *USA*10.1073/pnas.1919303117 (2020). 10.1073/pnas.1919303117 PMC7084129 32132213

[73] Lin Y (2008). Activity-dependent regulation of inhibitory synapse development by Npas4. Nature.

[74] Liu X (2012). Optogenetic stimulation of a hippocampal engram activates fear memory recall. Nature.

[75] Poirier, R. et al. Distinct functions of Egr gene family members in cognitive processes. *Front. Neurosci*. **2**, 242 (2008). 10.3389/neuro.01.002.2008 PMC2570062 18982106

[76] Wu G-Y, Deisseroth K, Tsien RW (2001). Spaced stimuli stabilize MAPK pathway activation and its effects on dendritic morphology. Nat. Neurosci..

[77] Thomas GM, Huganir RL (2004). MAPK cascade signalling and synaptic plasticity. Nat. Rev. Neurosci..

[78] Gallo, F. T., Katche, C., Morici, J. F., Medina, J. H. & Weisstaub, N. V. Immediate early genes, memory and psychiatric disorders: focus on c-Fos, Egr1 and Arc. *Front. Behav. Neurosci*. **12**, 79 (2018). 10.3389/fnbeh.2018.00079 PMC5932360 29755331

[79] Groenewegen, H. J., Wouterlood, F. G. & Uylings, H. B. M. Chapter 21—Organization of prefrontal-striatal connections. in *Handbook of Behavioral Neuroscience* (eds Steiner, H. & Tseng, K. Y.) Vol. 24, 423–438 (Elsevier, 2016).

[80] Hunnicutt, B. J. et al. A comprehensive excitatory input map of the striatum reveals novel functional organization. *eLife* **5**, e19103 (2016). 10.7554/eLife.19103 PMC5207773 27892854

[81] Ippolito, D. M. & Eroglu, C. Quantifying synapses: an immunocytochemistry-based assay to quantify synapse number. *J. Vis. Exp. JoVE*10.3791/2270 (2010). 10.3791/2270 PMC3159596 21113117

[82] Kim, I. H. et al. Dysregulation of the synaptic cytoskeleton in the PFC drives neural circuit pathology, leading to social dysfunction. *Cell Rep*. **32**, 107965 (2020). 10.1016/j.celrep.2020.107965 PMC8000056 32726629

[83] Anastassiadis K (2009). Dre recombinase, like Cre, is a highly efficient site-specific recombinase in *E. coli*, mammalian cells and mice. Dis. Model. Mech..

[84] Schwab ME, Javoy-Agid F, Agid Y (1978). Labeled wheat germ agglutinin (WGA) as a new, highly sensitive retrograde tracer in the rat brain hippocampal system. Brain Res..

[85] Madisen L (2010). A robust and high-throughput Cre reporting and characterization system for the whole mouse brain. Nat. Neurosci..

[86] Saunders A (2018). Molecular diversity and specializations among the cells of the adult mouse brain. Cell.

[87] Hodos W (1961). Progressive ratio as a measure of reward strength. Science.

[88] Killeen PR, Posadas-Sanchez D, Johansen EB, Thrailkill EA (2009). Progressive ratio schedules of reinforcement. J. Exp. Psychol. Anim. Behav. Process..

[89] Parker KE (2019). A paranigral VTA nociceptin circuit that constrains motivation for reward. Cell.

[90] Hershenberg R (2016). Diminished effort on a progressive ratio task in both unipolar and bipolar depression. J. Affect. Disord..

[91] Olarte-Sánchez CM, Valencia-Torres L, Cassaday HJ, Bradshaw CM, Szabadi E (2015). Quantitative analysis of performance on a progressive-ratio schedule: effects of reinforcer type, food deprivation and acute treatment with Δ9-tetrahydrocannabinol (THC). Behav. Processes.

[92] Margas, W., Ferron, L., Nieto-Rostro, M., Schwartz, A. & Dolphin, A. C. Effect of knockout of α2δ−1 on action potentials in mouse sensory neurons. *Philos. Trans. R. Soc. B Biol. Sci.* **371**, 20150430 (2016). 10.1098/rstb.2015.0430 PMC4938030 27377724

[93] Berndt A (2011). High-efficiency channelrhodopsins for fast neuronal stimulation at low light levels. Proc. Natl. Acad. Sci. USA.

[94] Owen SF, Liu MH, Kreitzer AC (2019). Thermal constraints on in vivo optogenetic manipulations. Nat. Neurosci..

[95] Alberio L (2018). A light-gated potassium channel for sustained neuronal inhibition. Nat. Methods.

[96] Kurshan PT (2018). γ-Neurexin and frizzled mediate parallel synapse assembly pathways antagonized by receptor endocytosis. Neuron.

[97] Wang T, Jones RT, Whippen JM, Davis GW (2016). α2δ-3 is required for rapid transsynaptic homeostatic signaling. Cell Rep..

[98] Cowen SL, Davis GA, Nitz DA (2012). Anterior cingulate neurons in the rat map anticipated effort and reward to their associated action sequences. J. Neurophysiol..

[99] Hillman KL, Bilkey DK (2010). Neurons in the rat anterior cingulate cortex dynamically encode cost–benefit in a spatial decision-making task. J. Neurosci..

[100] Akam, T. et al. The anterior cingulate cortex predicts future states to mediate model-based action selection. *Neuron* **109**, 149–163 (2020). 10.1016/j.neuron.2020.10.013 PMC7837117 33152266

[101] Kennerley SW, Walton ME, Behrens TEJ, Buckley MJ, Rushworth MFS (2006). Optimal decision making and the anterior cingulate cortex. Nat. Neurosci..

[102] Fouragnan EF (2019). The macaque anterior cingulate cortex translates counterfactual choice value into actual behavioral change. Nat. Neurosci..

[103] Porter, B. S., Li, K. & Hillman, K. L. Regional activity in the rat anterior cingulate cortex and insula during persistence and quitting in a physical-effort task. *eNeuro* **7**, ENEURO.0243–20.2020 (2020). 10.1523/ENEURO.0243-20.2020 PMC7545432 32859724

[104] Porter BS, Hillman KL, Bilkey DK (2019). Anterior cingulate cortex encoding of effortful behavior. J. Neurophysiol..

[105] Hayashi-Takagi A (2015). Labelling and optical erasure of synaptic memory traces in the motor cortex. Nature.

[106] Zhou J-J, Li D-P, Chen S-R, Luo Y, Pan H-L (2018). The α2δ−1–NMDA receptor coupling is essential for corticostriatal long-term potentiation and is involved in learning and memory. J. Biol. Chem..

[107] Baldwin KT, Eroglu C (2017). Molecular mechanisms of astrocyte-induced synaptogenesis. Curr. Opin. Neurobiol..

[108] Sekar A (2016). Schizophrenia risk from complex variation of complement component 4. Nature.

[109] Ullrich M (2018). OCD-like behavior is caused by dysfunction of thalamo-amygdala circuits and upregulated TrkB/ERK-MAPK signaling as a result of SPRED2 deficiency. Mol. Psychiatry.

[110] Calafate S (2015). Synaptic contacts enhance cell-to-cell tau pathology propagation. Cell Rep..

[111] Cath DC (2001). Repetitive behaviors in Tourette’s syndrome and OCD with and without tics: what are the differences?. Psychiatry Res..

[112] Luchins DJ, Goldman MB, Lieb M, Hanrahan P (1992). Repetitive behaviors in chronically institutionalized schizophrenic patients. Schizophr. Res..

[113] Bolger AM, Lohse M, Usadel B (2014). Trimmomatic: a flexible trimmer for Illumina sequence data. Bioinformatics.

[114] Langmead B, Salzberg SL (2012). Fast gapped-read alignment with Bowtie 2. Nat. Methods.

[115] Liao Y, Smyth GK, Shi W (2013). The Subread aligner: fast, accurate and scalable read mapping by seed-and-vote. Nucleic Acids Res..

[116] Liao Y, Smyth GK, Shi W (2014). featureCounts: an efficient general purpose program for assigning sequence reads to genomic features. Bioinformatics.

[117] Robinson MD, McCarthy DJ, Smyth G (2010). K. edgeR: a Bioconductor package for differential expression analysis of digital gene expression data. Bioinformatics.

[118] Yu G, Wang L-G, Han Y, He Q-Y (2012). clusterProfiler: an R package for comparing biological themes among gene clusters. OMICS J. Integr. Biol..

[119] Christopherson KS (2005). Thrombospondins are astrocyte-secreted proteins that promote CNS synaptogenesis. Cell.

[120] Kucukdereli H (2011). Control of excitatory CNS synaptogenesis by astrocyte-secreted proteins Hevin and SPARC. Proc. Natl. Acad. Sci. USA.

[121] Allen NJ (2012). Astrocyte glypicans 4 and 6 promote formation of excitatory synapses via GluA1 AMPA receptors. Nature.

[122] Risher WC (2014). Astrocytes refine cortical connectivity at dendritic spines. eLife.

[123] Singh SK (2016). Astrocytes assemble thalamocortical synapses by bridging NRX1α and NL1 via Hevin. Cell.

[124] Koh, S. et al. Thrombospondin−1 promotes circuit-specific synapse formation via β1-integrin. 10.2139/ssrn.3497027 (2019).

