# Supplementary material for: Training-induced circuit-specific excitatory synaptogenesis in mice is required for effort control
Source: Nat Commun. 2023 Sep 8;14:5522. doi: 10.1038/s41467-023-41078-z (PMC10491649; doi:10.1038/s41467-023-41078-z)
Supplement: Supplementary file 1 — Supplementary Information [file 41467_2023_41078_MOESM1_ESM.pdf]

Training-induced circuit-specific excitatory synaptogenesis in mice is required for effort control

*#Francesco Paolo Ulloa Severino[1-2-3], #Oluwadamilola O. Lawal[4], Kristina Sakers[1], Shiyi Wang[1], Namsoo Kim[2], Alexander Friedman[2], Sarah Johnson[1], Chaichontat Sriworarat[1], Ryan Hughes[2], Scott H. Soderling[1-4-5], Il Hwan Kim[6], *Henry H. Yin[2-4-5], *Cagla Eroglu[1-4-5-7]

[1]Department of Cell Biology, Duke University Medical Center, Durham, NC 27710, USA.
[2]Department of Psychology and Neuroscience, Duke University, Durham, NC, 27710, USA.
[3]Present Address: Cajal Institute (CSIC), Madrid, 28001, Spain.
[4]Department of Neurobiology, Duke University Medical Center, Durham, NC 27710, USA.
[5]Duke Institute for Brain Sciences (DIBS), Durham, NC, 27710, USA.
[6]Department of Anatomy & Neurobiology, University of Tennessee Health and Science Center, Memphis, TN 38103, USA.
[7]Howard Hughes Medical Institute, Duke University, Durham, NC 27710, USA.

#These authors contributed equally.

*Corresponding authors' e-mail addresses:

francesco.ulloa@cajal.csic.es

hy43@duke.edu

cagla.eroglu@duke.edu

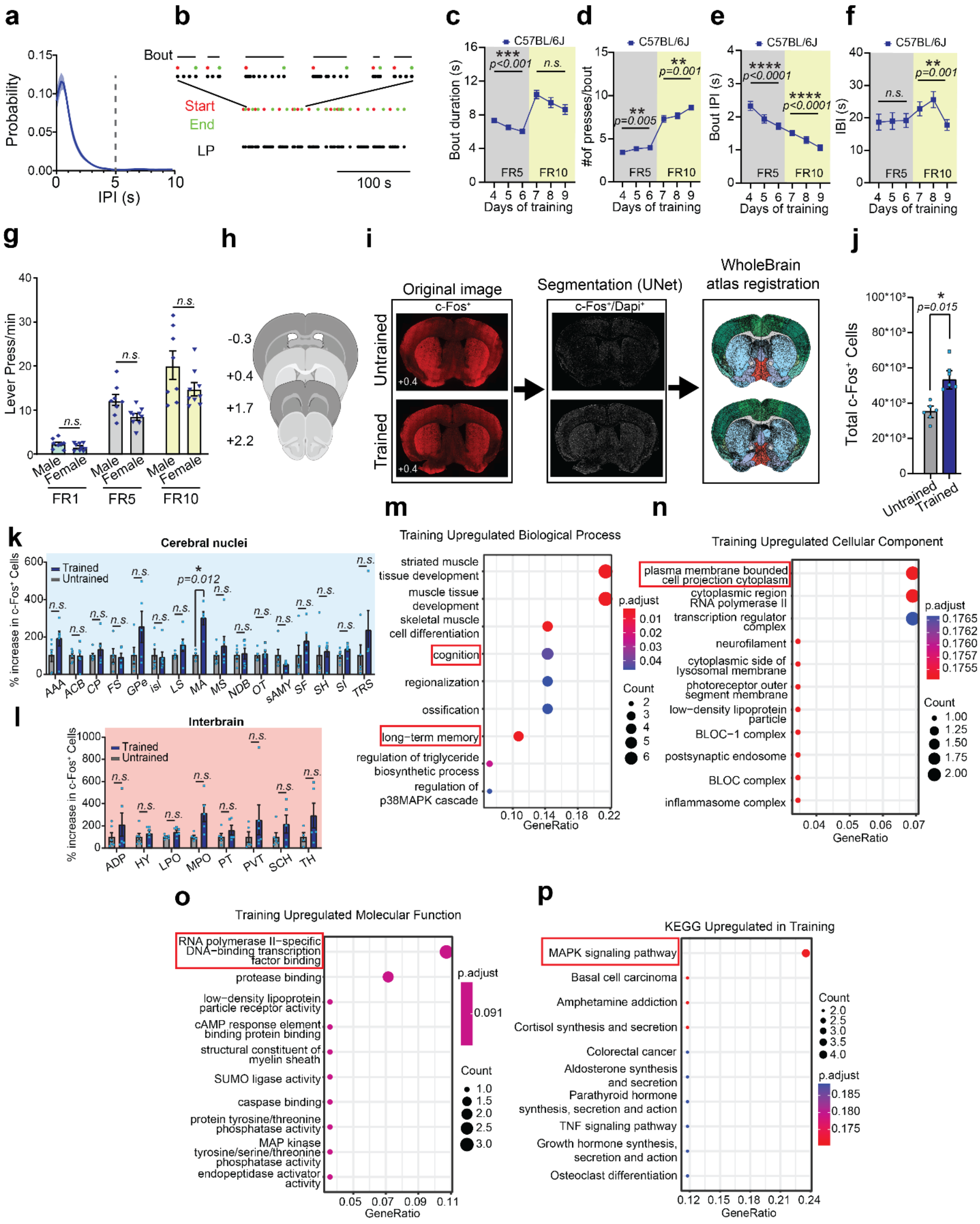

a
Probability
0.15
0.10
0.05
0.00
0
5
10
IPI (s)
b
Bout
Start
End
LP
100 s
c
C57BL/6J
***
p<0.001
n.s.
Bout duration (s)
FR5
FR10
Days of training
d
C57BL/6J
**
p=0.005
**
p=0.001
#of presses/bout
FR5
FR10
Days of training
e
C57BL/6J
****
p<0.0001
****
p<0.0001
Bout IPI (s)
FR5
FR10
Days of training
f
C57BL/6J
n.s.
**
p=0.001
IBI (s)
FR5
FR10
Days of training
g
Lever Press/min
n.s.
Male
Female
FR1
FR5
FR10
h
-0.3
+0.4
+1.7
+2.2
i
Original image
c-Fos⁺
Segmentation (UNet)
c-Fos⁺/Dapi⁺
WholeBrain
atlas registration
Untrained
Trained
+0.4
j
Total c-Fos⁺ Cells
*
p=0.015
100*10³
80*10³
60*10³
40*10³
20*10³
Untrained
Trained
k
Cerebral nuclei
Trained
Untrained
% increase in c-Fos⁺ Cells
n.s.
*
p=0.012
AAA
ACB
CP
FS
GPe
isl
LS
MA
MS
NDB
OT
sAMY
SF
SH
SI
TRS
l
Interbrain
Trained
Untrained
% increase in c-Fos⁺ Cells
n.s.
ADP
HY
LPO
MPO
PT
PVT
SCH
TH
m
Training Upregulated Biological Process
striated muscle tissue development
muscle tissue development
skeletal muscle cell differentiation
cognition
regionalization
ossification
long−term memory
regulation of triglyceride biosynthetic process
regulation of p38MAPK cascade
GeneRatio
p.adjust
Count
n
Training Upregulated Cellular Component
plasma membrane bounded cell projection cytoplasm
cytoplasmic region
RNA polymerase II transcription regulator complex
neurofilament
cytoplasmic side of lysosomal membrane
photoreceptor outer segment membrane
low−density lipoprotein particle
BLOC−1 complex
postsynaptic endosome
BLOC complex
inflammasome complex
GeneRatio
p.adjust
Count
o
Training Upregulated Molecular Function
RNA polymerase II−specific DNA−binding transcription factor binding
protease binding
low−density lipoprotein particle receptor activity
cAMP response element binding protein binding
structural constituent of myelin sheath
SUMO ligase activity
caspase binding
protein tyrosine/threonine phosphatase activity
MAP kinase tyrosine/serine/threonine phosphatase activity
endopeptidase activator activity
GeneRatio
p.adjust
Count
p
KEGG Upregulated in Training
MAPK signaling pathway
Basal cell carcinoma
Amphetamine addiction
Cortisol synthesis and secretion
Colorectal cancer
Aldosterone synthesis and secretion
Parathyroid hormone synthesis, secretion and action
TNF signaling pathway
Growth hormone synthesis, secretion and action
Osteoclast differentiation
GeneRatio
Count
p.adjust

Supplementary Figure 1. Brain-wide and ACC-specific screening of IEG expression in trained and untrained mice

a, Probability of inter-press interval (IPI) utilized to identify lever press bouts. **b**, Example of identified Start and End of the lever press bouts within a behavioral session. **c**, Bout Duration (*RM one-way ANOVA*. FR5 *[F (1.82, 29.1) = 16.3], p < 0.0001*; FR10 *[F (1.32, 21.0) = 5.71], p = 0.019*). *Holm-Sidak`s multiple comparisons adjusted p-value reported in figure*. **d**, Number of presses per bout (*RM one-way ANOVA*. FR5 *[F (1.98, 31.6) = 7.86], p = 0.001*; FR10 *[F (1.96, 31.3) = 11.7], p < 0.001*). *Holm-Sidak`s multiple comparisons adjusted p-value reported in figure*. **e**, Bout IPI (*RM one-way ANOVA.* FR5 *[F (1.73, 27.7) = 36.0], p < 0.0001*; FR10 *[F (1.78, 28.4) = 21.8], p < 0.0001*). *Holm-Sidak`s multiple comparisons adjusted p-value reported in figure*. **f**, Inter Bout Interval (*RM one-way ANOVA [F (2.32, 37.1) = 3.22], p=0.045*). *Holm-Sidak`s multiple comparisons adjusted p-value reported in figure*. For graphs in c-f: (n=17 mice). **g**, Quantification of lever press/min in male (n = 8) versus female (n = 9) mice. *RM Two-way ANOVA, main effect of ratio [F (1.142, 17.13) = 112.3, p<0.0001]* and no effect of sex *[F (1,15) = 4.155, p=0.059]* nor interaction *[F (2,30) = 2.442, p=0.104]*. **h**, Stereotaxic coordinates of the brain sections used for the c-Fos analysis (created with BioRender.com). **i**, Image processing flow chart with examples for a trained and an untrained animal. **j**, Total numbers of c-Fos$^+$ cells in Untrained (35.1 ± 3.2 $*10^3$ c-Fos$^+$ cells) and Trained mice (53.2 ± 5.1 $*10^3$ c-Fos$^+$ cells). n = 6 mice per condition; mean of 4 sections per mouse. *Unpaired two-tailed Mann-Whitney test [U = 3]*. **k**, Bar plot of c-Fos$^+$ cells for the cerebral nuclei regions. *n = 6 mice per condition, except for the following regions due to their absence in some cases:* FS, Gpe, MA, sAMY, SF (n=5 mice per condition), TRS (n=5 mice Untrained; n=4 mice trained). *Multiple unpaired t-test with Welch correction. Multiple comparisons using Holm-Sidak method; alpha = 0.05 for adjusted p-value*. MA (300.54 ± 35.11%, *[t (6.26) = 6.0]*). n = 4-6 mice per condition. **l**, Bar plot of c-Fos$^+$ cells for the Interbrain regions. *n = 6 mice per condition, except for the following regions due to their absence in some cases:* ADP (n=6 untrained; n=5 trained), MPO and PT (n=5 untrained; n=5 trained), PVT (n=5 untrained; n=6 trained), SCH (n=6 untrained; n=5 trained); TH (n=4 untrained; n=5 trained). *Multiple unpaired t-test with Welch correction. Multiple comparisons using Holm-Sidak method; alpha = 0.05 for adjusted p-value*. n = 4-6 mice per condition. **m-p**, Gene Ontology and KEGG pathway analysis for the overexpressed DEG. For all graphs: Data shown as mean ± s.e.m. alpha = 0.05. Source data are provided as a Source Data file.

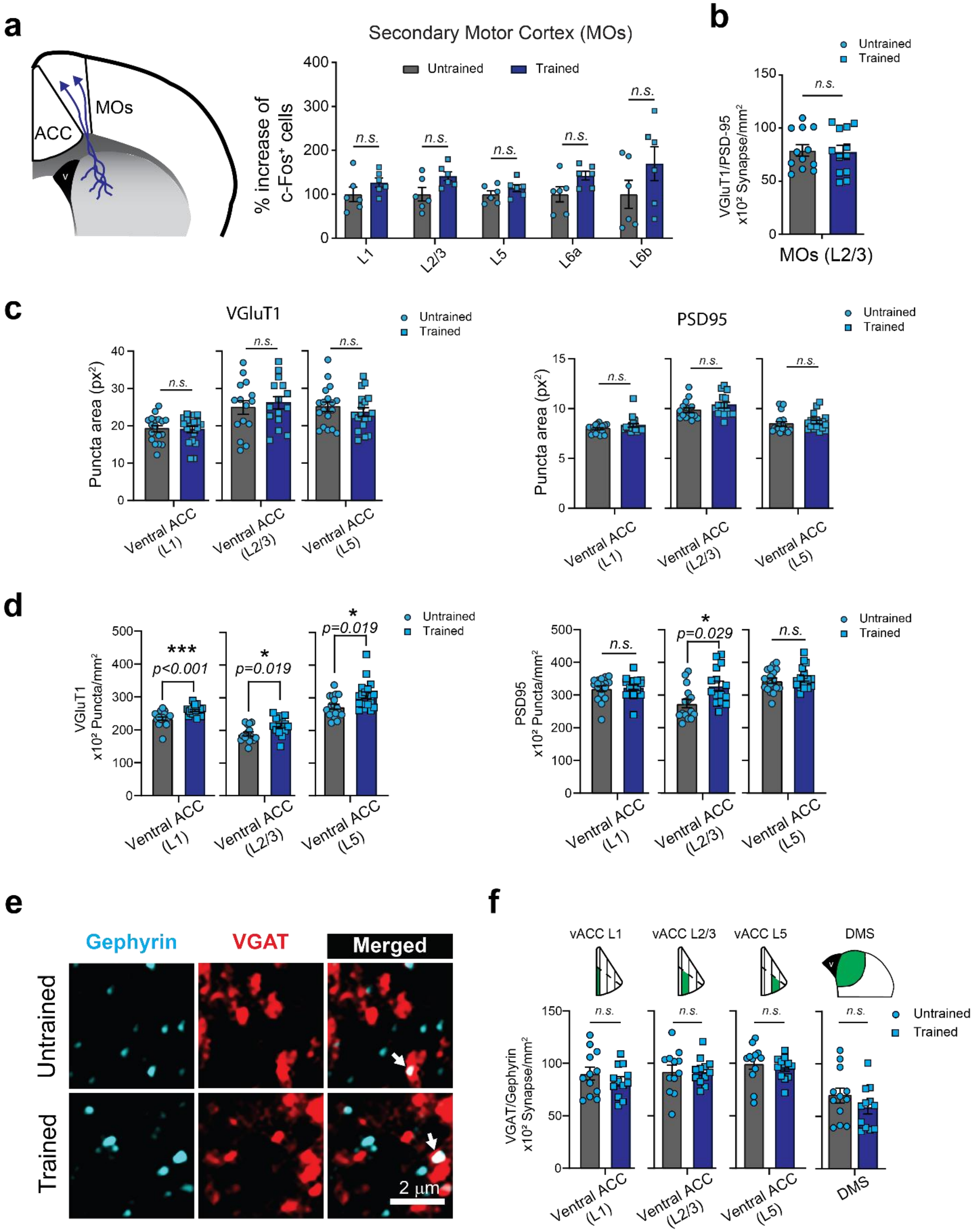
a
ACC
MOs
Secondary Motor Cortex (MOs)
Untrained
Trained
% increase of c-Fos+ cells
n.s.
L1
L2/3
L5
L6a
L6b
b
VGluT1/PSD-95 x10² Synapse/mm²
MOs (L2/3)
c
VGluT1
PSD95
Puncta area (px²)
Ventral ACC (L1)
Ventral ACC (L2/3)
Ventral ACC (L5)
d
VGluT1 x10² Puncta/mm²
PSD95 x10² Puncta/mm²
***
p<0.001
*
p=0.019
*
p=0.019
*
p=0.029
e
Gephyrin
VGAT
Merged
Untrained
Trained
2 µm
f
vACC L1
vACC L2/3
vACC L5
DMS
VGAT/Gephyrin x10² Synapse/mm²

Supplementary Figure 2. Training induces excitatory but not inhibitory synaptogenesis in the ACC. **a**, *Left.* Schematic representation of close proximity to the ACC of L2/3 neurons projecting from the MOs to the DMS. *Right.* Layer-specific count of c-Fos⁺ cells in the MOs. (n=6 mice per condition). *Multiple unpaired t-test with Welch correction. Multiple comparisons using Holm-Sidak method; alpha = 0.05 for adjusted p-value*. **b**, Quantification of VGlut1/PSD95 co-localized puncta in layer 2/3 of MOs (n = 4 mice per condition, 3 images per mouse). *Unpaired Two-tailed t-test with Welch`s correction [t (22) = 0.17].* **c**, Quantification of synaptic puncta area for VGluT1 and PSD95 in Untrained and Trained mice. For L1 and L5 (n = 6 mice per condition, 3 images per mouse); for L2/3 (n=5 mice per condition, 3 images per mouse). *Multiple unpaired t-test with Welch correction. Multiple comparisons using Holm-Sidak method; alpha = 0.05 for adjusted p-value*. L1 Untrained VGluT1 (19 ± 0.83), Trained VGluT1 (19 ± 0.88), *[t (34) = 0.17]*; PSD95 Untrained (8.0 ± 0.1), Trained (8.3 ± 0.19), *[t (26) = 1.5]*; L2/3 Untrained VGluT1 (25 ± 1.8), Trained VGluT1 (26 ± 1.6), *[t (27.7) = 0.5]*; PSD95 Untrained (9.8 ± 0.24), Trained (10 ± 0.29), *[t (27.2) = 1.35]*; L5 Untrained VGluT1 (25 ± 1.3), Trained VGluT1 (24 ± 1.2), *[t (33.8) = 0.84]*; PSD95 Untrained (8.5 ± 0.22), Trained (8.6 ± 0.19), *[t (33.5) = 0.59]*. **d**, Quantification of individual VGluT1 or PSD95 puncta in the ACC of untrained and trained WT mice. For L1 and L5 (n = 6 mice per condition, 3 images per mouse); for L2/3 (n=5 mice per condition, 3 images per mouse). *Multiple unpaired t-test with Welch correction. Multiple comparisons using Holm-Sidak method; alpha = 0.05 for adjusted p-value.* VGluT1: L1 Untrained (234 ± 4.92), Trained (259 ± 3.35) [t (29.9) = 4.08]; L2/3 Untrained (187 ± 6.23), Trained (213 ± 7.11) [t (27.5) = 2.67]; L5 Untrained (270 ± 7.52), Trained (305 ± 10.2) [t (31.3) = 2.75]; PSD95: L1 Untrained (320 ± 7.97), Trained (324 ± 7.18) [t (33.6) = 0.34]; L2/3 Untrained (274 ± 13.1), Trained (328 ± 14.4) [t (27.7) = 2.8]; L5 Untrained (344 ± 8.13), Trained (351 ± 8.10) [t (34) = 0.63]. **e**, Representative images for Untrained and Trained mice of Gephyrin and VGAT staining. The *arrows* in the merged channel indicate co-localized puncta. *Scale bar 20 µm*. **f**, Quantification of Gephyrin/VGAT co-localized puncta in ACC (L1, L2/3 and L5). *Multiple unpaired t-test with Welch correction. Multiple comparisons using Holm-Sidak method; alpha = 0.05 for adjusted p-value*. L1 Untrained (90 ± 6.1), Trained (83 ± 4.3) *[t (19.7) = 0.99]*; L2/3 Untrained (92 ± 6.0), Trained (92 ± 3.7) *[t (18.2) = 0.00028]*; L5 Untrained (100 ± 5.4), Trained (96 ± 3.2) [t (17.8) = 0.67]; and DMS (Untrained (70 ± 7.1), Trained (58 ± 5.8), *[t (21) = 1.3].* n = 4 mice per condition, 3 images per mouse. For all graphs: Data shown as mean ± s.e.m. alpha = 0.05. Source data are provided as a Source Data file.

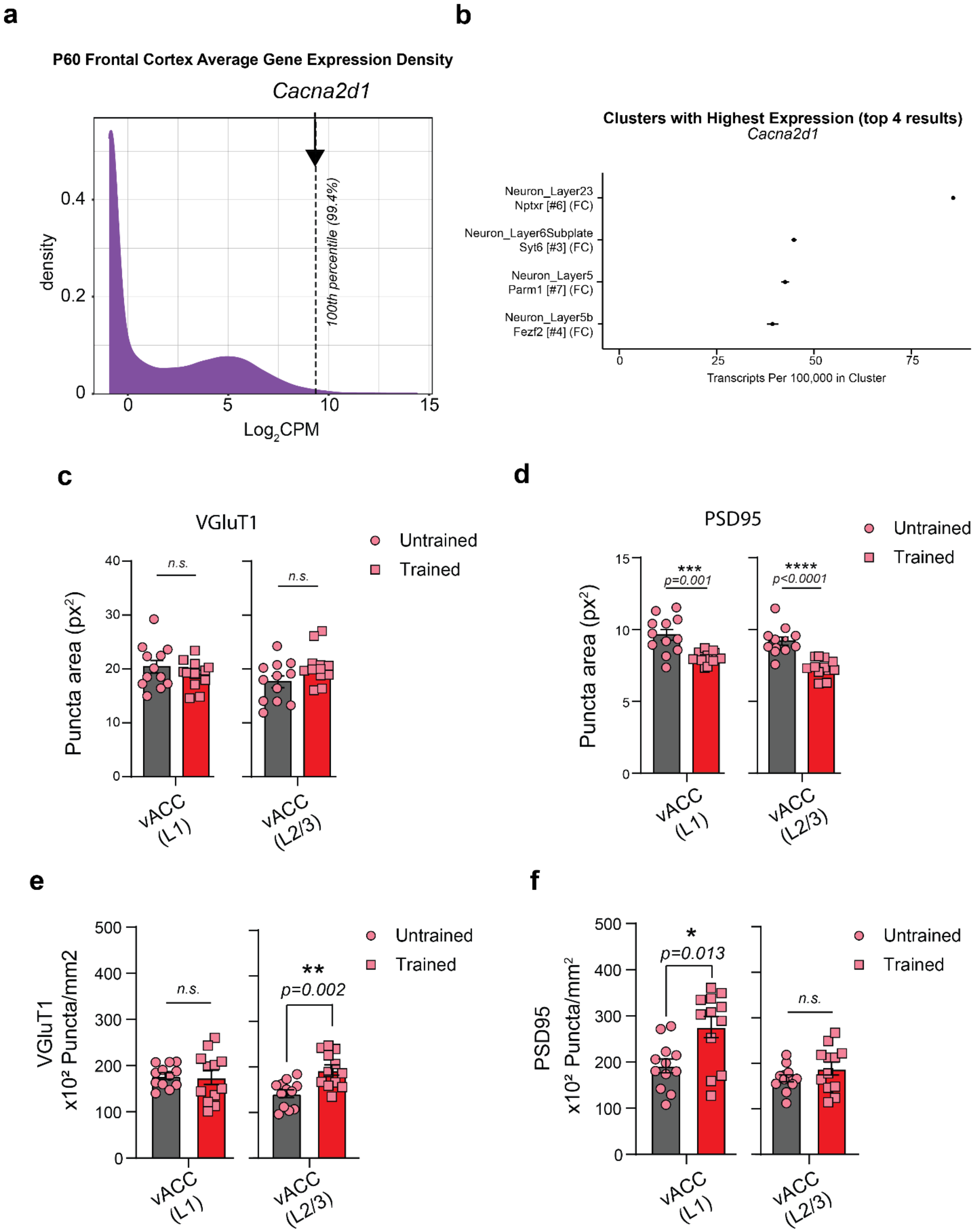

a
P60 Frontal Cortex Average Gene Expression Density
Cacna2d1
density
100th percentile (99.4%)
Log2CPM
b
Clusters with Highest Expression (top 4 results)
Cacna2d1
Neuron_Layer23 Nptxr [#6] (FC)
Neuron_Layer6Subplate Syt6 [#3] (FC)
Neuron_Layer5 Parm1 [#7] (FC)
Neuron_Layer5b Fezf2 [#4] (FC)
Transcripts Per 100,000 in Cluster
c
VGluT1
Untrained
Trained
Puncta area (px2)
n.s.
n.s.
vACC (L1)
vACC (L2/3)
d
PSD95
Untrained
Trained
Puncta area (px2)
***
p=0.001
****
p<0.0001
vACC (L1)
vACC (L2/3)
e
VGluT1 x10² Puncta/mm2
Untrained
Trained
n.s.
**
p=0.002
vACC (L1)
vACC (L2/3)
f
PSD95 x10² Puncta/mm2
Untrained
Trained
*
p=0.013
n.s.
vACC (L1)
vACC (L2/3)

Supplementary Figure 3. *CACNA2D1* is highly expressed in layer 2/3 of the frontal cortex.
a, Expression density (log_2CPM) of *Cacna2d1* gene (encoding for α2δ-1) in the prefrontal cortex of adult mice (P60). Data mined from Saunders et al., 2018. **b**, Cacna2d1 expression level across layers of the prefrontal cortex. Data mined from Saunders et al., 2018. **c**, Quantification of synaptic puncta area for VGluT1 in Untrained and Trained α2δ-1 KO mice. L1 Untrained VGluT1 (20 ± 1.2), Trained VGluT1 (19 ± 0.75), *[t (18.7) = 0.98]*; L2/3 Untrained VGluT1 (18 ± 1.1), Trained VGluT1 (20 ± 0.95), *[t (21.6) = 1.9]*. **d**, Quantification of synaptic puncta area for PSD95 in Untrained and Trained α2δ-1 KO mice. L1 Untrained PSD95 (9.6 ± 0.37), Trained PSD95 (8.0 ± 0.12), *[t (13.4) = 4.1]*; L2/3 Untrained PSD95 (9.2 ± 0.28), Trained PSD95 (7.3 ± 0.18), *[t (18.9) = 5.5].* **e**, Quantification of VGluT1 puncta alone in untrained vs trained α2δ-1 KO mice. L1 Untrained VGluT1 (178 ± 6.9), Trained VGluT1 (174 ± 15), *[t (22) = 0.19]*; L2/3 Untrained VGluT1 (139 ± 8.4), Trained VGluT1 (190 ± 11), *[t (22) = 3.7]*. **f**, Quantification of PSD95 puncta alone in Untrained and Trained α2δ-1 KO mice. *Unpaired Two-tailed t-test.* L1 Untrained PSD95 (192 ± 15), Trained PSD95 (276 ± 23), *[t (18.8) = 3.0]*; L2/3 Untrained PSD95 (165 ± 7.8), Trained PSD95 (186 ± 14), *[t (17.3) = 1.3]. For graphs c-f n=4 mice per condition, 3 sections per mouse. For all graphs: Multiple unpaired t-test with Welch correction. Multiple comparisons using Holm-Sidak method; alpha = 0.05 for adjusted p-value.* Data shown as mean ± s.e.m. Source data are provided as a Source Data file.

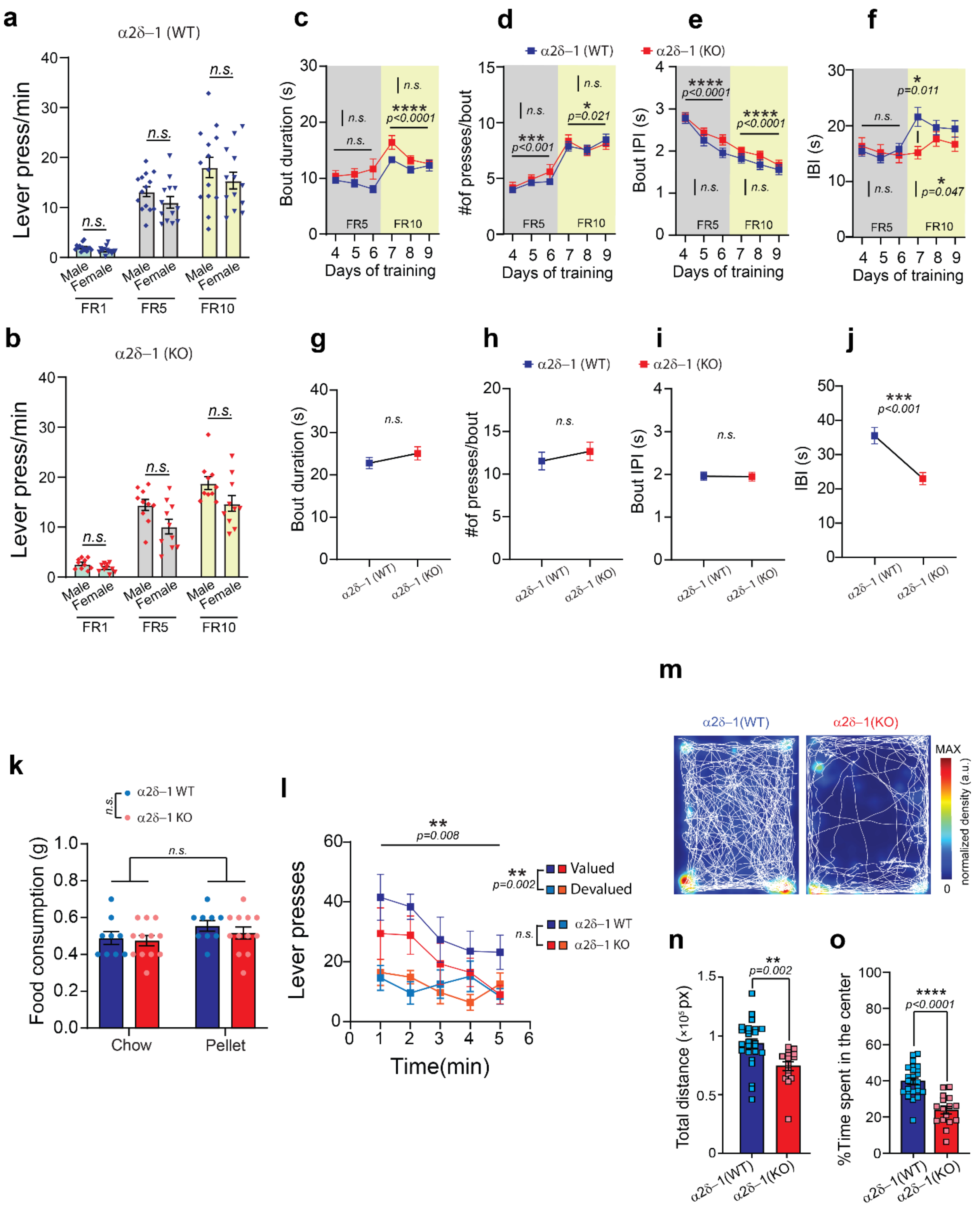

Supplementary Figure 4. α2δ-1 KO mice cannot effectively control effort exertion during a demanding task. a, Quantification of lever press/min in α2δ-1 WT male (n = 14) versus female (n = 13)

mice. α2δ-1 WT: *RM Two-way ANOVA, main effect of ratio [F (1.31, 32.75) = 126.6, p < 0.0001] and no effect of sex [F (1, 25) = 1.6, p = 0.217] nor interaction [F (2, 50) = 0.6066, p = 0.549].* **b**, Quantification of lever press/min in α2δ-1 KO male (n = 10) versus female (n = 10) mice. *α2δ-1 KO: RM Two-way ANOVA, main effect of ratio [F (1.707, 30.73) = 191.4, p < 0.0001], main effect of sex [F (1, 18) = 5.317, p = 0.033] and interaction [F (2, 36) = 3.285, p = 0.048]. Multiple comparisons using Holm-Sidak method; alpha = 0.05 for adjusted p-value showed no sexual dimorphism, CRF [t (17.26) = 1.97, p = 0.18], FR5 [t (16.68) = 2.37, p = 0.088], FR10 [t (17.16) = 1.99, p = 0.174].* **c**, Bout Duration (*RM Two-way ANOVA*. FR5: No *effect of Days [F (1.308, 58.87) = 0.03069, p = 0.915] nor Genotype [F (1, 45) = 3.407, p = 0.071] and no interaction [F (2, 90) = 2.435, p = 0.093]).* FR10: M*ain effect of Days [F (1.778, 79.99) = 15.93], no effect of Genotype [F (1, 45) = 2.796, p = 0.101] and interaction [F (2, 90) = 3.810, p = 0.026], Multiple comparison showed no effect between genotypes).* **d**, Number of presses per bout (*RM Two-way ANOVA*. FR5: M*ain effect of Days [F (1.324, 59.57) = 10.18] no effect of Genotype [F (1, 45) = 1.026, p = 0.316] and no interaction [F (2, 90) = 1.181, p = 0.312])*. FR10: *Main effect of Days [F (1.729, 77.82) = 4.322] no effect of Genotype [F (1, 45) = 8.166e-005, p = 0.993] and no interaction [F (2, 90) = 1.180, p = 0.312])*. **e**, Bout IPI *(RM Two-way ANOVA*. FR5: M*ain effect of Days [F (1.559, 70.16) = 116.8] no effect of Genotype [F (1, 45) = 1.031, p = 0.315] and significant interaction [F (2, 90) = 4.791, p = 0.010]. Multiple comparison showed no differences between genotypes)*. FR10: M*ain effect of Days [F (1.637, 73.65) = 39.08] no effect of Genotype [F (1, 45) = 1.103, p = 0.299] and no interaction [F (2, 90) = 1.209, p = 0.303])*. **f**, Inter Bout Interval *(RM Two-way ANOVA*. FR5: No *effect of Days [F (1.706, 76.76) = 2.279, p = 0.117] no effect of Genotype [F (1, 45) = 0.03223, p = 0.858] and no interaction [F (2, 90) = 2.044, p = 0.135]). FR10: No effect of Days [F (1.488, 66.95) = 0.2467, p = 0.715], main effect of Genotype [F (1, 45) = 4.166, p = 0.047] and significant interaction [F (2, 90) = 3.916, p = 0.023]).* For graphs in c-f: WT (n=27 mice), KO (n=20 mice). **g**, Bout Duration in α2δ-1 WT (23 ± 1.3) and KO (25 ± 1.6), *Unpaired Two-tailed t-test. [t (39) = 1.1, p = 0.266]*. **h**, Number of presses per bout in α2δ-1 WT (12 ± 1.0) and KO (13 ± 1.1), *Unpaired Two-tailed t-test. [t (39) = 0.77, p = 0.448])*. **i**, Bout IPI in α2δ-1 WT (2 ± 0.09) and KO (1.9 ± 0.10), *Unpaired Two-tailed t-test. [t (39) = 0.079, p = 0.937]*. **j**, Inter Bout Interval in α2δ-1 WT (36 ± 2.4) and KO (23 ± 1.8), *Unpaired Two-tailed t-test. [t (39) = 4.1, p<0.001]*. For graphs in g-j: WT (n=22 mice), KO (n=19 mice). **k**, Hunger test. WT (n=9 mice), KO (n=12 mice). (RM Two-way ANOVA. No effect of Genotype [F (1, 19) = 0.661, p = 0.426], no effect of food type [F (1, 19) = 3.106, p = 0.094], no interaction [F (1, 19) = 0.165, p = 0.6888]. **l**, Devaluation test plot over time. WT (n=10 mice), KO (n=7 mice). RM Three-way ANOVA. Significant effect of time [F (4, 60) = 3.78], a significant effect of value state [F (0.67, 10.03) = 20.34], no effect of genotype [F (1, 15) = 3.78, p = 0.071] and no interaction [F (1,15) = 2.80, p = 0.115].**m**, Examples of open field patterns of locomotion by α2δ-1 WT and KO mice. **n**, Bar graph of the total distance travelled in pixels for α2δ-1 WT (n = 25; $9.3*10^4 \pm 4.0*10^3$ pixels) and KO (n = 16; $7.4*10^4 \pm 3.8*10^3$ pixels) mice during the open field test. *Unpaired Two-tailed t-test [t (39) = 3.2].* **o**, Percent time spent in the center of the arena by α2δ-1 WT (n = 25; 40 ± 1.7%) and KO (n = 16; 24 ± 2.1%) mice. *Unpaired Two-tailed t-test [t (39) = 5.9].* For all graphs: Data shown as mean ± s.e.m. alpha = 0.05. For all the graphs: Data shown as mean ± s.e.m. *Multiple comparisons using Holm-Sidak method; alpha = 0.05 for adjusted p-value.* Source data are provided as a Source Data file.

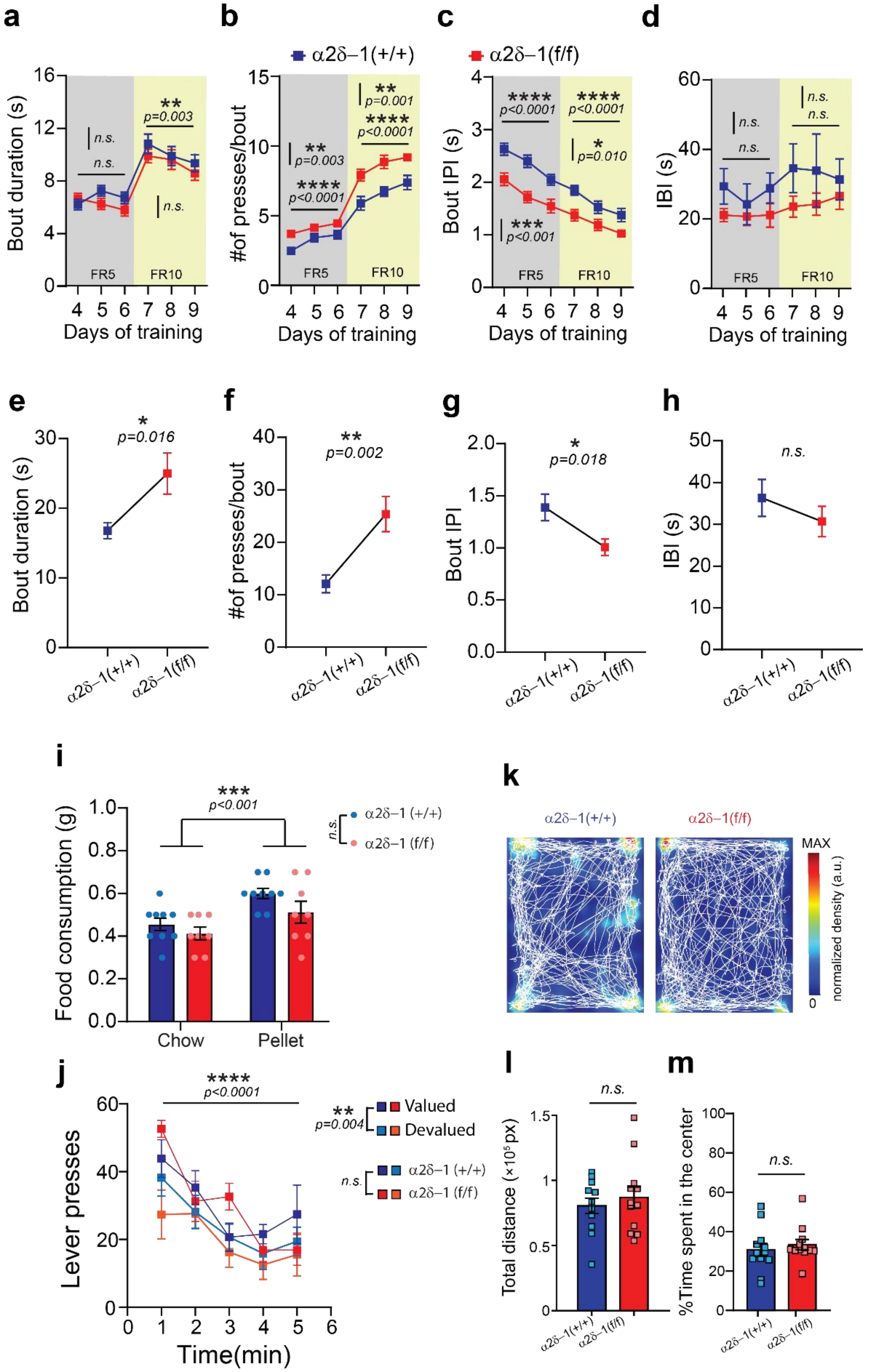
a
b
c
d
α2δ−1(+/+)
α2δ−1(f/f)
Bout duration (s)
#of presses/bout
Bout IPI (s)
IBI (s)
FR5
FR10
Days of training
n.s.
**
p=0.003
p=0.001
****
p<0.0001
***
p<0.001
*
p=0.010
e
f
g
h
p=0.016
p=0.002
p=0.018
Bout IPI
α2δ−1(+/+)
α2δ−1(f/f)
i
Food consumption (g)
Chow
Pellet
α2δ−1 (+/+)
α2δ−1 (f/f)
j
Lever presses
Time(min)
p=0.004
Valued
Devalued
k
MAX
normalized density (a.u.)
l
Total distance (×10⁵ px)
m
%Time spent in the center

Supplementary Figure 5. Circuit-specific conditional deletion of α2δ-1 alters bout properties resulting in increased effort exertion.

a, Bout Duration (*RM Two-way ANOVA*. FR5: *no effect of Days [F (1.348, 29.66) = 1.629, p = 0.215] nor Genotype [F (1, 22) = 0.9778, p = 0.333] and significant interaction [F (2, 44) = 4.105, p = 0.023]). Multiple comparison showed no differences between genotypes.* FR10: *Main effect of Days [F (1.760, 38.72) = 7.238], no effect of Genotype [F (1, 22) = 0.6237, p = 0.438] and no interaction [F (2, 44) = 0.4177, p = 0.661])*. **b**, Number of presses per bout (*RM Two-way ANOVA*. FR5: *Main effect of Days [F (1.809, 39.81) = 21.29] and Genotype [F (1, 22) = 11.04] and no interaction [F (2, 44) = 1.563, p = 0.221].* FR10: *Main effect of Days [F (1.969, 43.32) = 19.06] and Genotype [F (1, 22) = 13.08] and no interaction [F (2, 44) = 0.2286, p = 0.796]).* **c**, Bout IPI *(RM Two-way ANOVA*. FR5: *Main effect of Days [F (1.543, 33.95) = 34.17] and of Genotype [F (1, 22) = 16.07] and no significant interaction [F (2, 44) = 0.9428, p = 0.397].* FR10: *Main effect of Days [F (1.956, 43.04) = 38.95] and Genotype [F (1, 22) = 7.784] and no interaction [F (2, 44) = 1.322, p = 0.277]).* **d**, Inter Bout Interval *(RM Two-way ANOVA*. FR5: N*o effect of Days [F (1.769, 38.92) = 0.5621, p = 0.553] no effect of Genotype [F (1, 22) = 1.810, p = 0.192] and no interaction [F (2, 44) = 0.4030, p = 0.671].* FR10: *No effect of Days [F (1.804, 39.70) = 0.001295, p = 0.997], no effect of Genotype [F (1, 22) = 1.130, p = 0.299] and no interaction [F (2, 44) = 0.5863, p = 0.561]). For the graphs in a-d: n = 12 mice per condition.* **e**, Bout Duration in α2δ-1 (+/+) (17 ± 1.2) and α2δ-1 (f/f) (25 ± 2.9), *Unpaired Two-tailed t-test. [t (22) = 2.6,]*. **f**, Number of presses per bout in α2δ-1 (+/+) (12 ± 1.7) and α2δ-1 (f/f) (25 ± 3.4), *Unpaired Two-tailed t-test. [t (22) = 3.5]*. **g**, Bout IPI in α2δ-1 (+/+) (1.4 ± 0.13) and α2δ-1 (f/f) (1.0 ± 0.08), *Unpaired Two-tailed t-test. [t (22) = 2.5]*. **h**, Inter Bout Interval in α2δ-1 (+/+) (36 ± 4.4) and α2δ-1 (f/f) (31 ± 3.6), *Unpaired Two-tailed t-test. [t (22) = 0.99, p = 0.335]*. For all the graphs: Data shown as mean ± s.e.m. *Multiple comparisons using Holm-Sidak method; alpha = 0.05 for adjusted p-value. For the graphs in e-h: n = 12 mice per condition.* **i**, Hunger test. α2δ-1 (+/+) (n = 9) and α2δ-1 (f/f) (n = 8). (RM Two-way ANOVA. No effect of Genotype [F (1, 15) = 2.477, p = 0.136], an effect of food type [F (1, 15) = 23.40, p < 0.001], no interaction [F (1, 15) = 0.773, p = 0.393]. **j**, Devaluation test plot over time. α2δ-1 (+/+) (n=9 mice); α2δ-1 (f/f) (n=8 mice). RM Three-way ANOVA. Significant effect of time [F (4, 60) = 14.4, p < 0.0001], a significant effect of value state [F (0.62, 9.31) = 18.87, p = 0.004], no effect of genotype [F (1, 15) = 0.732, p = 0.405] and no interaction [F (1,15) = 1.87, p = 0.191]. **k**, Examples of open field patterns of locomotion by α2δ-1 (+/+) and α2δ-1 (f/f) mice. **l**, Bar graph of the total distance traveled in pixels for α2δ-1 (+/+) (n = 12; $8.0*10^4 \pm 5.8*10^3$ pixels) and α2δ-1(f/f) (n = 12; $8.7*10^4 \pm 8.2*10^3$ pixels) mice during the open field test. *Unpaired Two-tailed t-test [t (22) = 0.62, p = 0.538]*. **m**, Percent time spent in the center of the arena by α2δ-1 (+/+) (n = 12; 31 ± 3.3%) and α2δ-1(f/f) (n = 12; 33 ± 2.6%) mice. *Unpaired Two-tailed t-test [t (22) = 0.64, p = 0.526].* For all graphs: *Multiple comparisons using Holm-Sidak method; alpha = 0.05 for adjusted p-value*. Source data are provided as a Source Data file.

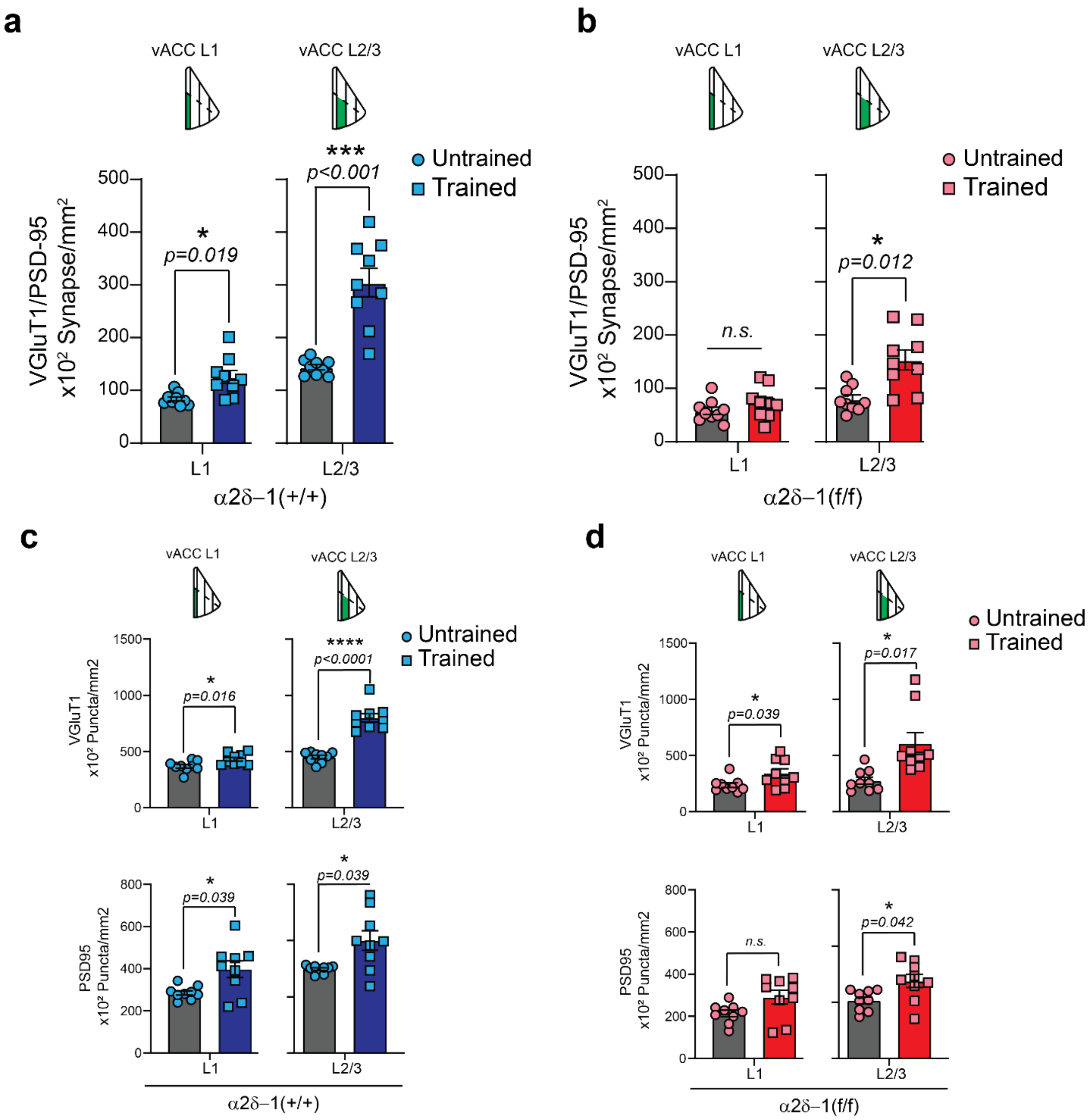

Supplementary Figure 6. Training-induced excitatory synaptogenesis onto ACC$_{\rightarrow DMS}$ neurons is reduced in cKO mice.

a, Quantification of VGlut1/PSD95 co-localized puncta in L1 and L2/3 of vACC for untrained and trained α2δ-1 (+/+) (n = 3 mice per condition, 3 images per mouse). L1 *[t (9.6) = 3.2];* L2/3 *[t (8.5) = 5.8].* **b**, Quantification of VGlut1/PSD95 co-localized puncta in L1 and L2/3 of vACC for untrained and trained α2δ-1 (f/f) (n = 3 mice per condition, 3 images per mouse). L1 *[t (14.14) = 1.2, p = 0.408];* L2/3 *[t (10.79) = 3.6].* **c**, Quantification of VGluT1 and PSD95 puncta alone in untrained and trained α2δ-1 (+/+) (n = 3 mice per condition, 3 images per mouse).VGluT1: L1, *[t (15.97) = 2.69];* L2/3 *[t (10.75) = 8.60]; PSD95:* L1, *[t (9.11) = 2.73];* L2/3 *[t (8.27) = 2.88].* **d**, Quantification of VGluT1 and PSD95 puncta alone in untrained and trained α2δ-1 (f/f) (n = 3 mice per condition, 3 images per mouse). VGluT1: L1, *[t (11.79) = 2.31];* L2/3 *[t (9.74) = 3.26]; PSD95:* L1, *[t (11.37) = 2.13];* L2/3 *[t (12.10) = 2.64].* For all the graphs: *Multiple unpaired Two-tailed t-test with Welch`s correction, multiple comparison with Holm-Sidak method alpha = 0.05 for adjusted p-value.* Data shown as mean ± s.e.m. Source data are provided as a Source Data file.

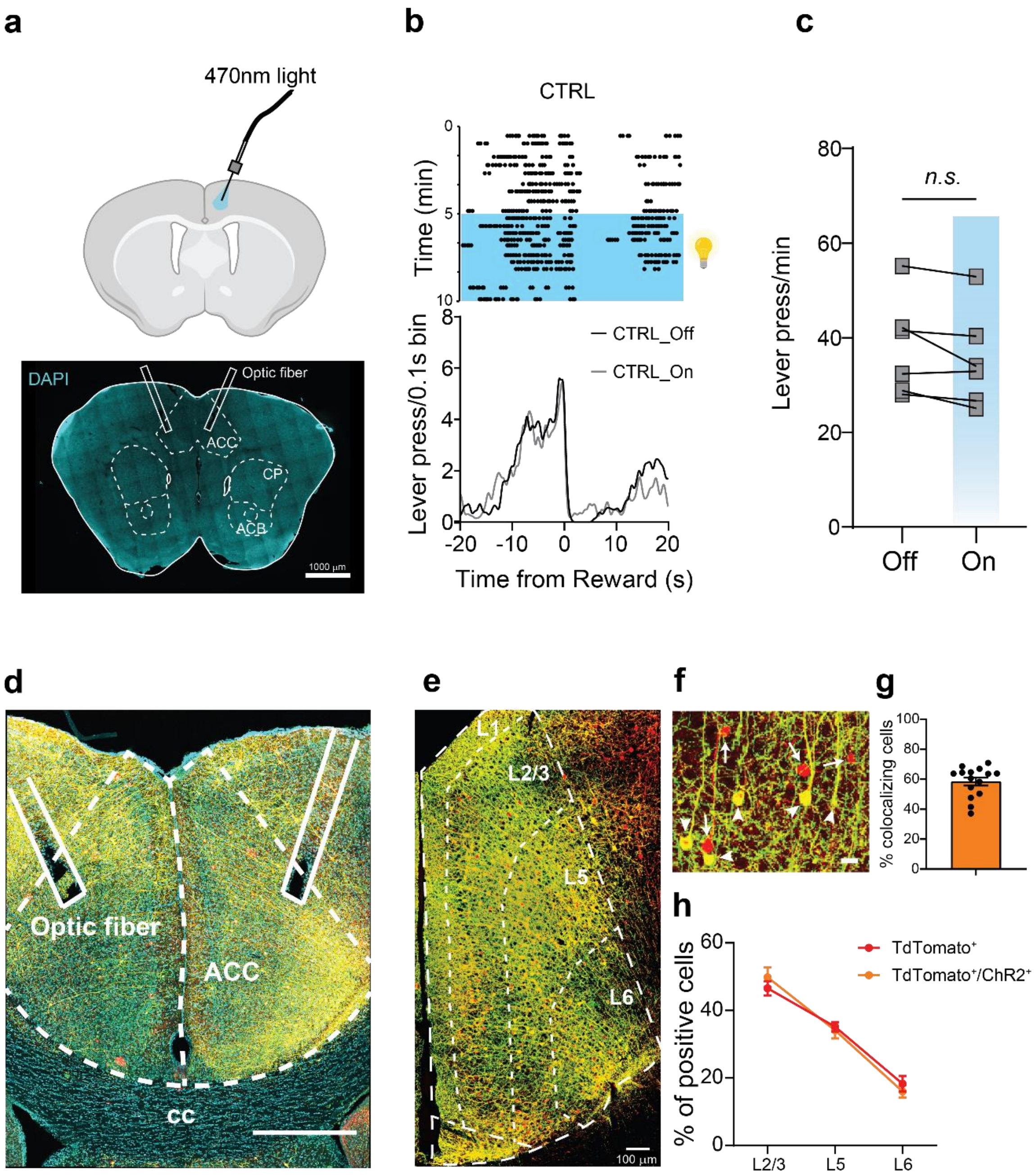

Supplementary Figure 7. Light stimulation in absence of ChR2 does not change the lever-press behavior.
a, Schematic representation of the CTRL mice implanted with optic fibers and without ChR2 viral injection (created with Biorender.com). **b**, Example peri-reward raster histogram during time of light-Off and light-On. **c**, Lever press/min for CTRL mice (n = 6) during light-Off (38 ± 4.2) and light-On (35 ± 4.2). *Paired Two-tailed t-test [t (5) = 2.2, p = 0.079]*. **d**, Anatomical confirmation of viral expression and fiber placement in a representative example of an α2δ-1(f/f) mouse expressing the Rox-Cre and the Cre-dependent ChR2 in $ACC_{\rightarrow DMS}$ neurons. **e**, Representative image of $ACC_{\rightarrow DMS}$ neurons across layers positive for tdTomato and ChR2. **f**, Magnified image of $ACC_{\rightarrow DMS}$ neurons labeled by tdTomato (arrow) and ChR2, yellow neurons (arrow heads) are the ones expressing both molecules. **g**, Quantification of colocalized cells within the ACC (h) Distribution of $tdTomato^{+}$ and $tdTomato/ChR2^{+}$ cells across layers. For all graphs: Data shown as mean ± s.e.m. alpha = 0.05. Source data are provided as a Source Data file.

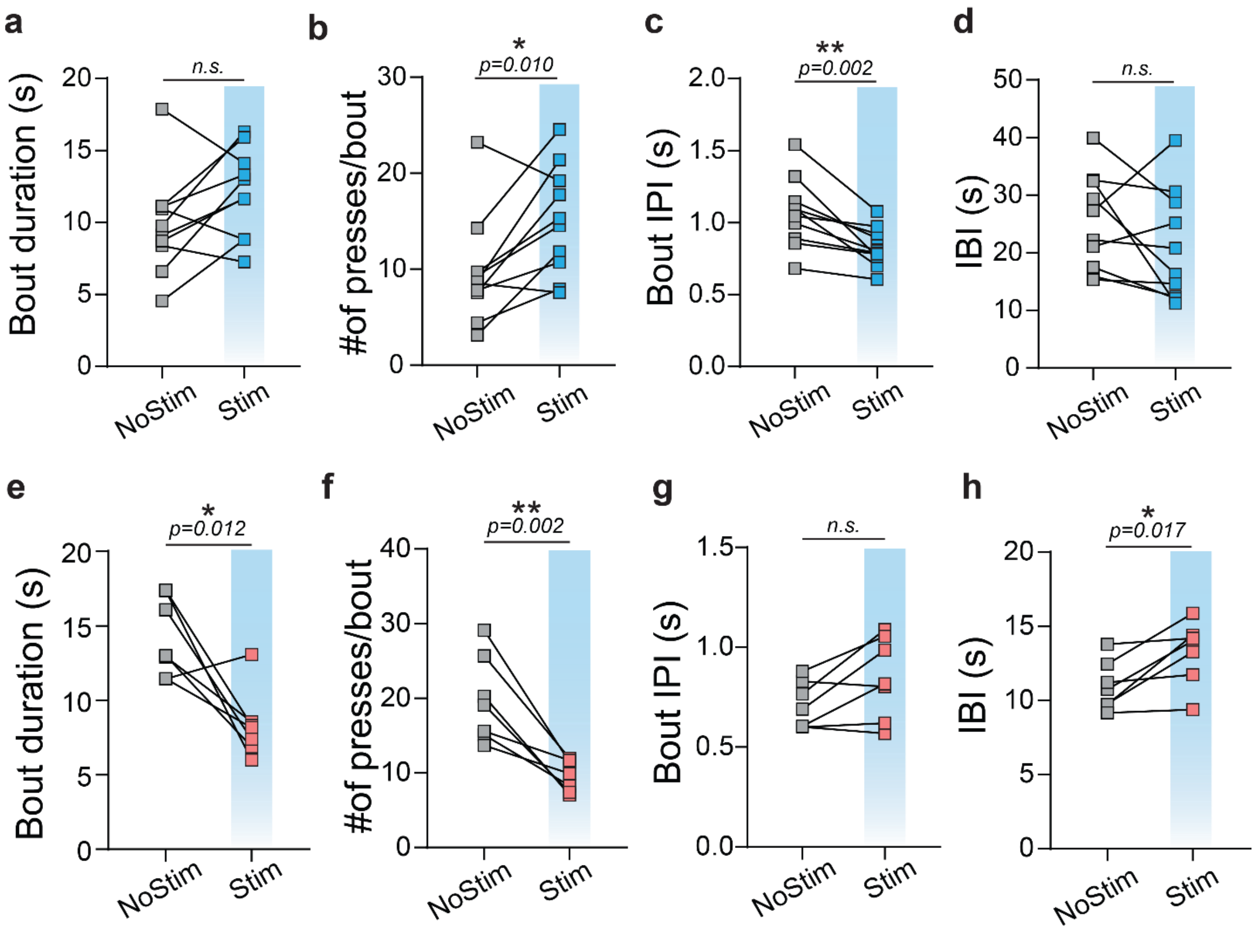

Supplementary Figure 8. Optogenetic modulation of ACC$_{\rightarrow DMS}$ neurons changes the lever press bout properties.

a, Bout duration in NoStim (9.8 ±1.1) and Stim (12 ± 0.96) conditions were not significantly different. *Paired Two-tailed t-test [t (9) = 2.0, p = 0.074].* **b**, Number of presses per bout during NoStim (9.7 ± 1.8) and Stim (15 ± 1.8) days showed a significant difference *Paired Two-tailed t-test [t (9) = 3.2].* **c**, Bout IPI during NoStim (1.1 ± 0.07) and Stim (0.8 ± 0.04) showed a significant decrease. *Paired Two-tailed t-test [t (9) = 4.2].* **d**, IBI in NoStim (25 ± 2.6) compared to Stim (21 ± 3.0) showed no difference. *Paired Two-tailed t-test [t (9) = 1.4, p = 0.186]. For graphs in a-d: n = 10 mice.* **e**, Bout duration in NoStim (14 ±1.0) and Stim (8.3 ± 0.86) conditions were significantly different. *Paired Two-tailed t-test. [t (6) = 3.6].* **f**, Number of presses per bout during NoStim (20 ± 2.2) and Stim (9.7 ± 0.79) days showed a significant difference. *Paired Two-tailed t-test [t (6) = 5.0]*. **g**, Bout IPI during NoStim (0.71 ± 0.04) and Stim (0.85 ± 0.08) showed no significant difference*. Paired Two-tailed t-test [t (6) = 2.4, p = 0.051].* **h**, IBI in NoStim (11 ± 0.63) compared to Stim (13 ± 0.80) showed a significant difference. *Paired Two-tailed t-test [t (6) = 3.3]. For graphs in e-h: n = 7 mice.* Source data are provided as a Source Data file.

Supplementary Table 1. Extended names for the acronyms used to identify the brain regions.

Cerebral cortex	ACC	Anterior Cingulate Cortex
	AI	Agranular Insuar area
	BMA	Basomedial amygdalar nucleus
	CLA	Claustrum
	COA	Cortical amygdalar area
	EP	Endopiriform nucleus
	GU	Gustatory area
	ILA	Infralimbic Area
	MOp	Primary motor Area
	MOs	Secondary motor Area
	ORB	Orbital area
	PIR	Piriform area
	PL	Prelimbic area
	RSP	Retrosplenial area
	SSp	Primary somatosensory area
	SSs	Secondary somatosensory area
	TT	Taenia tecta
	VIS	Visual areas
Cerebral nuclei	AAA	Anterior amygdalar area
	ACB	Nucleus accumbens
	CP	Caudatum putamen
	FS	Fundus of striatum
	GPe	Globus pallidus external segment
	isl	Islands of Calleja
	LS	Lateral septal nucleus
	MA	Magnocellular nucleus
	MS	Medial septal nucleus
	NDB	Diagonal band nucleus
	OT	Olfactory tubercle
	sAMY	Striatum-like amygdalar nuclei
	SF	Septofimbrial nucleus
	SH	Septohippocampal nucleus
	SI	Substantia innominata
	TRS	Triangular nucleus of septum
Interbrain	ADP	Anterodorsal preoptic nucleus
	HY	Hypothalamus
	LPO	Lateral preoptic area
	MPO	Medial preoptic area
	PT	Parataenial nucleus
	PVT	Paraventricular nucleus of the thalamus
	SCH	Suprachiasmatic nucleus
	TH	Thalamus
