# Supplementary material for: Training-induced circuit-specific excitatory synaptogenesis in mice is required for effort control
Source: Nat Commun. 2023 Sep 8;14:5522. doi: 10.1038/s41467-023-41078-z (PMC10491649; doi:10.1038/s41467-023-41078-z)
Supplement: Supplementary file 2 — Reporting Summary [file 41467_2023_41078_MOESM2_ESM.pdf]

nature portfolio

Corresponding author(s): Cagla Eroglu, Henry Yin, Francesco Paolo Ulloa Severino

Last updated by author(s): Jul 25, 2023

Reporting Summary

Nature Portfolio wishes to improve the reproducibility of the work that we publish. This form provides structure for consistency and transparency in reporting. For further information on Nature Portfolio policies, see our Editorial Policies and the Editorial Policy Checklist.

Statistics

For all statistical analyses, confirm that the following items are present in the figure legend, table legend, main text, or Methods section.

n/a	Confirmed	
☐	☒	The exact sample size (*n*) for each experimental group/condition, given as a discrete number and unit of measurement
☐	☒	A statement on whether measurements were taken from distinct samples or whether the same sample was measured repeatedly
☐	☒	The statistical test(s) used AND whether they are one- or two-sided<br>*Only common tests should be described solely by name; describe more complex techniques in the Methods section.*
☒	☐	A description of all covariates tested
☐	☒	A description of any assumptions or corrections, such as tests of normality and adjustment for multiple comparisons
☐	☒	A full description of the statistical parameters including central tendency (e.g. means) or other basic estimates (e.g. regression coefficient) AND variation (e.g. standard deviation) or associated estimates of uncertainty (e.g. confidence intervals)
☐	☒	For null hypothesis testing, the test statistic (e.g. F, t, r) with confidence intervals, effect sizes, degrees of freedom and P value noted<br>*Give P values as exact values whenever suitable.*
☒	☐	For Bayesian analysis, information on the choice of priors and Markov chain Monte Carlo settings
☒	☐	For hierarchical and complex designs, identification of the appropriate level for tests and full reporting of outcomes
☒	☐	Estimates of effect sizes (e.g. Cohen's *d*, Pearson's *r*), indicating how they were calculated

Our web collection on statistics for biologists contains articles on many of the points above.

Software and code

Policy information about availability of computer code

Data collection	Med-PC-IV; Central blackrock microsystems; Bonsai 2.6.3; Olympus Fluoview; Patchmaster (HEKA); pCLAMP10;
Data analysis	GraphPad Prism v8 and v9; Matlab R2018a, R2022b; Neuroexplorer v5; WholeBrain 1.0; UNet 2015; Trimmomatic v0.38; Bowtie2 v2.3.5.1; Subread featureCounts v1.6.3; edgeR v3.30.3; ImageJ 1.53c; Minhee Analysis software v1.1.3; Cluster Profile v4.6.2; Stitching and segmentation custom code https://github.com/ErogluLab/CellCounts; custom scripts available at https://github.com/UlloaSeverinoLab

For manuscripts utilizing custom algorithms or software that are central to the research but not yet described in published literature, software must be made available to editors and reviewers. We strongly encourage code deposition in a community repository (e.g. GitHub). See the Nature Portfolio guidelines for submitting code & software for further information.

Data

Policy information about availability of data

All manuscripts must include a data availability statement. This statement should provide the following information, where applicable:

- Accession codes, unique identifiers, or web links for publicly available datasets
- A description of any restrictions on data availability
- For clinical datasets or third party data, please ensure that the statement adheres to our policy

Further information and requests for resources and reagents can be directed to the Lead Contacts, Francesco Paolo Ulloa Severino (francesco.ulloa@duke.edu),

Henry Yin (hy43@duke.edu), and Cagla Eroglu (cagla.eroglu@duke.edu). The reagents and data generated in this study are available without restriction. RNA-sequencing data have been deposited in the Gene Expression Omnibus (GEO) repository with accession number: GSE169392. The accession code for the mouse genome used for the RNAseq is GRCm38 available here https://www.ncbi.nlm.nih.gov/datasets/genome/GCF_000001635.20/. The scRNAseq database from Saunders et al., 2018 can be found here http://dropviz.org/.

Research involving human participants, their data, or biological material

Policy information about studies with human participants or human data. See also policy information about sex, gender (identity/presentation), and sexual orientation and race, ethnicity and racism.

Reporting on sex and gender	N/A
Reporting on race, ethnicity, or other socially relevant groupings	N/A
Population characteristics	N/A
Recruitment	N/A
Ethics oversight	N/A

Note that full information on the approval of the study protocol must also be provided in the manuscript.

Field-specific reporting

Please select the one below that is the best fit for your research. If you are not sure, read the appropriate sections before making your selection.

☒ Life sciences ☐ Behavioural & social sciences ☐ Ecological, evolutionary & environmental sciences

For a reference copy of the document with all sections, see nature.com/documents/nr-reporting-summary-flat.pdf

Life sciences study design

All studies must disclose on these points even when the disclosure is negative.

Sample size	Sample size for each experiment is indicated in the figure legend for each experiment. The sample size for each experiments was chosen based on previous experience (Risher et al., 2018; Eroglu et al., 2009; Singh et al., 2016; Yin et al., 2009; Kim et al., 2019;). No statistical methods were used to predetermine sample size.
Data exclusions	No samples were excluded from any reported experiments and analysis, except for the cases reported in the following points:<br>1)Three animals were excluded from the behavioral analysis of the BLINK2 experiments based on the lack of viral expression.
Replication	Each experiment was performed at least three times and all findings were reliably reproducible.
Randomization	For in vivo studies, animals were chosen based on correct genotypes, requiring 2 or 3 correct alleles. Each experiment contained animals from at least three different litters to ensure that the differences between genotypes can be observed in mice from different litters. Sex-specific differences were addressed by including equal numbers of both sexes for all animal studies. Once the sex and genotype was assessed, mice were randomly allocated to the experimental groups (i.e., untrained vs trained)
Blinding	For all the synaptic structural analysis the investigator was blind to the experimental conditions to avoid bias in the analysis. For behavioral experiments the group allocation was not relevant as there is no intervention from the experimenter in the collection of the behavioral time stamps. All other data collections and analysis were automatized so blinding was not necessary.

Reporting for specific materials, systems and methods

We require information from authors about some types of materials, experimental systems and methods used in many studies. Here, indicate whether each material, system or method listed is relevant to your study. If you are not sure if a list item applies to your research, read the appropriate section before selecting a response.

Materials & experimental systems

n/a	Involved in the study
☐	☒ Antibodies
☐	☒ Eukaryotic cell lines
☒	☐ Palaeontology and archaeology
☐	☒ Animals and other organisms
☒	☐ Clinical data
☒	☐ Dual use research of concern
☒	☐ Plants

Methods

n/a	Involved in the study
☒	☐ ChIP-seq
☒	☐ Flow cytometry
☒	☐ MRI-based neuroimaging

Antibodies

Antibodies used	Antibodies used for immunohistochemistry:<br>1) Rabbit anti-cFos (Calbiochem, PC05) - Immunofluorescence<br>2) Donkey anti-rabbit conjugated to Alexa fluor-594<br>3) Guinea pig anti-VGlut1 (AB5905, Millipore, MA)<br>4) Rabbit anti-PSD95 (51–6900, Invitrogen, CA)<br>5) Guinea pig anti-VGAT (Synaptic Systems 131 004)<br>6) Rabbit anti-Gephyrin (Synaptic Systems 147 002)<br>7) Rabbit anti-RFP (Rockland, 600-401-379)<br>8) Chicken anti-GFP (Millipore, AB16901; Aveslabs, GFP 1010)
Validation	1) Validation: Manufacturer- https://www.emdmillipore.com/US/en/product/Anti-c-Fos-Ab-2-4-17-Rabbit-pAb,EMD_BIO-PC05<br>2) Validation: Manufacturer - https://www.thermofisher.com/antibody/product/Donkey-anti-Rabbit-IgG-H-L-Highly-Cross-Adsorbed-Secondary-Antibody-Polyclonal/A-21207<br>3) Validation: Manufacturer - https://www.emdmillipore.com/US/en/product/Anti-Vesicular-Glutamate-Transporter-1-Antibody,MM_NF-AB5905<br>4) Validation: Manufacturer - https://www.thermofisher.com/antibody/product/PSD-95-Antibody-Polyclonal/51-6900<br>5) Validation: Manufacturer - https://sysy.com/product/131004<br>6) Validation: Validated in house using co-staining with other anti-Gephyrin antibodies.<br>7) Validation: Manufacturer https://www.thermofisher.com/antibody/product/RFP-Pre-adsorbed-Antibody-Polyclonal/600-401-379<br>8) Validation: Validated in house using tissue sections not injected with GFP expressing constructs.

Eukaryotic cell lines

Policy information about cell lines and Sex and Gender in Research

Cell line source(s)	Human Embryonic kidney cells 293T. Purchased from American type culture collection (ATCC) #CRL-11268
Authentication	The cell line used in this manuscript have not been authenticated.
Mycoplasma contamination	We confirmed that all cell lines were negatively tested for mycoplasma.
Commonly misidentified lines<br>(See ICLAC register)	No commonly misidentified lines were used in this work.

Animals and other research organisms

Policy information about studies involving animals; ARRIVE guidelines recommended for reporting animal research, and Sex and Gender in Research

Laboratory animals	All the studies were conducted on adult mice (3-5months) with strain C57BL/6J.<br>Wilde type - The Jackson laboratory - Strain #000664<br>B6.Cg-Gt(ROSA)26Sortm14(CAG-tdTomato)Hze/J - The Jackson laboratory - Strain #007914<br>a2d-1 Het, KO, and conditional KO - Generated from our laboratory<br><br>All animals, were kept under typical day/night conditions of 12-hours cycles. The room temperature is set at 22 degrees Celsius and humidity around 50%.
Wild animals	No wild animals were used.
Reporting on sex	All the behavioral experiments were conducted by including both male and female mice. The analysis of sex differences was carried for the initial behavioral experiments and then stopped based on the findings that both sexes, independently from their genotype, were behaving similarly. The data are reported in our supplementary figures 1 and 4.
Field-collected samples	No field collected samples were used.

Ethics oversight	The Institutional Animal Care and Use Committee (IACUC) and the Duke Division of Laboratory Animal Resources (DLAR).

Note that full information on the approval of the study protocol must also be provided in the manuscript.
